# Multiomics analysis of platelet-rich plasma promoting biological performance of mesenchymal stem cells

**DOI:** 10.1186/s12864-024-10329-8

**Published:** 2024-06-05

**Authors:** Pengxiu Dai, Yi Wu, Yaxin Gao, Mengnan Li, Mingde Zhu, Haojie Xu, Xiancheng Feng, Yaping Jin, Xinke Zhang

**Affiliations:** The College of Veterinary Medicine, Northwest A and F University, Yangling, 712100 Shaanxi China

**Keywords:** Platelet-rich plasma, Mesenchymal stem cells, Biological performance, Mechanism

## Abstract

Mesenchymal Stem Cells are ideal seed cells for tissue repair and cell therapy and have promising applications in regenerative medicine and tissue engineering. Using Platelet-Rich Plasma as an adjuvant to create and improve the microenvironment for Mesenchymal Stem Cells growth can enhance the biological properties of Mesenchymal Stem Cells and improve the efficacy of cell therapy. However, the mechanism by which Platelet-Rich Plasma improves the biological performance of Mesenchymal Stem Cells is still unknown. In this study, by examining the effects of Platelet-Rich Plasma on the biological performance of Mesenchymal Stem Cells, combined with multiomics analysis (Transcriptomics, Proteomics and Metabolomics) and related tests, we analyzed the specific pathways, related mechanisms and metabolic pathways of Platelet-Rich Plasma to improve the biological performance of Mesenchymal Stem Cells. In an *in vitro* cell culture system, the biological performance of Mesenchymal Stem Cells was significantly improved after replacing Foetal Bovine Serum with Platelet-Rich Plasma, and the genes (*ESM1*, *PDGFB*, *CLEC7A*, *CCR1* and *ITGA6 et al.*) related to cell proliferation, adhesion, growth, migration and signal transduction were significantly upregulated. Platelet-Rich Plasma can enhance the secretion function of MSC exosomes, significantly upregulate many proteins related to tissue repair, immune regulation and anti-infection, and enhance the repair effect of exosomes on skin injury. After replacing Foetal Bovine Serum with Platelet-Rich Plasma, Mesenchymal Stem Cells underwent metabolic reprogramming, the metabolism of amino acids and fatty acids and various signaling pathways were changed, the anabolic pathways of various proteins were enhanced. These results provide a theoretical and technical reference for optimizing the Mesenchymal Stem Cells culture system, improving the biological characteristics and clinical application effects of Mesenchymal Stem Cells.

**Supplementary Information:**

The online version contains supplementary material available at 10.1186/s12864-024-10329-8.

## Introduction

Mesenchymal stem cells (MSCs) come from a wide range of sources, express a variety of surface markers such as CD105 and CD73, and have good adhesion and migration ability, multiple differentiation potential, immune regulatory functions and the ability to promote the repair of damaged tissues. It is an ideal seed cell for cell transplantation therapy and tissue engineering [1, 2]. At the same time, MSCs can secrete exosomes through paracrine action, and MSCs exosomes are more safe, stable and efficient, and have stronger and complex signaling molecule transport and regulation capabilities. More and more studies suggest that MSCs exosomes may play a key role in tissue damage repair. Exosomes carrying signals such as cytokines and nucleic acids can be transmitted to neighboring target cells to promote the repair of damaged sites [3, 4]. In clinical applications, the survival, proliferation and continuous exosome secretion of MSCs after implantation are the keys to successful cell therapy. However, due to various factors, a large number of cells will die after implantation at the injured site [5], and the exosome secretion function of the remaining cells will be significantly weakened.

Therefore, how to improve the biological performance of MSCs and improve the survival rate and exosome secretion function after transplantation has become the focus and challenge of current research. Many studies have attempted to improve the biological properties of MSCs through gene modification or drug assistance to optimize the therapeutic effect [6, 7]. Although these optimization methods are effective, they are difficult to translate into clinical applications in most cases, and the biological properties of MSCs need to be significantly improved through other methods to improve the clinical therapeutic effect based on MSCs.

Platelet-rich plasma (PRP) is rich in a variety of growth factors and extracellular vesicles, which can promote the proliferation and migration of MSCs, reduce cell apoptosis, necrosis and senescence, and improve their survival rate under oxidative stress [7, 8]. Therefore, we suggest the combined application of PRP and MSCs, using PRP as an adjuvant to create and improve the microenvironment for the growth of MSCs, enhance the biological performance of MSCs, and thus improve the clinical efficacy to achieve the effect of 1+1 > 2 (synergistic effect). Hosni Ahmed *et al.* found that after pretreating MSCs with PRP, cell proliferation was accelerated and TGF-β secretion was increased. In burn treatment, after pretreating MSCs with PRP, the expression of ANG-1, ANG-2 and VIMENTIN was upregulated, while the expression of MMP-1 and TIMP-2 was downregulated, and the healing time was significantly reduced [9]. Sassoli *et al.* found that PRP combined with MSCs was more effective in skeletal muscle repair than PRP alone or MSCs alone [10]. After treating MSCs with PRP, Myung *et al.* found that the content of vascular endothelial growth factor was higher in the cell medium, and the wound healing rate and angiogenesis were significantly increased when MSCs and PRP were combined to treat radiation-induced wounds in mice [11].

Currently, most studies have confirmed that the clinical effect of cell therapy can be greatly improved after the combination of PRP and MSCs or the pretreatment of MSCs with PRP. However, regarding why PRP can significantly improve the biological performance of MSCs, Levoux *et al.* found that dynamic dependent clathrin-mediated endocytosis can transfer platelet mitochondria to MSCs and promote the proangiogenic activity of MSCs through metabolic remodeling, revealing a new mechanism by which PRP enhances the biological performance of MSCs [12]. In addition, after the treatment of MSCs with PRP, what other components of PRP play a role, what changes occur in the gene expression and metabolic pathways of MSCs, whether other biological properties are significantly improved, and whether PRP can be used as a substitute for serum *in vitro* culture to avoid the risk of transmission of zoonotic diseases remain unclear. These questions require further investigation. In addition, exosomes secreted by mesenchymal stem cells have no immunogenicity and few side effects and can be used in a wide range of applications, making them a promising cell-free therapeutic tool that can overcome many risks and difficulties in cell therapy [13]. What specific changes in exosome secretion function will occur after PRP replaces fetal bovine serum *in vitro* culture or pretreats MSCs with PRP and what significance these changes have for clinical application also need to be further explored and verified.

Therefore, this study planned to take canine adipose-derived mesenchymal stem cells (cADMSCs) as seed cells, start with the detection of the effect of PRP on the biological performance of fat MSCs, conduct multiomics combined analysis of the cells and relevant experimental verification, and analyze the specific pathways, related mechanisms and metabolic pathways by which PRP improves the biological performance of fat MSCs. This study revealed the exosome secretion changes and therapeutic effects of MSCs after PRP replaced fetal bovine serum *in vitro* culture, providing theoretical and technical references for optimizing the MSC culture system, improving the biological performance and clinical application effects of MSCs, and achieving better functional recovery of damaged tissues.

## Methods

### PRP extraction

Adult beagle dogs’ venous (provided by the Laboratory Animal Center of Northwest A & F University) blood was extracted using a negative pressure tube preloaded with heparin sodium anticoagulant (WEGO, 20172410710, China), mixed well, 1 mL of blood was used for whole blood platelet count (Automatic cell analyzer, Mindray, BC-5000, China), and the remaining blood was divided into new centrifuge tubes (5 mL/tube). The blood was centrifuged for the first time (Centrifuge from Xiangyi, HT165R, China), centrifuged at 900 g at 4 ℃ for 5 minutes. The upper layer of pale-yellow plasma and the part 1-2 mm below the plasma layer were absorbed into a new centrifuge tube, centrifuged at 1500 g at 4 ℃ for 15 minutes. The upper 2/3 was discarded and the remaining part, which was called PRP, platelet count (Automatic cell analyzer, Mindray, BC-5000, China) was performed after mixing. The prepared PRP was transferred to a new centrifuge tube, the platelet concentration was adjusted to 1x10^9^/mL using α-MEM basic medium, and the PRP was frozen and thawed 4 times in the -80 ℃ refrigerator and 37 ℃ water bath, and the vortex was carried out after each freeze and thawing. After freezing and thawing, centrifuge at 5000 rpm at 4 ℃ for 30 minutes, and the resulting supernatant is activated PRP. The samples are divided into frozen storage tubes and placed at -80 ℃ for use.

### Cell culture medium

Three types of cell culture medium were configured. PRP Group (α-MEM medium (Procell, PM150421, China) containing 10% activation PRP, 1% 100× Penicillin, Streptomycin Solution (Procell PB180120, China) and 0.25% Anti - Myc mycoplasma clearing reagent (Procell, P-CMR-001, China)). FBS Group (α-MEM culture medium (Procell, PM150421, China) containing 10% exosomal removed fetal bovine serum (ViVaCell, C38010050, Germany), 1% 100× Penicillin Streptomycin Solution (Procell, PB180120, China) and 0.25% Anti-Myc Mycoplasma scavenging reagent (Procell, P-CMR-001, China). CONT Group (α-MEM medium (Procell, PM150421, China) containing 1% 100× Penicillin, Streptomycin Solution (Procell PB180120, China) and 0.25% Anti - Myc mycoplasma clearing reagent (Procell, P-CMR-001, China))

### Cell culture and detection

The lab preserved frozen P3 generation cADMSCs were resuscitated using FBS medium. The isolation, culture and identification of the cells are shown in the previous results [14, 15], and after the cells were attached to the wall, the medium was abandoned, and the cells were cultured through the above medium, respectively, to observe the growth rate and growth morphology of the cells. At the same time, cells were tested for proliferation according to the instructions of Cell Counting Kit-8 (Sigma‒Aldrich, 96992-500TESTS-F, USA). Each well (96-well plate) was added with 10 μL CCK-8 solution, and the culture plate was incubated in the incubator for 1.5 h. The absorbance at 450 nm was measured by enzyme labeling.

Cells with a density of 1000 cells per well were inoculated into 6-well plates (FBS group medium), and after the cells were attached to the wall, the medium was discarded, and the cells were cultured through the above medium, the number of cell colonies was counted (Cell counting apparatus, Countstar, IC1000, China), and the results were analyzed.

The cells were treated with the media of PRP group and FBS group for 48 h, respectively, and then the cell suspension was prepared with the culture medium of fetal bovine serum, and the migration ability of the cells was measured by Transwell (Corning, 3414, China) migration test. In the migration assay, 2 × 10^4^ cells suspended in 100 μL serum-free α-MEM were seeded in the upper compartment of the chamber and 800 μL α-MEM with 10% FBS were added to the lower compartment of the chamber. After 24 h, the migrated cells were counted.

The cells were cultured using FBS culture medium, and after the cells were adhered to the walls, the culture was abandoned, and the migration ability of the cells was evaluated through cell scratch experiment. A parallel line was drawn in the petri dish using a 10 μL pipette, and the cell debris were gently rinsed with PBS, and the medium of the FBS group and PRP group was added respectively. The cell migration was observed at 48 h.

At the same time, Staining was detected according to the instructions of Senescence β-Galactosidase Staining Kit (Beyotime, C0602, China). The cell culture medium was sucked out, the cells were washed once with PBS, 1 mL of staining fixing solution was added, and fixed at room temperature for 15 minutes. After the fixative was removed, the cells were washed with PBS 3 times for 3 minutes each time. Add 1 mL SA-β-gal staining solution to each well. Incubate overnight at 37 ° C and cover with plastic wrap to prevent evaporation. The cells were observed under an ordinary light microscope.

### Transcriptome sequencing

CONT Group, FBS Group and PRP Group were used to culture the cADMSCs for 48 h, discard the media, rinse with PBS 3 times, collect the cells, and perform transcriptome sequencing analysis. Total RNA was extracted using TRIzol reagent (Thermofisher, 15596018, USA) following the manufacturer's procedure. The total RNA quantity and purity were analysis of Bioanalyzer 2100 and RNA 6000 Nano LabChip Kit (Agilent, 5067-1511, USA), high-quality RNA samples with 2.0 > A260/280 ratio > 1.8, RIN > 7.0 were used to construct sequencing library. After total RNA was extracted, mRNA was purified from total RNA (5 µg) using Dynabeads Oligo (dT) (Thermo Fisher, 61002, USA) with two rounds of purification. Following purification, the mRNA was fragmented into short fragments using divalent cations. Then, the cleaved RNA fragments were reverse-transcribed to create the cDNA by SuperScript™ II Reverse Transcriptase (Invitrogen, 1896649, USA), which were then used to synthesize U-labeled second-stranded DNAs with *E. coli* DNA polymerase I (NEB, m0209, USA), RNase H (NEB, m0297, USA) and dUTP Solution (Thermo Fisher, R0133, USA). An A-base was then added to the blunt ends of each strand, preparing them for ligation to the indexed adapters. Each adapter contained a T-base overhang for ligating the adapter to the A-tailed fragmented DNA. Dual-index adapters were ligated to the fragments, and size selection was performed with AMPureXP beads. After the heat-labile UDG enzyme (NEB, m0280, USA) treatment of the U-labeled second-stranded DNAs, the ligated products were amplified with PCR. The average insert size for the final cDNA libraries were 300±50 bp. Finally, we performed the 2×150 bp paired-end sequencing (PE150) on an Illumina Novaseq™ 6000 (LC-Bio Technology CO., Ltd., Hangzhou, China) following the vendor's recommended protocol.

### Bioinformatics Analysis of transcriptome sequencing data

Reads obtained from the sequencing machines include raw reads containing adapters or low-quality bases, which will affect the subsequent assembly and analysis. Thus, to obtain high-quality clean reads, reads were further filtered by Cutadapt (https://cutadapt.readthedocs.io/en/stable/, version:cutadapt-1.9). The parameters were as follows: 1) removing reads containing adapters; 2) removing reads containing polyA and polyG; 3) removing reads containing more than 5% of unknown nucleotides (N); and 4) removing low-quality reads containing more than 20% of low-quality (Q-value≤20) bases. Then, sequence quality was verified using FastQC (http://www.bioinformatics.babraham.ac.uk/projects/fastqc/, 0.11.9) [16–18]. After that, a total of G bp of cleaned, paired-end reads were produced.

We aligned reads of all samples to the reference genome using the HISAT2 (https://daehwankimlab.github.io/hisat2/, version:hisat2-2.2.1) package, which initially removed a portion of the reads based on quality information accompanying each read and then mapped the reads to the reference genome [19–21]. The mapped reads of each sample were assembled using StringTie (http://ccb.jhu.edu/software/stringtie/, version:stringtie-2.1.6) with default parameters. Then, all transcriptomes from all samples were merged to reconstruct a comprehensive transcriptome using gffcompare software (http://ccb.jhu.edu/software/stringtie/gffcompare.shtml, version:gffcompare-0.9.8). After the final transcriptome was generated, StringTie and Ballgown (http://www.bioconductor.org/packages/release/bioc/html/ballgown.html) were used to estimate the expression levels of all transcripts and perform expression abundance for mRNAs by calculating FPKM (fragment per kilobase of transcript per million mapped reads) values [21–23].

Gene differential expression analysis was performed by DESeq2 software between two different groups (and by edgeR between two samples). Genes with a false discovery rate (FDR) below 0.05 and absolute fold change ≥ 2 were considered differentially expressed genes. Differentially expressed genes were then subjected to enrichment analysis of GO functions and KEGG pathways [24, 25].

GO has three ontologies: molecular function, cellular component and biological process. First, all DEGs were mapped to GO terms in the Gene Ontology database (http://www.geneontology.org/), gene numbers were calculated for every term, and significantly enriched GO terms in DEGs compared to those in the genome background were defined by a hypergeometric test [26]. The calculation formula of GO terms meeting this condition with p < 0.05 was defined as significantly enriched GO terms in DEGs. This analysis was able to recognize the main biological functions of the DEGs [27].

Genes usually interact with each other to play roles in certain biological functions. Pathway-based analysis helps to further understand gene biological functions. KEGG (https://www.kegg.jp/kegg/) is the major public pathway-related database. Pathway enrichment analysis identified significantly enriched metabolic pathways or signal transduction pathways in DEGs compared with those in the whole genome background [28]. Pathways meeting this condition with p < 0.05 were defined as significantly enriched pathways in DEGs.

### TMT labeled quantitative proteomics

The cADMSCs were cultured for 48 h using the medium of the FBS group and PRP group, and the supernatant of the cell culture was collected, centrifuged at 5000 g for 10 min, and the supernatant was collected (repeated twice). Exosomes were extracted from the collected supernatant and activated PRP (BP group). The sample was moved to a new centrifuge tube and centrifuged at 2000 g at 4 ℃ for 30 min. Carefully move the supernatant into a new centrifuge tube, 10000 g, 4 ℃, 45 min and centrifuge again to remove large vesicles. The supernatant was filtered by 0.45 μm filter membrane, and the filtrate was collected. The filtrate was transferred to a new centrifuge tube, the overspeed rotor was selected, and centrifuged at 100000 g at 4 ℃ for 70 min. After the supernatant was removed and re-suspended with 100 mL pre-cooled 1×PBS, the overspeed rotor was selected and centrifuged again at 4 ℃, 100000 g, for 70 min. The supernatant was removed and the exosomes were suspended with 100 μL pre-cooled 1×PBS, and the exosomes were preserved at -80 ℃.

The extracted exosomes were extracted for protein extraction, and quality control of the extracted proteins was carried out. A proper number of samples were weighed and transferred into 2 mL centrifuge tubes. Steel balls, lysis solution containing 8M Urea/50mMTris-HCL and Roche cocktail with 1X final concentration were added into the centrifuge tubes. The tubes were placed on ice for 5 min. Samples were homogenated using a tissue lyser (60 Hz, 2 min) followed by 15 min centrifugation (20,000 g, 4 °C). The supernatant was collected.

DTT was added to obtain a final concentration of 10 mM, and the tubes were placed in 37℃ water bath for 1 hour. IAA was added to obtain a final concentration of 20 mM, and the tubes were placed away from light for 30 min. The Bradford method for protein quantitation 0, 2, 4, 6, 8, 12, 14, 16, 18μL standard protein solution (0.2 μg/μL BSA) was added into the 96-well plate successively, and then 20, 18, 16, 14, 12, 10, 8, 6, 4, 2 μL pure water was added successively. After mixing thoroughly, 180μL Coomas bright Blue G-250 quantitative working solution was added to each well. A microplate reader was used to measure the absorption value at 595nm (OD595), and the linear standard curve was created according to OD595 and protein concentration. 180 μL quantitative working solution was added to 20 μL protein solution of the sample, and the OD595 of the sample was read. The protein concentration of the sample was calculated according to the standard curve and OD595 of the sample.

A total of 150 μg of protein was taken from each sample. Three micrograms of trypsin was added to obtain a protein:enzyme ratio of 50:1, and the samples were incubated for 14-16 h at 37 °C. The enzymatically digested peptides were desalted using Waters solid phase extraction cartridges and then vacuum dried. The dried peptide fractions were redissolved in pure water and stored at -20 °C [29].

A proper amount of peptide was taken from each sample and then redissolved to 30 μL with 100 mM TEAB after vacuum drying. Each TMT-10plex label was dissolved in 100% anhydrous acetonitrile. Each sample was added to each TMT label at a ratio of TMT label:peptide = 5:1 and left to stand for 1-2 hours at room temperature [29].

Equal amounts of peptide from each sample were mixed, diluted with solvent A (5% ACN, pH 9.8) and injected into the column. The peptide mixture was fractionated using a 3.5 μm 4.6x150 mm Agilent ZORBAX 300Extend- C18 column on a Thermo Scientific UltiMate™ 3000 Binary Rapid Separation System. The gradient elution was performed at a flow rate of 0.3 mL/min: 5 to 21% solvent B (97% ACN, pH 9.8) in 38 min, 21.5% to 40% solvent B in 20 min, 40% to 90% solvent B in 2 min, 90% solvent B for 3 min, and 5% solvent B equilibrated for 10 min. The elution peaks were monitored at 214 nm, and fractions were collected every minute. The fractions were combined according to chromatograms of the elution peaks. Ten fractions were obtained and then freeze-dried [30].

The dried peptide samples were redissolved with 0.1% FA followed by 10 min of centrifugation (20,000 g). The supernatant was collected and injected into a self-loading C18 column (100 μm I.D., 1.8 μm column media particle size, approximately 35 cm column length). Separation was performed by a Thermo Scientific EASY-nLC™ 1200 system at a flow rate of 300 nL/min through the following effective gradient: From 0 to 48 min, 8% solvent B (98% ACN, 0.1% FA) linearly increased to 32%; from 48-53 min, solvent B increased from 32% to 45%; from 53-555 min, solvent B increased from 45% to 90%; and from 55-662 min, 90% solvent B. The separated peptides were ionized by nano-ESI and then transferred to an Orbitrap Fusion mass spectrometer (Thermo Fisher Scientific, San Jose, CA) for DDA mode detection. Parameter settings: ion source voltage was 2.2 KV; scan range of primary mass spectrometry was 350~1,500 m/z; resolution was 120,000; normalized AGC target was 300%. The secondary mass spectrometry fragmentation mode was HCD. The fragmentation energy was set at 36%; the resolution was set at 45,000; the dynamic exclusion time was 60 s. The starting m/z of secondary mass spectrometry was fixed to 110; the parent ion screening condition for secondary fragmentation was charge 2+ to 6+; the normalized AGC target was set at 200%, and the maximum ion injection time (MIT) was 105 ms[31].

### Protein Identification and Quantification

MaxQuant (https://www.maxquant.org/) (version 2.1.4.0) software was used to analyze the TMT-plexed MS/MS raw data with the following settings: Type: Reporter ion MS2: TMT6plex, TMT10plex, TMT16plex or TMT18plex; enzyme: Trypsin/P; maximum missed cleavages: 2; fixed modification: carbamidomethyl (C); variable modifications: oxidation (M) and acetyl (protein N-term); precursor mass tolerance: 20 ppm; fragment mass tolerance: 0.05 Da; match between runs and second peptide search was enabled. All other parameters are in default. The MS/MS data were searched for protein sequences that were downloaded from the UniProt database. The FDR threshold was set as 1% at both the PSM (peptide spectrum match) and protein levels. Protein from the contaminant or reverse will be removed [29, 30].

Statistical analysis was performed in R (version 4.0.0). The raw protein intensity was normalized by the method "medium", and hierarchical clustering was performed using the pheatmap package. Principal component analysis (PCA) was performed using the metaX package. A t test was used for statistical differential analysis, and a cutoff of p value <= 0.05 and fold change >= 1.2 was used to select statistically differentially expressed proteins. Hypergeometric-based enrichment analysis with KEGG pathway, Gene Ontology and Reactome pathway analyses was performed to annotate protein sequences. Individually [30, 32]. Subcellular localization analysis was performed by WoLF PSORT (https://wolfpsort.hgc.jp/). Transcription factor annotation was based on AnimalTFDB/PlantTFDB.

### Metabolite extraction and mass spectrometry analysis

The FBS group and PRP group were used to culture the cADMSCs for 48 h, the media were discarded, the cells were rinsed with PBS 3 times, and the cells were collected on ice. First, metabolites were extracted from the cells using the organic reagent precipitation protein method. The collected samples were thawed on ice, and metabolites were extracted with 80% methanol buffer. Briefly, 50 mg of sample was extracted with 0.5 mL of precooled 80% methanol. The extraction mixture was then stored for 30 min at -20 °C. After centrifugation at 20,000 g for 15 min, the supernatants were transferred into new tubes and vacuum dried. The samples were redissolved in 100 μL of 80% methanol and stored at -80 °C prior to LC‒MS analysis. In addition, pooled QC samples were also prepared by combining 10 μL of each extraction mixture [33]. All samples were acquired by the LC‒MS system following the manufacturer’s instructions. First, all chromatographic separations were performed using an UltiMate 3000 UPLC System (Thermo Fisher Scientific, Germany). An ACQUITY UPLC T3 column (100 mm*2.1 mm, 1.8 μm, Waters, Milford, USA) was used for the reversed-phase separation. The column oven was maintained at 40 ℃. The fter, (5 mM ammonium acetate and 5 mM acetic acid) and solvent B (acetonitrile). The low rate was 0.3 mL/min, and the mobile phase consisted of solvent A. The gradient elution conditions were set as follows: 0~0.8 min, 2% B; 0.8~2.8 min, 2% to 70% B; 2.8~5.6 min, 70% to 90% B; 5.6~6.4 min, 90% to 100% B; 6.4~8.0 min, 100% B; 8.0~8.1 min, 100% to 2% B; and 8.1~10 min, 2% B [34].

A high-resolution tandem mass spectrometer Q Exactive (Thermo Scientific) was used to detect metabolites eluted from the column. The Q Exactive was operated in both positive and negative ion modes. Precursor spectra (70–1050 m/z) were collected at 70,000 resolution to hit an AGC target of 3e6. The maximum injection time was set to 100 ms. A top 3 configuration to acquire data was set in DDA mode. Fragment spectra were collected at 17,500 resolution to hit an AGC target of 1e5 with a maximum injection time of 80 ms. To evaluate the stability of the LC‒MS during the whole acquisition, a quality control sample (pool of all samples) was acquired after every 10 samples [34, 35].

### Information analysis of metabolic data

Proteowizard's MSConvert software was used to convert the original data of the mass spectrum into readable data mzXML. The acquired MS data pretreatments, including peak picking, peak grouping, retention time correction, second peak grouping, and annotation of isotopes and adducts, were performed using XCMS software. LC−MS raw data files were converted into mzXML format and then processed by the XCMS, CAMERA and metaX toolboxes implemented with R software. Each ion was identified by combining retention time (RT) and m/z data. The intensities of each peak were recorded, and a three-dimensional matrix containing arbitrarily assigned peak indices (retention time-m/z pairs), sample names (observations) and ion intensity information (variables) was generated [35].

The online KEGG and HMDB databases were used to annotate the metabolites by matching the exact molecular mass data (m/z) of samples with those from the database. If a mass difference between the observed and database values was less than 10 ppm, the metabolite was annotated, and the molecular formula of the metabolite was further identified and validated by isotopic distribution measurements. We also used an in-house fragment spectrum library of metabolites to validate the metabolite identification[35, 36].

The intensity of the peak data was further preprocessed by metaX. Those features that were detected in less than 50% of QC samples or 80% of biological samples were removed, and the remaining peaks with missing values were imputed with the k-nearest neighbor algorithm to further improve the data quality. PCA was performed for outlier detection and batch effect evaluation using the preprocessed dataset. Quality control-based robust LOESS signal correction was fitted to the QC data with respect to the order of injection to minimize signal intensity drift over time. In addition, the relative standard deviations of the metabolic features were calculated across all QC samples, and those > 30% were then removed [37].

Student’s t tests were conducted to detect differences in metabolite concentrations between the 2 phenotypes. The P value was adjusted for multiple tests using an FDR (Benjamini–Hochberg). Supervised PLS-DA was conducted through metaX to discriminate the different variables between groups. The VIP value was calculated. A VIP cutoff value of 1.0 was used to select important features.

### Skin healing test

Six healthy dogs aged 2 to 5 years and weighing 6 to 8 kg (provided by the Laboratory Animal Center of Northwest A & F University) were used in the study. All of the dogs were reared, obtained, and housed in accordance with our institute’s laboratory animal requirements. All procedures and the study design were conducted in accordance with the Guide for the Care and Use of Laboratory Animals (Ministry of Science and Technology of China, 2006) and were approved by the Animal Ethical and Welfare Committee of Northwest Agriculture and Forest University. The dogs were kept in cages in a feeding room without purification equipment at a temperature of 18–25 °C, humidity of 40–60%, airflow value of 0.13–0.18 m/s, ventilation rate of 10–20 times per hour, light normal, and noise below 60 dB.

Dogs were given respiratory anesthesia (Isoflurane, Minimum Alveolar Concentration = 1.3%-1.8%), and a skin trephine was used to create 5 round skin notches with a diameter of 2 cm on the back of the dog. The dogs were administered Vectaxib chewable tablets (Orbiepharm, China, 3 mg/kg/d, for 5 days) and Beylide (Bayer, Germany, 2.5 mg/kg/d, for 5 days) beginning on the first postoperative day. On the day of surgery and 5, 10 and 15 days after surgery, the following reagents were injected around 5 skin defects in each dog, divided into 5 groups. Group 1: Supernatant after cell culture in the FBS group medium. Group 2: Supernatant after cultured cells in the PRP group medium. Group 3: Supernatant after cell culture in FBS Group medium + PRP after activation. Group 4: PRP after activation. Group 5: Normal saline.

The healing of the skin defect was observed in 5, 15 and 25 days after surgery, the healing efficiency was determined according to the healing area, and the paraffin sections were prepared and treated with hematoxylin-eosin (HE) staining.

### Statistical analysis

The experimental data were processed by SPSS 26.0 software, and the results are expressed as the mean ± standard deviation. Comparisons between groups were conducted by t test. *P* < 0.05 was considered a significant difference, and P < 0.01 was considered an extremely significant difference. Bar graphs were constructed using GraphPad Prism 9.5 software.

## Results

### Effects of PRP on biological properties of cADMSCs

**Fig. 1:**
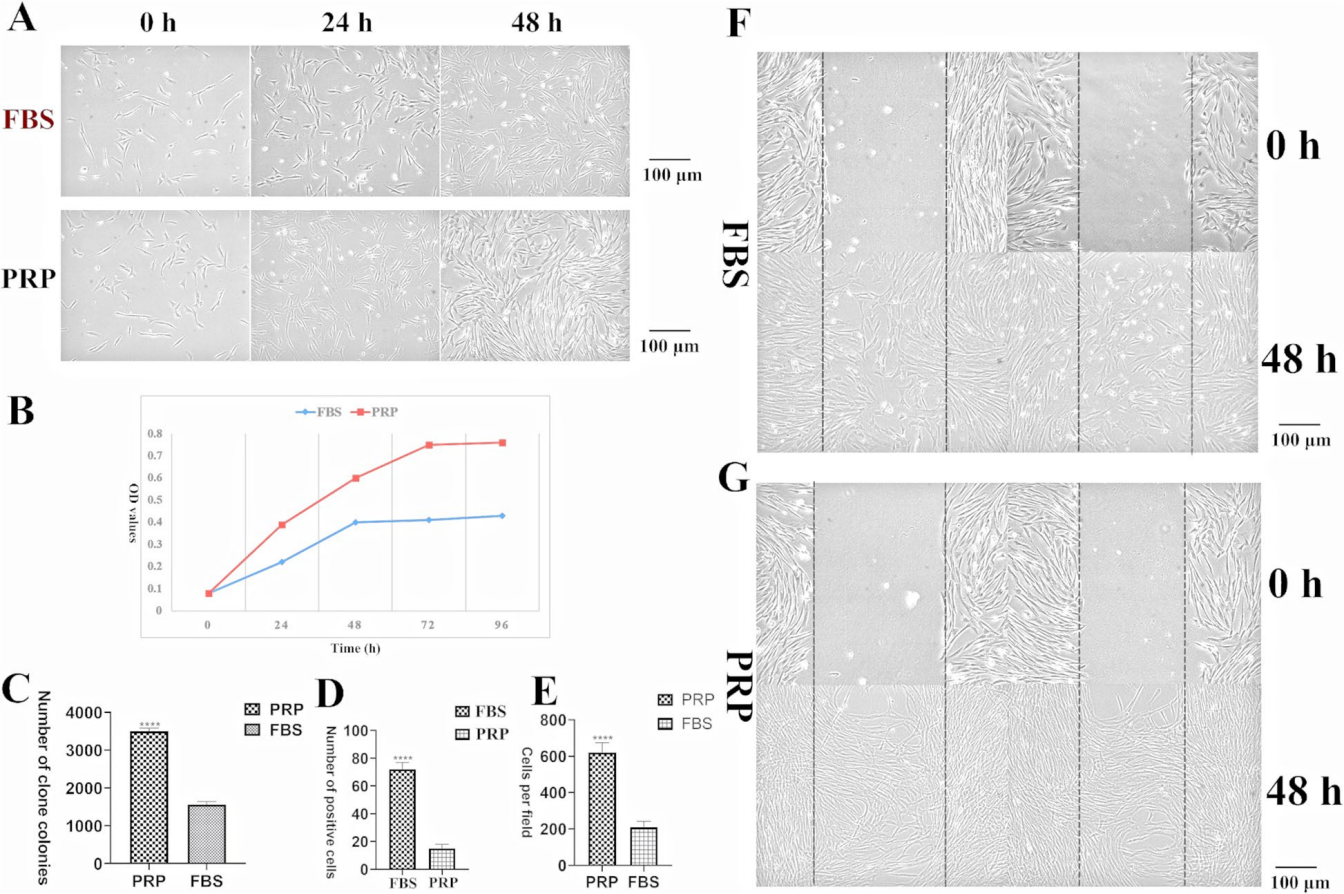
Growth, proliferation and migration of cADMSCs. **A** The growth of the cells was observed at 0, 24, 48 hours. After replacing 10%FBS with PRP, the proliferation capacity of cADMSCs was significantly enhanced, and the cell-to-cell connection was closer. **B** The growth curve of cADMSCs. **C** Compared with FBS, after adding PRP to the cell medium, and the number of cloned cell populations was more, ****: *P*<0.01. **D** The number of senescent cells in the PRP Group was extremely significantly reduced compared with that in the FBS Group, ****: *P*<0.01. **E** In Transwell experiment, after 48 h of cADMSCs pretreated with PRP, the number of migrating cells in the unit area increased significantly, ****: *P*<0.01. **F **and** G** After 48 h of migration, the cells which pretreated with PRP migration rate was significantly higher than that of the untreated group

The platelet content in whole blood of normal dogs was 306 ± 55 × 10^6^ / mL, and the platelet content in PRP was 1638 ± 208 × 10^6^ / mL, and the platelet content in PRP was 5 times higher than that in conventional blood. In subsequent cell cultures, after replacing 10%FBS with PRP (1x10^8^/mL), the proliferation capacity of cADMSCs was significantly enhanced, and the cell-to-cell connection was closer (Fig. 1A and 1B). Compared with FBS, after adding PRP to the cell medium, the cloning-forming ability of the cADMSCs was extremely significantly enhanced, and the number of cloned cell populations increased (Fig. 1C), and the cloning-forming cell population volume was larger. When the senescence of cells was detected, it was found that the number of senescent cells in the PRP Group was extremely significantly reduced compared with that in the FBS Group (Fig. 1D), PRP could prevent the aging of cells. In Transwell experiment (Fig. 1E), after 48 h of cADMSCs pretreated with PRP, and after 48 h of migration, the migration rate of the cells, pretreated with PRP, was significantly higher than that of the untreated group. In cell scratch experiments (Fig. 1F and 1G), the addition of PRP to cell media promoted the migration of cADMSCs. Replacing FBS with PRP in cell culture systems can improve the biological properties of cells, promote the proliferation, migration and cloning of cADMSCs, and slow down the aging process.

### Effect of PRP on the transcriptome of cADMSCs

After replacing 10%FBS with PRP (1x10^8^/mL), PRP can promote the proliferation, migration and clonal formation of cADMSCs, and slow down the aging process. However, how this is achieved, or what changes in gene expression occur in cells during these processes, is unknown. Therefore, this study conducted transcriptome sequencing analysis on cADMSCs cultured in the media of the PRP Group. cADMSCs cultured in CONT Group and FBS Group were taken as control. The raw sequence data have been submitted to the NCBI Short Read Archive (SRA) with PRJNA1002236.

**Fig. 2:**
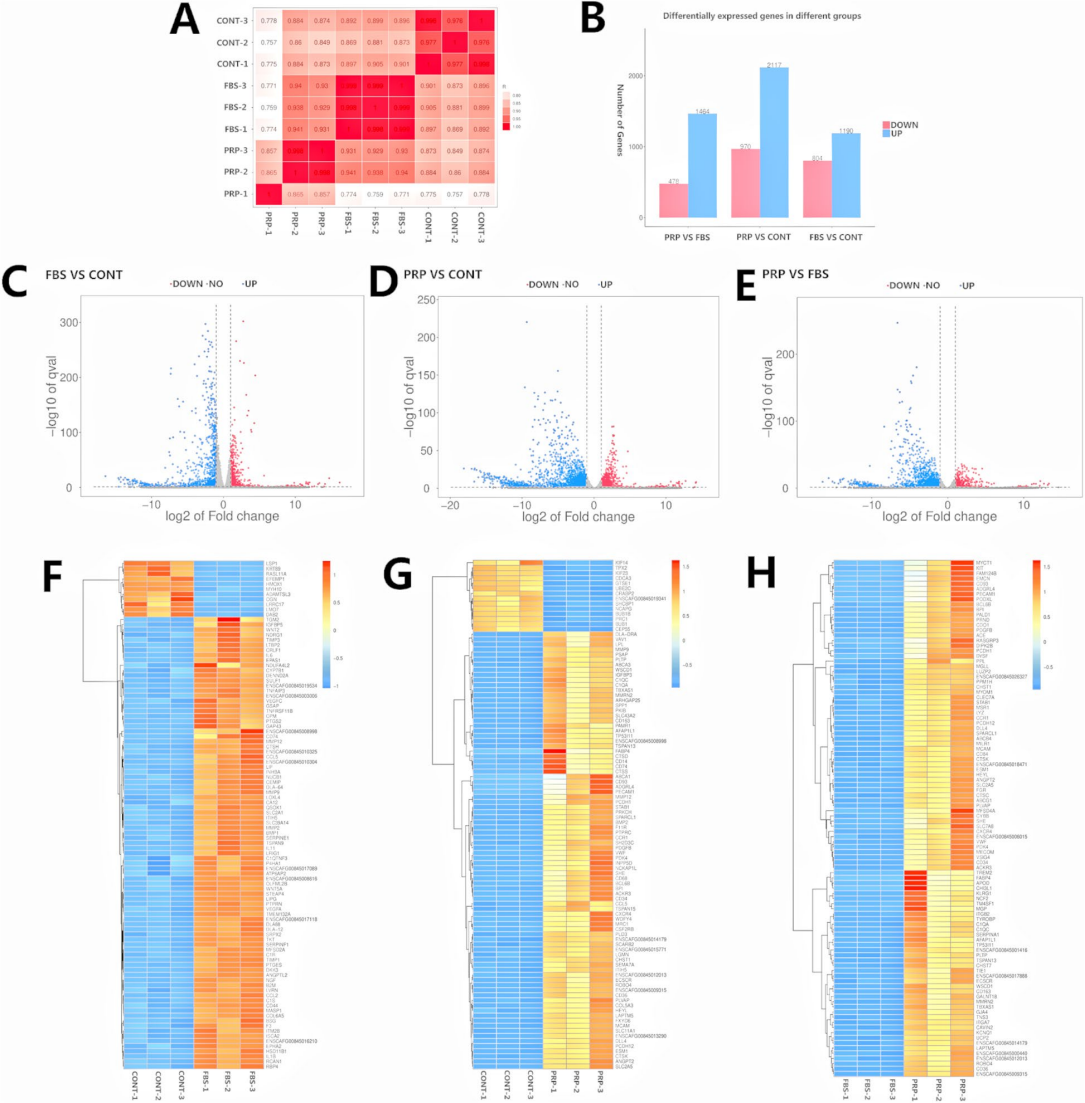
The transcriptome of cADMSCs. **A** Pearson correlation coefficient chart. The horizontal coordinate and vertical coordinate are the sample names respectively, and the color depth indicates the correlation coefficient of the two samples. The closer it is too red, the greater the correlation; The closer it is to white, the less correlated it is. The three biological repeat correlation coefficients of each group were 1 or close to 1, indicating good repeatability. **B** Statistics of differentially expressed genes. The horizontal coordinate represented pair-to-pair comparison between groups, and the vertical coordinate represented the number of differentially expressed genes. Compared with CONT group, gene expression in PRP group and FBS group was increased, and gene expression in PRP group was higher than that in FBS group. **C-E** Volcano map of differentially expressed genes. The horizontal coordinate is log2 (fc) and the vertical coordinate is -log10 (qvalue), where red represents down-regulated significantly differentially expressed genes, blue represents up-regulated significantly differentially expressed genes, and gray dots represent non-significantly differentially expressed genes. C is the result of comparison between FBS and CONT, D is the result of comparison between PRP and CONT, **E** is the result of comparison between PRP and FBS. **F-H** Clustering heat map of differentially expressed genes. The horizontal coordinate is the sample name, and the vertical coordinate is the selected Top100 differentially expressed gene with the smallest q value. Different colors indicate different relative gene expression levels, and colors ranging from blue through white to red indicate low to high expression levels. F is the result of comparison between FBS and CONT, G is the result of comparison between PRP and CONT, H is the result of comparison between PRP and FBS

First, the correlation analysis of gene expression levels in CONT, FBS and PRP groups was conducted. The Pearson correlation coefficient between samples is shown in Fig. 2A, indicating a good repeatability between samples. Then, the difference multiple FC >= 2 or FC <= 0.5 (the absolute value of log2FC >= 1) and q value < 0.05 (|log2fc| >= 1 & q < 0.05) as a threshold criterion, differentially expressed genes were screened and statistically analyzed. Compared with those in CONT group, gene expression levels in PRP and FBS groups were significantly increased. In FBS vs. CONT, 1190 genes were upregulated, while in PRP vs. CONT, 2117 genes were upregulated. In addition, the PRP group had higher gene expression than the FBS group (1464 genes upregulated) (Fig. 2B-E, Supplementary document 1, 2, 3, 4). Subsequently, cluster analysis was conducted on the differentially expressed genes, which was displayed in the form of a heatmap (Fig. 2F-2H). It can be seen from the above results that replacing FBS with PRP in culture system can improve the gene expression ability of cells. At the same time, according to the transcriptomic sequencing results, 18 differentially expressed genes (9 upregulated and 9 downregulated) were selected from each comparison group, and *Gapdh* was used as the internal reference gene for RT‒qPCR validation (Primers are shown in Supplementary document 5). According to the results (Supplementary document 5), the transcriptional sequencing results in this study were correct.

### GO functional enrichment analysis

**Fig. 3:**
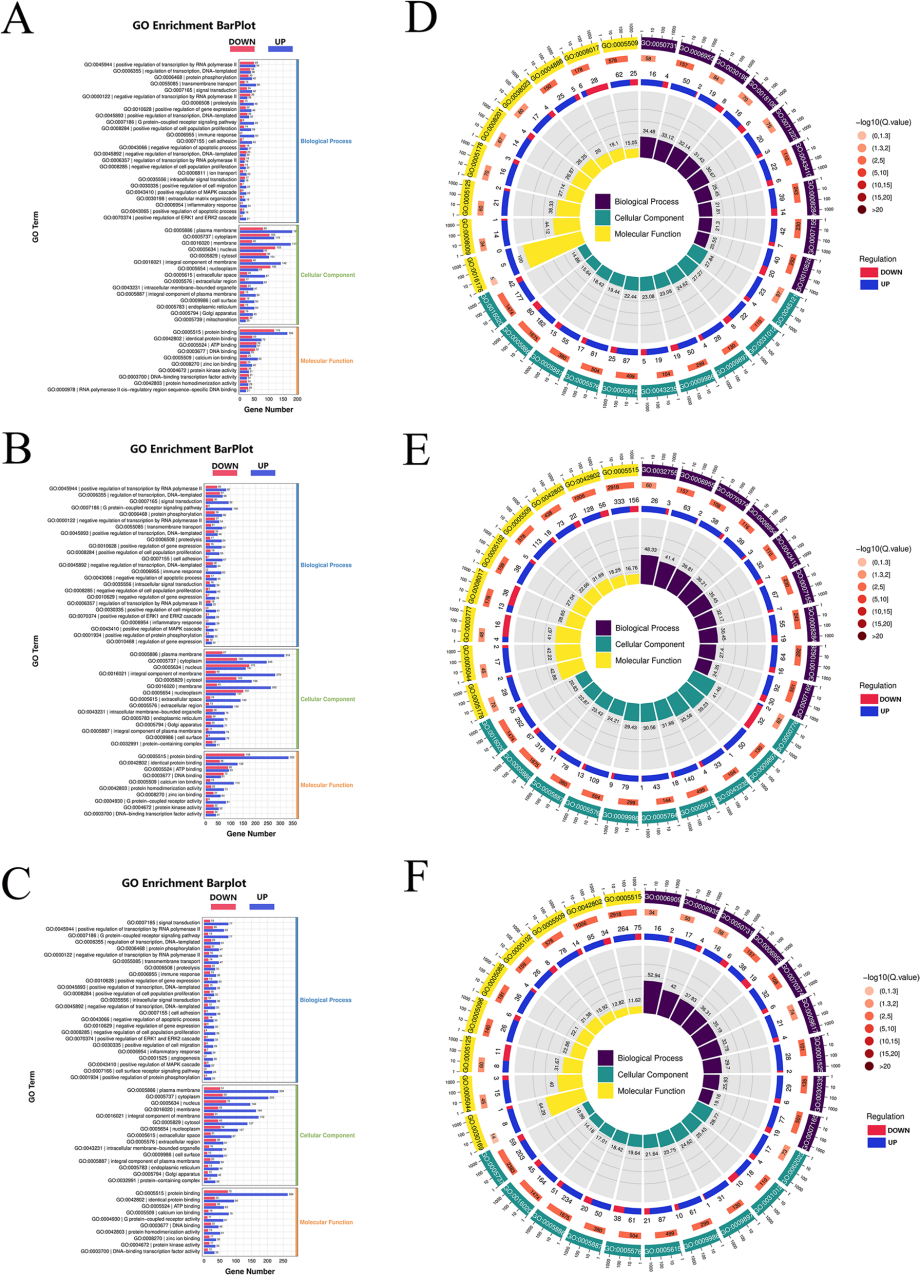
GO functional enrichment analysis. **A-C** GO enrichment up-down bar chart. Regardless of the enrichment significance *P*-value, the number of differentially expressed genes enriched to each GO entry was mainly considered, and the enrichment analysis was carried out from the perspective of the number of differentially expressed genes contained. A is the enrichment result of differentially expressed genes of FBS vs CONT, B is the enrichment result of differentially expressed genes of PRP vs CONT, and C is the enrichment result of differentially expressed genes of PRP vs FBS. **D-F** GO enrichment LoopCircos diagram. The first circle (from outside to inside) is the GO entries enriched Top (minimum P value or minimum Q value), and the outside of the circle is the coordinate scale of the number of genes, different colors indicate the classification of GO three categories; The second circle represents the number of genes annotated to the GO entry (B), and the color represents the -log10 value of the enrichment analysis P value or Q value; The third circle is the statistics of the number of differentially up-regulated and down-regulated genes in GO. The fourth lap represents a Rich Facto percentage. D is the result of differentially expressed gene enrichment of FBS vs CONT, E is the result of differentially expressed gene enrichment of PRP vs CONT, and F is the result of differentially expressed gene enrichment of PRP vs FBS

Subsequently, GO functional enrichment analysis was performed for differentially expressed genes. First, the number of differentially expressed (upregulated or downregulated) genes enriched in each GO item was counted. In FBS vs. CONT, upregulated differentially expressed genes were significantly enriched in positive regulation of transcription by RNA polymerase II, protein phosphorylation, transmembrane transport, positive regulation of gene expression, positive regulation of cell population proliferation, cell adhesion, positive regulation of cell migration, positive regulation of MAPK cascade, positive regulation of ERK1 and ERK2 cascade in Biological Process (Fig. 3A). At the same time, Supplementary document 6 show the enrichment of upregulated differentially expressed genes in Cellular Component and Molecular Function.

In PRP vs. CONT, upregulated differentially expressed genes were significantly enriched in positive regulation of transcription by RNA polymerase II, G protein-coupled receptor signaling pathway, protein phosphorylation, transmembrane transport, positive regulation of gene expression, positive regulation of cell population proliferation, cell adhesion, positive regulation of cell migration, positive regulation of ERK1 and ERK2 cascade, positive regulation of MAPK cascade in Biological Process (Fig. 3B). Supplementary document 7 show the enrichment of upregulated differentially expressed genes in Cellular Component and Molecular Function.

In PRP vs. FBS, upregulated differentially expressed genes were significantly enriched in signal transduction, positive regulation of transcription by RNA polymerase II, G protein-coupled receptor signaling pathway, protein phosphorylation, transmembrane transport, positive regulation of gene expression, positive regulation of cell population proliferation, cell adhesion, negative regulation of apoptotic process, positive regulation of ERK1 and ERK2 cascade, positive regulation of cell migration, positive regulation of MAPK cascade, cell surface receptor signaling pathway in Biological Process (Fig. 3C). At the same time, Supplementary document 8 show the enrichment of upregulated differentially expressed genes in Cellular Component and Molecular Function.

At the same time, according to the size of P value or Q value, the enrichment GO Term with the smallest P value (or Q value) was statistically analyzed. In FBS vs. CONT, upregulated differentially expressed genes were significantly enriched in positive regulation of gene expression, positive regulation of cell population proliferation, cell adhesion, extracellular matrix organization, positive regulation of peptidyl-tyrosine phosphorylation, cellular response to lipopolysaccharide, peptidyl-tyrosine phosphorylation, positive regulation of MAPK cascade in Biological Process (Fig. 3D). In PRP vs. CONT, upregulated differentially expressed genes were significantly enriched in cell adhesion, signal transduction, positive regulation of cell population proliferation, positive regulation of ERK1 and ERK2 cascade, positive regulation of gene expression, positive regulation of interleukin-6 production, positive regulation of MAPK cascade in Biological Process (Fig. 3E). In PRP vs. FBS, upregulated differentially expressed genes were significantly enriched in signal transduction, positive regulation of ERK1 and ERK2 cascade, phagocytosis, chemotaxis, angiogenesis, positive regulation of peptidyl-tyrosine phosphorylation, positive regulation of cell migration in Biological Process (Fig. 3F).

According to the above results, compared with those in the CONT group, genes related to cell proliferation, adhesion, growth, migration and signaling were significantly upregulated in the FBS group and PRP group. Adding either FBS or PRP in cell culture medium can enhance the biological function of cells and promote the expression of related genes. In the comparison between the FBS group and the PRP group, upregulated differentially expressed genes were significantly enriched in GO term, which is related to cell growth, reproduction and migration, and inhibition of apoptosis and senescence, indicating that PRP can enhance the biological function of cADMSCs and can replace FBS in the culture system. In addition, in PRP vs. FBS, upregulated differentially expressed genes were also significantly enriched in the positive regulation of extracellular matrix constituent secretion, positive regulation of protein secretion, peptide secretion, protein secretion. These results indicate that cADMSCs have stronger exosome secretion function after replacing FBS with PRP in culture system.

## KEGG enrichment analysis

**Fig. 4:**
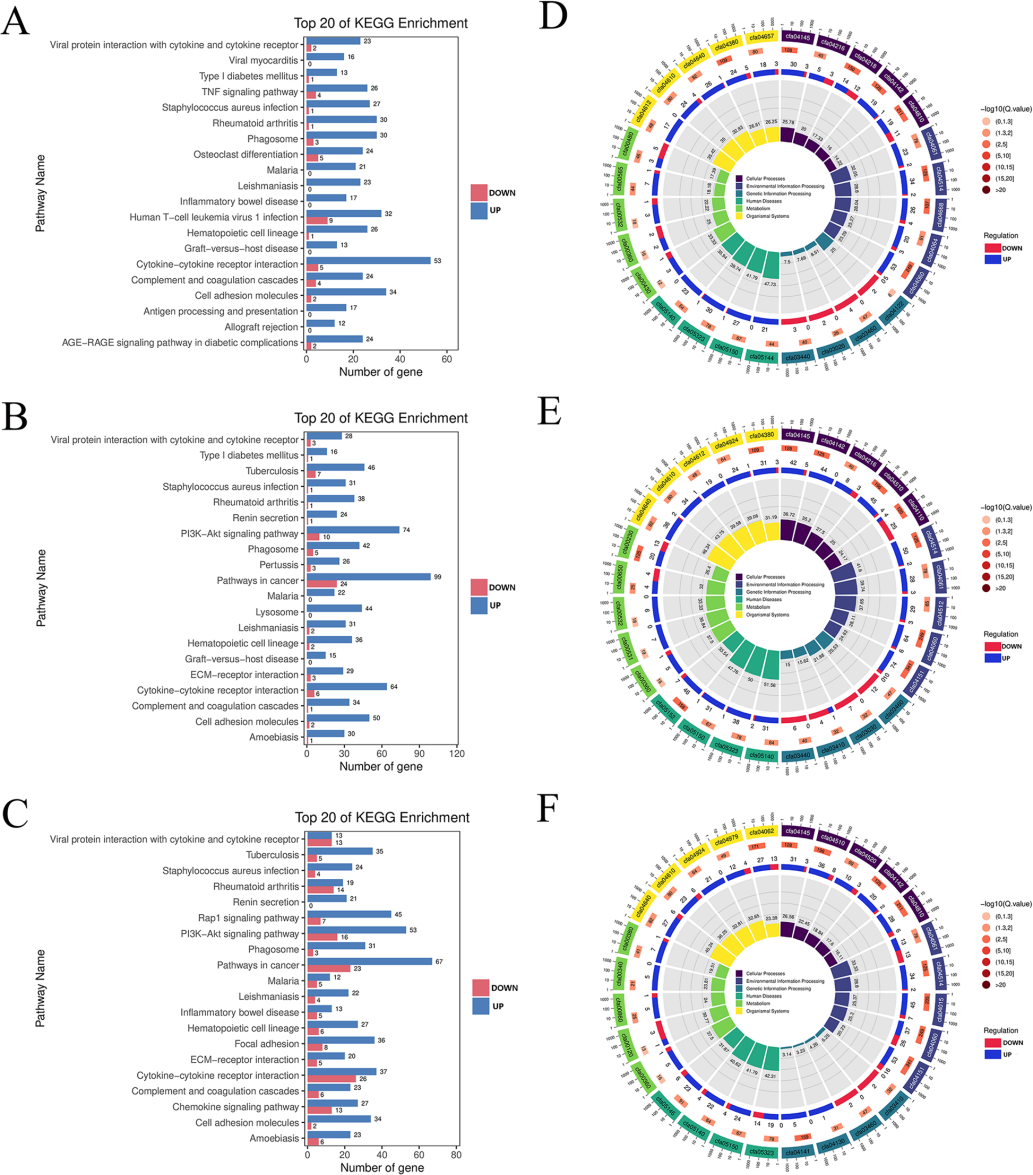
KEGG enrichment analysis. **A-C** KEGG enrichment analysis donut diagram. A is the enrichment result of differentially expressed genes of FBS vs CONT, B is the enrichment result of differentially expressed genes of PRP vs CONT, and C is the enrichment result of differentially expressed genes of PRP vs FBS. **D-F** KEGG enrichment analysis LoopCircos diagram. D is the result of differentially expressed gene enrichment of FBS vs CONT, E is the result of differentially expressed gene enrichment of PRP vs CONT, and F is the result of differentially expressed gene enrichment of PRP vs FBS

At the same time, KEGG enrichment analysis was performed for differentially expressed genes. First, the number of differentially expressed (upregulated or downregulated) genes enriched in each KEGG pathway was counted. In FBS vs. CONT, upregulated differentially expressed genes were mainly enriched in Cytokine‒cytokine receptor interaction, PI3K-Akt signaling pathway, MAPK signaling pathway, Cell adhesion molecules, Chemokine signaling pathway, Rap1 signaling pathway, TNF signaling pathway, Ras signaling pathway (Fig. 4A, Supplementary document 9). Additionally, the pathways with the smallest P or Q values in KEGG enrichment analysis were screened. In addition to the above pathways, Complement and coagulation cascades, NF-kappa B signaling pathway, L-17 signaling pathway were also included (Fig. 4D, Supplementary document 9).

In PRP vs. CONT, upregulated differentially expressed genes were mainly enriched in PI3K-Akt signaling pathway, Cytokine‒cytokine receptor interaction, Rap1 signaling pathway, MAPK signaling pathway, Cell adhesion molecules, Focal adhesion, Chemokine signaling pathway, Ras signaling pathway (Fig. 4B, Supplementary document 10). In addition, the pathways with the smallest P or Q values in KEGG enrichment analysis were screened. In addition to the above pathways, Complement and coagulation cascades was also included (Fig. 4E, Supplementary document 10).

In PRP vs. FBS, upregulated differentially expressed genes were mainly enriched in PI3K-Akt signaling pathway, Rap1 signaling pathway, MAPK signaling pathway, Cytokine‒cytokine receptor interaction, Focal adhesion, Cell adhesion molecules, Ras signaling pathway, Chemokine signaling pathway, cGMP-PKG signaling pathway, Complement and coagulation cascades, cAMP signaling pathway (Fig. 4C, Supplementary document 11). The pathways with the smallest P or Q values in KEGG enrichment analysis were screened. In addition to the above pathways, PPAR signaling pathway was also included (Fig. 4F, Supplementary document 11).

According to the results of KEGG enrichment analysis, compared with those in the CONT group, the upregulated differentially expressed genes in the FBS and PRP groups were mainly enriched in signaling pathways related to cell metabolism, adhesion, growth, survival and proliferation, indicating that the biological function of cADMSCs can be optimized by adding either FBS or PRP in the culture system. In the comparison between the FBS group and the PRP group, the upregulated differentially expressed genes were significantly enriched in several key signaling pathways, which play an important role in cell growth, proliferation and biological function, demonstrating that cADMSCs have stronger biological activity and function after replacing FBS with PRP in the cell culture system.

## Effect of PRP on cellular exosome secretion

According to transcriptomic analysis, after replacing FBS with PRP in the cell culture system, many of the genes in cells were upregulated, and the enrichment of GO function and KEGG of upregulated genes proved that PRP could further enhance the gene expression and biological function of cADMSCs. Currently, exosomes play an important role in the treatment of related diseases through mesenchymal stem cells, and the strong secretion of exosomes from mesenchymal stem cells has an important influence on the clinical application of cells. In this study, transcriptome sequencing analysis proved that PRP can promote the biological function of cADMSCs, and according to GO functional enrichment analysis, it was preliminarily proved that PRP can make cADMSCs secrete more exosomes. However, which exosomes are significantly increased in secretion remains unclear. Therefore, we studied these exosomes by proteomic sequencing of cADMSCs exosomes.

**Fig. 5:**
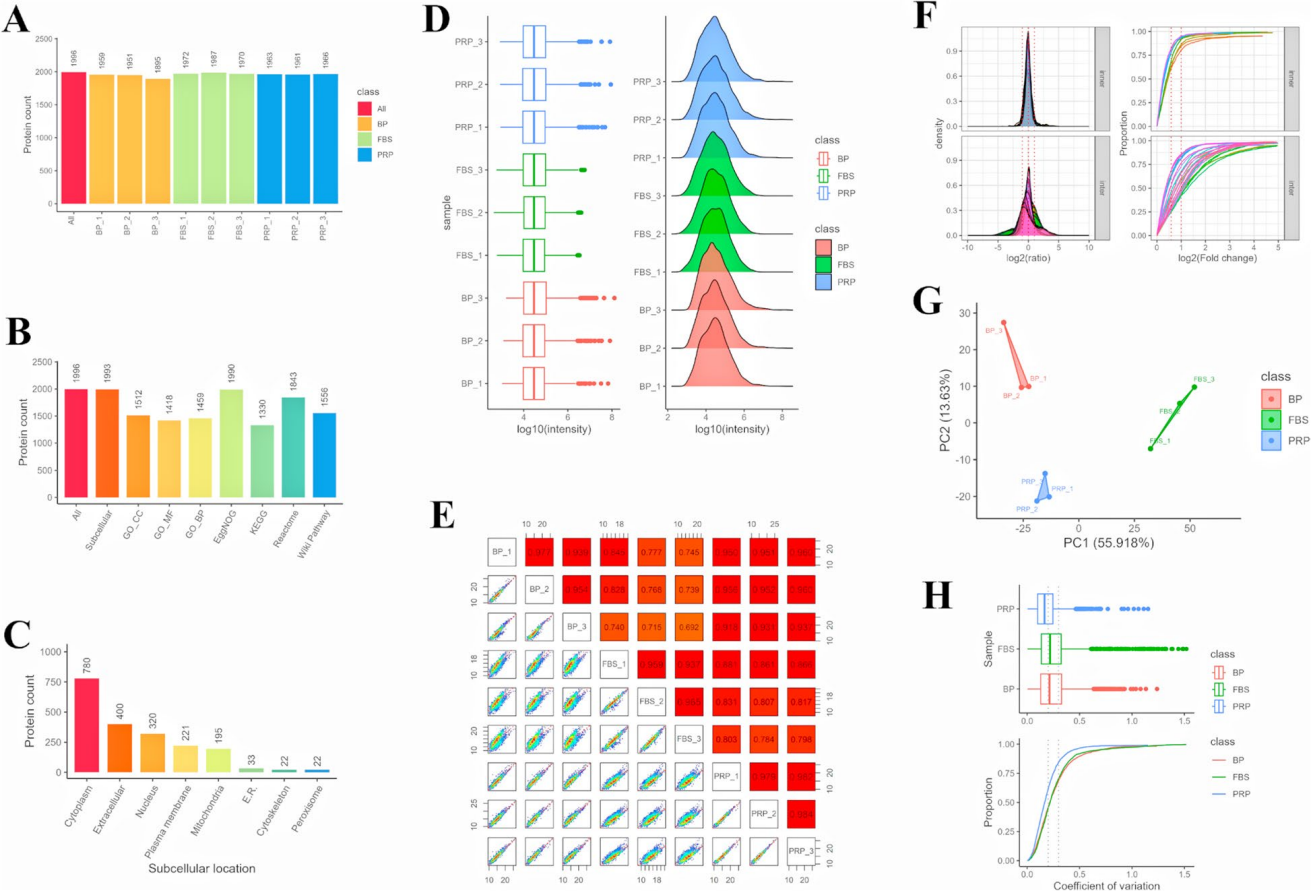
Proteomic sequencing of cADMSCs exosomes. **A** Total protein identification. The total number of proteins identified and the number distribution in each sample, the horizontal coordinate is the group, the vertical coordinate is the protein number. **B** Protein annotation. Functional annotation ratio of proteins in each database. Multiple functional databases (GO, Pathway, Reactome, etc.) were used for functional annotation of the identified proteins to reveal the functional classification of proteins. **C** Subcellular localization. Subcellular localization of identified proteins was performed using WoLF PSORT based on sorting signals, amino acid composition, and function. **D** Protein intensity distribution. The boxplot and density distribution map of the strength of all proteins in each sample can be used to display information such as the median and dynamic range of protein strength, and also understand the differences in distribution between samples. **E** Correlation analysis. The strength of all proteins was log10 as the observed quantity, and the correlation coefficient (Pearson) between samples was calculated with the sample as the variable. The higher the correlation coefficient, the less the difference between samples. F: Ratio distribution between samples. The protein strength was compared in pairwise samples, and the ratio log2 was symmetrical (log2 value > 0, representative ratio > 1). It is assumed that the overall phenotype of the organism does not change much, and the overall protein ratio between the two samples is normally distributed, and most of the ratio is 1 (log2 value is 0). Pair-to-pair ratio distribution was divided into phenotypic groups (inner and inter) to evaluate the differences between samples. G and H: Principal Component Analysis (PCA) and Repeatability analysis

First, exosomes were isolated and extracted from the PRP group (supernatant of cells cultured in PRP Group), FBS group (supernatant of cells cultured in FBS Group) and BP group (10% activated PRP) by ultrafast centrifugation method. Subsequently, the obtained exosome samples were detected by TMT labeled quantitative proteome technology to explore the protein composition, expression differences and corresponding biological functions in the samples. The proteins in the samples were extracted and enzymolized. The enzymolized peptide segments were labeled and enriched with TMT reagent, and the peptides were detected by high-performance liquid chromatography (HPLC) in tandem with high-resolution mass spectrometer to generate a large amount of mass spectrometry data. Detailed information of all identified credible proteins is shown in Supplementary document 12. MaxQuant (v2.1.4.0) software was used to identify proteins in the samples. The identification conditions were: map false-positive (PSM FDR) < 0.01, Protein false-positive (Protein FDR) < 0.01. The total number of proteins identified and their distribution in each sample are shown in Fig. 5A. In the 3 groups, CD63, CD9 and TSG101 were highly expressed. Quality control (QC) information in peptide identification process, including peptide fdr distribution, spectral number of peptide number distribution, protein identification coverage distribution, etc., can be found in Supplementary document 13. The mass spectrometry proteomics data have been deposited to the ProteomeXchange Consortium (http://proteomecentral.proteomexchange.org) via the iProX partner repository with the dataset identifier PXD045208. Then, several functional databases (GO, Pathway, Reactome, etc.) are used to annotate the identified proteins to reveal their functional classification. Figure 5B shows the proportion of functional annotations of proteins in each database, and subcellular localization is shown in Fig. 5C, in which 400 proteins are localized in the Extracellular.

To understand the protein distribution, sample relationship and other information, all the protein strength information quantified by sample identification was evaluated as a whole, including protein strength distribution, relationship between samples, repeatability analysis, etc. The strength of all proteins in each sample was displayed by boxplot and density distribution diagram (Fig. 5D), and information such as median, dynamic range and distribution difference between samples was determined. Meanwhile, log10 was taken as the observed quantity for all protein strengths, and the correlation coefficient between samples (Pearson) was calculated with the sample as the variable to determine the differences between samples (Fig. 5E). In addition, the protein strength was compared in pairwise samples, and the ratio log2 showed a symmetrical distribution. It can be seen that the global protein ratio between the two samples is normally distributed, and the ratio is mostly 1, the difference between samples was shown to be small (Fig. 5F). Principal component analysis and repeatability analysis are shown in Fig. 5G and 5H, which proved that the sample repeatability was good.

**Fig. 6:**
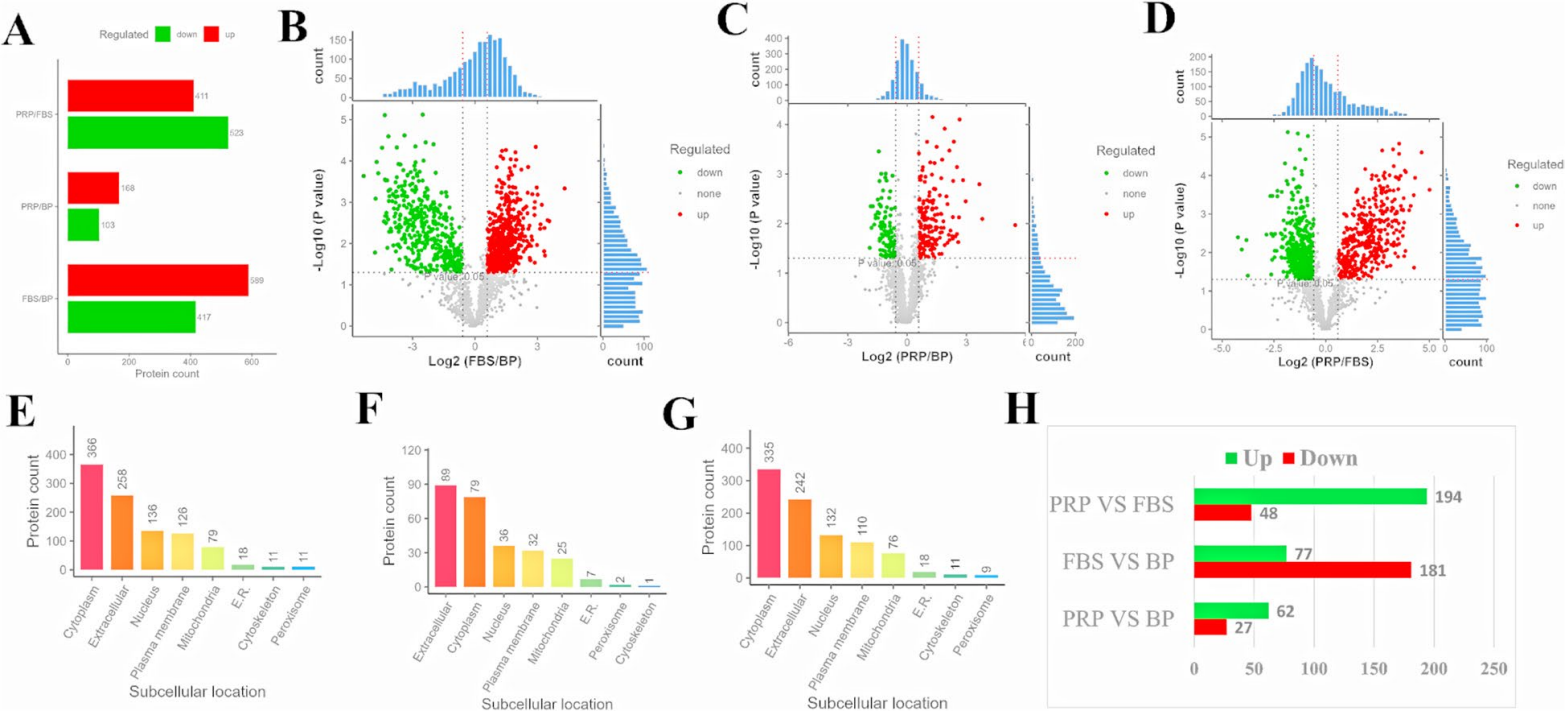
Differential protein analysis. **A** Indifference statistics. Differential proteins were screened by Fold change (FC) greater than 1.5 times and T-test statistical significance (pvalue) less than 0.05. The horizontal coordinate is the number of proteins, and the vertical coordinate is the pairwise comparison group. **B-D** Differentially expressed volcano map. The horizontal coordinate of the volcano map is log2 for the ratio of the comparison group, so that its ratio is symmetrically distributed, that is, log2 (ratio) greater than 0 is a highly expressed protein, and log2 (ratio) less than 0 is a low-expressed protein. B is the result of FSB vs BP, C is the result of PRP vs BP, and D is the result of PRP vs FBS. **E-G** Subcellular localization. Subcellular localization of differential proteins was performed using WoLF PSORT based on sorting signals, amino acid composition, and function. E is the result of FSB vs BP, F is the result of PRP vs BP, and G is the result of PRP vs FBS. **H** The number of differential proteins with Extracellular localization. The horizontal coordinate is the number of proteins, and the vertical coordinate is the pairwise comparison group

Subsequently, the differential protein was screened using the Fold change (FC) greater than 1.5 times, and the significance (*p*value) of T test statistics was less than 0.05 (Fig. 6A, Supplementary documents 14, 15, 16). The volcano diagram of the differential protein is shown in Fig. 6B-6D. In PRP vs. FBS, 411 proteins were upregulated, and in PRP vs. BP, 168 proteins were upregulated. The subcellular localization of the differential proteins was analyzed (Fig. 6E-6G), and the number of differential proteins with Extracellular localization was also analyzed. In PRP vs. FBS, 194 proteins were upregulated, 48 proteins were downregulated, and 62 proteins were upregulated, 27 proteins were downregulated in PRP vs. BP (Fig. 6H, Supplementary documents 17, 18, 19). In PRP vs. FBS, the proteins (subcellular localization is Extracellular) with significantly increased protein expression mainly include: Fibronectin, Peroxidasin, Peptidoglycan-recognition protein, Vitronectin, Kallikrein B1, Extracellular matrix protein 1, Coagulation factor V, Laminin subunit gamma 1, Collagen type VI alpha 3 chain, Coagulation factor XII, Insulin like growth factor binding protein acid labile subunit, Versican, Sushi von Willebrand factor type A EGF and pentraxin domain containing 1. These differential proteins may play an important role in tissue repair. Combined with transcriptome sequencing analysis, it was found that the expression levels of genes corresponding to the significantly increased differential proteins increased. *FN1*, *PXDN*, *PGLYRP1*, *VTN*, *KLKB1*, *ECM1*, *F5*, *LAMC1*, *F12*, *IGFALS* and *VCAN* were increased, but there was no significant difference. The expression levels of *COL6A3* and *SVEP1* were significantly increased. Meanwhile, according to the GO function analysis of proteins, it was found that GO function was extracellular exosome, but subcellular localization is Cytoplasm, Nucleus, Peroxisome, Mitochondria, 66 proteins are upregulated, the main proteins are: Pentraxin family member, S100 calcium binding protein P, Ectonucleotide pyrophosphatase/phosphodiesterase 4, Serpin family F member 2, Procollagen C-endopeptidase enhancer, Coronin, Bactericidal permeability-increasing protein, FERM domain containing kindlin 3, Carbonic anhydrase, Tyrosine-protein kinase, Adiponectin C1Q and collagen domain containing, Ferritin light chain, Glutamine synthetase, Laminin G domain-containing protein, Catalase, Vacuolar protein sorting-associated protein 29 (Supplementary document 20). These differential proteins may play an important role in tissue repair and immune regulation. Combined with transcriptome sequencing analysis, it was found that the expression levels of genes corresponding to the significantly increased differential proteins increased. *CRP, S100P, ENPP4, SERPINF2, CORO1A, SRC* and *GLUL* were increased, but there was no significant difference. The expression levels of *PCOLCE, BPI, FERMT3, CA2, ADIPOQ, FTL, COL18A1, CAT* and *VPS29* were significantly increased. For VEGF, there was no significant difference in PRP vs. FBS, while it was extremely significantly increased in PRR vs. BP, indicating that VEGF secretion did not change after PRP replaced FBS.

At the same time, to eliminate the possible platelet component influence of the cellular supernatant in the PRP group, several differential proteins were also significantly increased in PRR vs. BP: Peroxidasin, Versican, Laminin subunit gamma 1, Sushi von Willebrand factor type A EGF and pentraxin domain containing 1, Collagen type VI alpha 3 chain, Procollagen C-endopeptidase enhancer, Bactericidal permeability-increasing protein. Peroxidasin, as a participant in histogenesis, is an important signal for endothelial cell survival and growth and can support cell matrix synthesis through sulfilimine crosslink-dependent matrix assembly [38, 39], the network of related genes and proteins is shown in Supplementary document-37, according to transcriptome sequencing analysis, many genes in this network are upregulated (Supplementary document 4). Versican is a chondroitin sulfate proteoglycan found in the extracellular matrix that is important for changes in cell phenotype associated with development and disease, it was identified as a fibroblast proteoglycan and forms large multimolecular complexes with hyaluronan and other components of the provisional matrix during wound healing and inflammation [40]. In GO enrichment analysis, Versican is associated with cell adhesion and cell recognition, and in KEGG enrichment analysis, Versican is associated with Cell adhesion molecules (CAMs) signaling pathway. Laminin is a major noncollagenous component of the basement membrane of cells and is involved in a variety of biological processes, including cell adhesion, differentiation, migration, signaling, neurite growth, and metastasis [41]. In the KEGG enrichment analysis, laminin is related to PI3K-Akt signaling pathway, Focal adhesion, ECM-receptor interaction and other signaling pathways. Sushi von Willebrand factor type A EGF and pentraxin domain containing 1 is an EGF-domain-containing extracellular multidomain cell adhesion protein that plays an important biological role in cell adhesion and/or the immune system, mediates cell‒cell adhesion in an integrin-dependent manner in osteoblasts, and plays a key role in epidermal differentiation [42, 43]. Collagen type VI alpha 3 chain can mediate necessary hemostasis by maintaining the integrity and stability of blood vessel wall [44], which can activate muscle stem cells and is clearly correlated with muscle regeneration [45]. In KEGG enrichment analysis, Collagen type VI alpha 3 chain was correlated with signaling pathways such as PI3K-Akt signaling pathway and ECM-receptor interaction. Procollagen C-endopeptidase enhancer is a specific connective tissue glycoprotein that is likely to regulate procollagen processing *in vivo* [46, 47], Procollagen C-endopeptidase enhancer may be used as a therapeutic candidate to enhance host defenses by increasing neutrophil oxidative bursts [48]. In GO enrichment analysis, Procollagen C-endopeptidase enhancer is associated with positive regulation of peptidase activity and peptidase activator activity, and in KEGG enrichment analysis, it is associated with Vitamin digestion and absorption. Bactericidal permeability-increasing protein can not only kill gram-negative bacteria in broad spectrum but also effectively neutralize bacterial endotoxin, to increase the anti-infection ability of the body [49, 50]. In GO enrichment analysis, Bactericidal permeability-increasing protein is associated with immune response, and in KEGG enrichment analysis, it is associated with NF-kappa B signaling pathway.

In summary, replacing FBS with PRP in the cell culture system can significantly enhance the exosome secretion function of cADMSCs. Especially Peroxidasin, Versican, Laminin subunit gamma 1, Sushi von Willebrand factor type A EGF and pentraxin domain containing 1, Collagen type VI alpha 3 chain, Procollagen C-endopeptidase enhancer, Bactericidal permeability-increasing protein that have an important impact on tissue repair, immune regulation and anti-infection. Replacing FBS with PRP in cell culture systems is of great significance for cADMSCs culture and clinical application.

### Skin injury healing test

Through exosomal proteomics, it was found that the use of PRP instead of FBS could significantly enhance the exosomal secretion function of cADMSCs and significantly increase the secretion of related proteins, which is of great significance for the process of tissue repair. To further verify the repair effect, a skin healing test was conducted in this study.

**Fig. 7:**
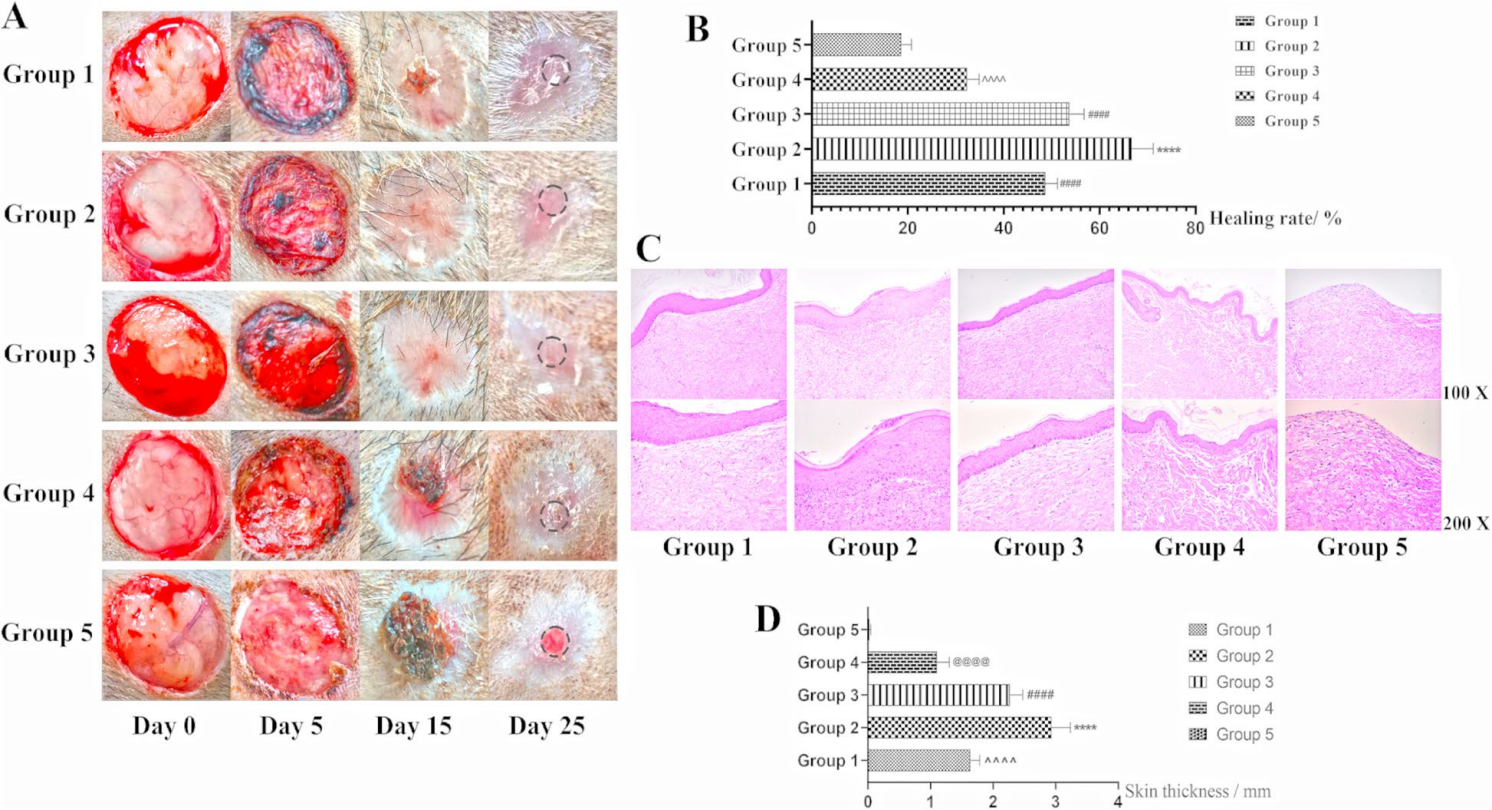
Skin injury healing results. **A** On days 0, 5, 15 and 25, the repair effects of the five groups were observed. **B** The healing rate of skin defects, ****, #### and ^^^^, *P*<0.01. **C**: Tissue section and HE staining were performed at the skin repair site on day 25 (black circle on day 25 in Figure 7A). **D** The skin thickness after repair was statistically analyzed, ****, ####, @@@@ and ^^^^, *P*<0.01

On Days 0, 5, 15 and 25, the repair effects of the five groups were observed, among which Group 2 and Group 3 had the best repair effect, followed by Group 1, and Group 4 and Group 5 had the worst repair effect (Fig. 7A). Subsequently, the healing rate of skin defects (skin defect area - unrepaired area/skin defect area) was statistically analyzed, and the healing rate of skin defects in Group 2 was significantly higher than that in other groups, while there was no significant difference between Group 3 and Group 1, and Group 4 was higher than Group 5 (Fig. 7B). Tissue sectioning and HE staining were performed at the skin repair site on Day 25 (black circle on Day 25 in Fig. 7A). The repair effect of Group 2 was the best, and the dermis and stratum corneum could be observed. The dermis could be observed in all groups except Group 5, but the stratum corneum was very loose (Fig. 7C). Meanwhile, the skin thickness after repair was statistically analyzed. The skin thickness of Group 2 was significantly higher than that of the other groups, followed by Group 3 and Group 1 (Group 3 was significantly higher than Group 1) and Group 4. The skin thickness of Group 5 was very thin. No dermis was formed (Fig. 7D).

### Effect of PRP on cell metabolism

According to the above results, PRP can stimulate the high expression of related genes and signaling pathways to enable rapid cell growth and proliferation. After replacing FBS with PRP in the culture system, cADMSCs have stronger biological activity and biological function, and can secrete more exosomes. However, after replacing FBS with PRP in cell culture system, the specific metabolic pathways of cells are unknown. To explore the changes in the metabolic pathway of cells after PRP treatment, this study conducted nontargeted metabolomics studies on PRP and FBS groups.

**Fig. 8:**
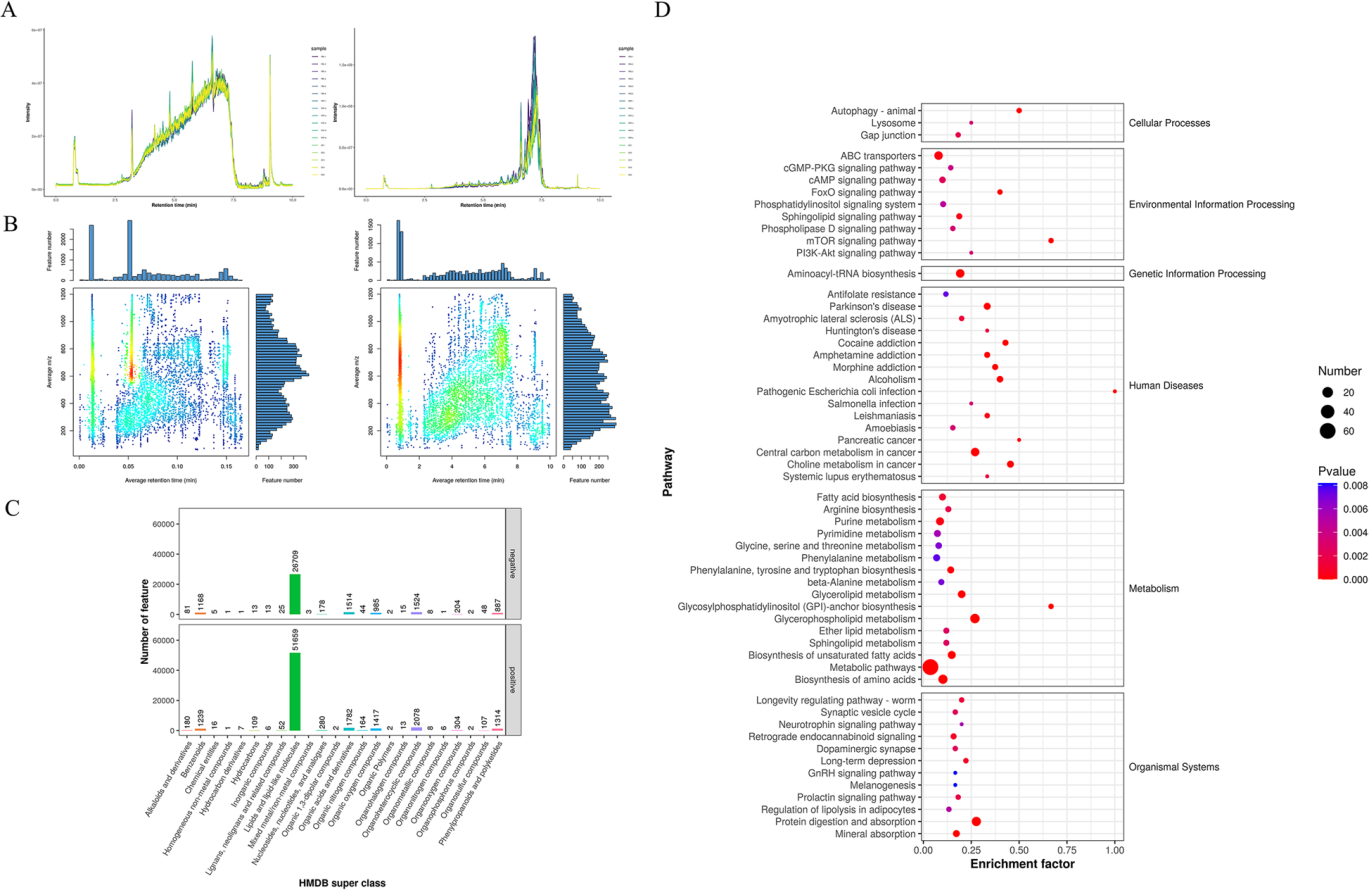
Non-targeted metabolomics. **A** Total Ion Chromatogram. With the time point as the horizontal coordinate, the total strength of all ions in the mass spectrum at each time point is the vertical coordinate, and each color represents a sample. **B** Metabolite m/z-rt distribution map. The retention time is the horizontal coordinate and m/z is the vertical coordinate. Each point represents a substance, and the color indicates how dense the substance is in that area; The darker the color, the larger the number of Feature numbers. **C** HMDB Super class classification diagram. Take the Super class entry as the horizontal coordinate. Corresponding article number of metabolites ordinate. **D** The Pathway entries with the smallest P-values. According to the enriched P-values, the Pathway entries with the smallest *P*-values are screened

First, quality control was carried out, including Total Ion Chromatogram test and m/z-rt distribution of metabolites (Fig. 8A and B), to control the overall mass spectrum signal intensity of the samples and quantify the metabolites more accurately. Subsequently, metaX was used to match the m/z of the substance with KEGG to obtain the identification result of primary metabolites, and the HMDB was matched to obtain the HMDB result (Supplementary document 21). The HMDB Super class classification is shown in Fig. 8C, most of the primary metabolites are lipids and lipid-like molecules. The metabolites were matched with the KEGG database, and the identification results were shown in Supplementary document 22.

Then, the different substances are broken in the mass spectrometer, and the second-level atlas is generated using the fragment information. It was matched with the secondary spectrum of standard substances in the public database, and the matching results were scored, and the secondary identification results of metabolites were obtained (Supplementary document 23). The pathways involved in the obtained secondary metabolites are shown in Supplementary document 24. Meanwhile, according to the enriched P values, the pathway entries with the smallest P values are screened (Fig. 8D). XCMS software is used to extract the signal intensity information of each substance in different samples and metaX software is used to carry out quality control. First, low quality peaks are removed, and then KNN (K-Nearest Neighbors) is used to fill in missing values. Then, PQN (Probabilistic Quotient Normalization) and QC-RSC (QC-robust spline batch correction) were used for data normalization (Supplementary document 25). The correlation analysis between metabolites and samples was conducted through the normalized data of metabolites detected in positive and negative ion modes (Supplementary document 26). Metabolomics data have been deposited into the CNGB Sequence Archive (CNSA) [51] of China National GeneBank DataBase (CNGBdb) [52] with accession number CNP0005577 (https://db.cngb.org/search/?q=CNP0005577).

**Fig. 9:**
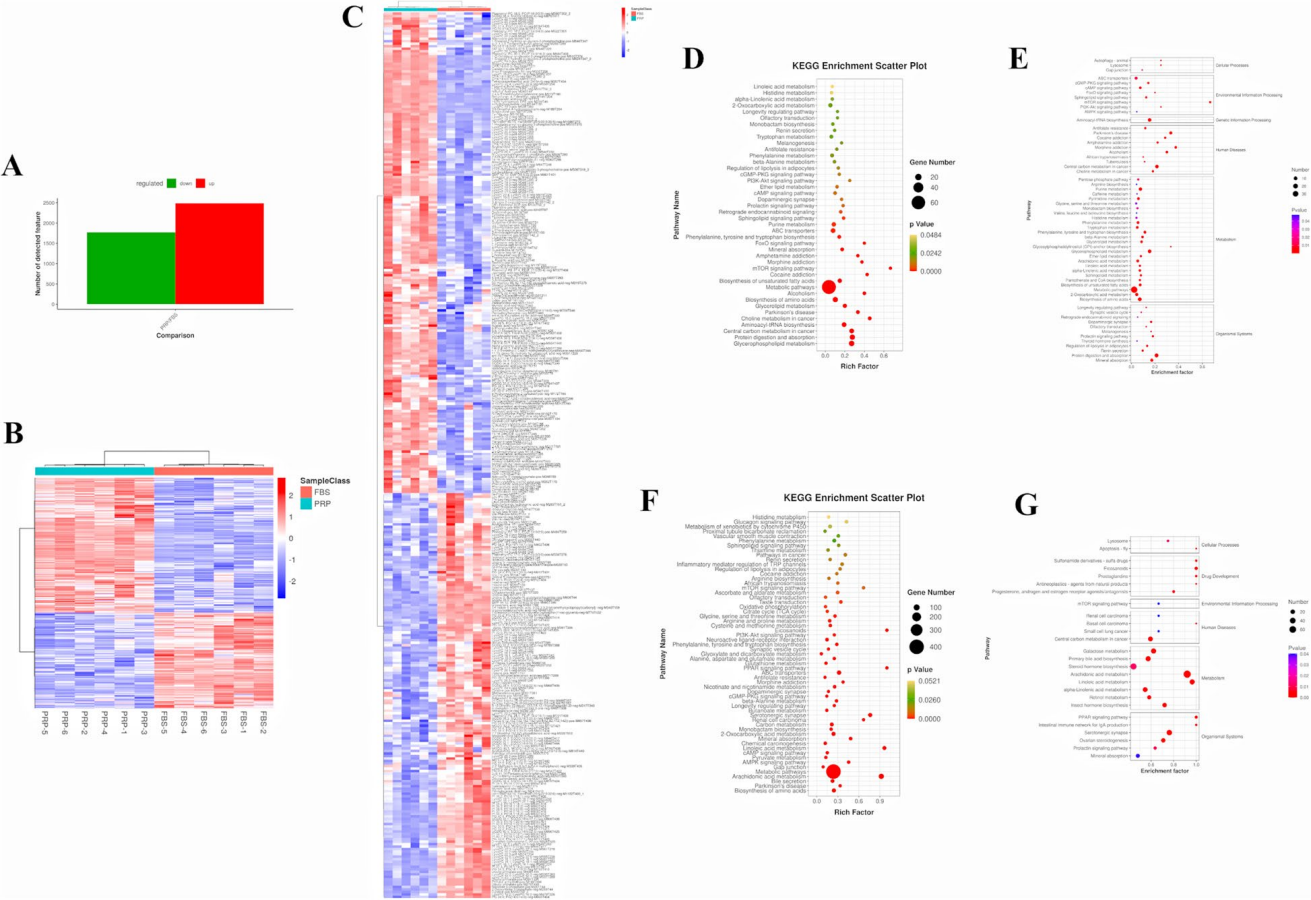
Differential metabolite analysis. **A** Statistical histogram of differential metabolic ions. **B** and **C** Comparison group differential metabolite heat maps. **D**: The KEGG pathway enriched by differential metabolites (Secondary differential (upregulated) metabolites). **E** According to the size of the *P*-value, the KEGG pathway with the lowest *P*-value was screened (Secondary differential (upregulated) metabolites). **F** The KEGG pathway enriched by differential metabolites (primary difference (up-regulated) metabolites). **G** According to the size of the *P*-value, the KEGG pathway with the lowest P-value was screened (primary difference (up-regulated) metabolites)

Then, univariate analysis of the difference multiple and the T-test were used to obtain q-value by BH correction. Combined with multivariate statistical analysis of PLS-DA, VIP (Variable Important for the Projection) value was obtained. Metabolic ions with differential expression were screened (Fig. 9A), and the total table of differential substances was shown in Supplementary documents 27, 28. After the intensity values of each metabolite were normalized, heatmaps were used to show the expression of differential substances in different samples (Fig. 9B and 10C). After replacing FBS with PRP in culture system, the metabolites in cADMSCs changed drastically, many metabolites were upregulated and downregulated, and the cADMSCs produced metabolic reprogramming. PCA and PLS-DA analysis are shown in Supplementary documents 29, 30, 31. All differential-metabolite-enriched KEGG pathways are shown in Supplementary documents 32, 33.

The main secondary differential (upregulated) metabolites are: Guanine, Histidine, Indole-3-acetaldehyde, L-Phenylalanine, Tryptophan, Tyrosine, L-Proline, Indole, Pyroglutamic acid, L-Tyrosine, L-Aspartic acid, Sphingomyelin, Adenosine, AMP, cAMP, etc. Figure 9D shows the KEGG pathway enriched by these differentially abundant metabolites. At the same time, according to the size of the P value, the KEGG pathway with the lowest P value was screened (Fig. 9E). The main primary difference (upregulated) metabolites are: HETE, LysoPC, Tyr-His, Alpha-Linolenic acid, LysoPG, Acylcarnitine Guanine, Histidine, Indole-3-acetaldehyde, L-Phenylalanine, Goshuyic acid, Tryptophan, Tyrosine, L-Proline, Pyroglutamic acid, Phenaceturic acid, Tyrosine, Sphingosine, S1P, Adenosine, AMP, cAMP, etc. Figure 9F shows the KEGG pathway enriched by these differentially abundant metabolites. At the same time, according to the size of the P value, the KEGG pathway with the lowest P value was screened (Fig. 9G).

The main enrichment pathways of differentially abundant metabolites in Environmental information processing were ABC transporters, cGMP-PKG signaling pathway, and Sphingolipid signaling pathway, cAMP signaling pathway, mTOR signaling pathway, PI3-Akt signaling pathway. In transcriptome sequencing, upregulated genes are also significantly enriched in the above metabolic pathways (Supplementary document 11). These results indicate that after replacing FBS with PRP in cell culture systems, cells can regulate cell growth, proliferation, migration and various biological functions through these metabolic pathways. After PRP treatment of cADMSCs, substances such as adenosine released by platelet dense particles can activate related genes through G protein-coupled receptors, and metabolites such as SM and SIP in cADMSCs are significantly increased to activate the Sphingolipid signaling pathway, thereby regulating cADMSCs growth, proliferation, migration, and secretion (Supplementary document 34). At the same time, adenosine, dopamine and other substances released by platelet dense particles can activate cAMP signaling pathway through G protein-coupled receptors and adenylate cyclase pathway and regulate cell state and various biological functions through PKA (Supplementary document 35). In transcriptome GO functional enrichment analysis, upregulated differentially expressed genes were significantly enriched in the G protein-coupled receptor signaling pathway, while in the adenylate cyclase pathway, ATP was derived from platelet dense particles. ATP may also be related to the mitochondrial transfer of platelets and the enhancement of MSCs respiration [12].

In Metabolism, differential (upregulated) metabolite enrichment pathways are mainly: Biosynthesis of amino acids, Aminoacyl-tRNA biosynthesis, Thiamine metabolism, Phenylalanine, tyrosine and tryptophan biosynthesis, Phenylalanine metabolism, Nicotinate and nicotinamide metabolism, Linoleic acid metabolism, Histidine metabolism, Glyoxylate and dicarboxylate metabolism, Glycine, serine and threonine metabolism, Cysteine and methionine metabolism, beta-Alanine metabolism, Arginine biosynthesis, Arginine and proline metabolism, Arachidonic acid metabolism, Alanine, aspartate and glutamate metabolism, 2-Oxocarboxylic acid metabolism, etc. (Supplementary document 36). In transcriptome analysis, many upregulated differentially expressed genes were also enriched in GO function and KEGG pathway related to gene expression, protein phosphorylation, protein synthesis, and protein transport (Supplementary document 8 and Supplementary document 11). The results show that PRP can cause metabolic reprogramming of cADMSCs, especially significant changes in amino acid metabolism, which has an important impact on the exosome secretion function of cells.

Combined with exosomal proteomics and metabonomics, it can be seen that PRP can cause metabolic reprogramming of cADMSCs, strengthen the metabolism of amino acids and fatty acids, and promote the synthesis of various proteins and improve the function of secreting various factors.

## Discussion

MSCs exist in bone marrow, fat, placenta, umbilical cord, dental pulp and periodontal tissues, showing low immunogenicity and multilineage differentiation, with strong self-renewal ability and immunomodulatory effects [53]. In addition, compared with those of embryonic stem cells (ESCs) and induced pluripotent stem cells (iPSCs), MSCs have fewer ethical problems, lower tumorigenic risk, and a wide range of clinical applications. In recent years, a large number of basic research and clinical research results have emerged, but in clinical application, the positive results obtained in animal model studies have not been consistently replicated in clinical trials due to the large number of deaths after MSCs implantation at the injured site [5]. To solve this problem, it is necessary to significantly improve the biological properties of MSCs through alternative methods, improve the therapeutic effect based on MSCs, and achieve better the functional recovery of damaged tissues [54, 55]. PRP contains factors such as PDGF, EGF, FGF, IGF-1, IGF2 and VEGF [56], which can stimulate the migration and proliferation of fibroblasts, endothelial cells and chondrocytes, improve extracellular matrix secretion and angiogenesis, and promote the chemotaxis of macrophages, monocytes and polymorphonuclear cells to regulate inflammation [57]. In addition, activated PRP contains ribosomal RNA, microRNA, non-coding RNA, lipids and extracellular vesicles, etc., which may also be involved in tissue repair and regeneration [58]. Despite positive results in preclinical studies, clinical trials using PRP have led to controversial results, and when PRP is used alone, the release of associated factors is temporary and requires multiple injections [59–62]. Therefore, some studies suggest the use of PRP as an adjuvant to create and improve the microenvironment in which MSCs grow, enhance the biological properties of MSCs, and thus improve the clinical efficacy to achieve the effect of 1+1 > 2 [63, 64].

In this study, replacing FBS with PRP in cell culture systems can improve the biological properties of cADMSCs, promote the proliferation, migration and clonal formation of cADMSCs, and slow down the aging process. Other researchers have also found that PRP can promote the proliferation and migration of MSCs, reduce apoptosis, necrosis and senescence, and improve their survival under oxidative stress [65, 66]. Many researchers have reached a consensus that PRP can promote the proliferation and migration of MSCs and reduce cell apoptosis. This phenomenon should be consistent across species. In this study, we conducted transcriptome sequencing analysis on MSCs stimulated by activated PRP, analyzed the changes in the transcription level of MSCs, and explored the effects of PRP on MSCs from the gene level. The transcriptome sequencing results were verified by RT-qPCR. In the GO functional enrichment analysis, we found that compared with those in the CONT group and FBS group, genes related to cell proliferation, adhesion, growth, migration and signal transduction were significantly upregulated in the PRP group, indicating that PRP can stimulate the high expression of related genes in MSCs, enhance the biological function of MSCs, and can replace FBS in the culture system. In PRP vs. FBS, upregulated differentially expressed genes enriched in positive regulation of extracellular matrix constituent secretion, positive regulation of protein secretion, peptide secretion and protein secretion in GO functional enrichment analysis. This result indicate that cADMSCs may have stronger exosome secretion function after replacing FBS with PRP in the cell culture system, which is consistent with the findings of C. Ji *et al.* [67]. According to the results of KEGG Pathway enrichment analysis, compared with those in the CONT group and FBS group, upregulated differential expression genes in PRP group were mainly enriched in several key signaling pathways related to cell metabolism, adhesion, growth, survival and proliferation, which proved that after replacing FBS with PRP in the cell culture system, cADMSCs have stronger biological functions.

Subsequently, in the sequencing of exosome TMT protein, we found that replacing FBS with PRP in the cell culture system could significantly enhance the exosome secretion function of cells. In PRP vs. FBS, 194 extracellular proteins were upregulated, and 48 extracellular proteins were downregulated. Especially proteins such as, Peroxidasin, Versican, Laminin subunit gamma 1, Sushi von Willebrand factor type A EGF and pentraxin domain containing 1, Collagen type VI alpha 3 chain, Procollagen C-endopeptidase enhancer, Bactericidal permeability-increasing protein, which have an important effect on tissue repair, immune regulation and anti-infection, were significantly increased. However, for VEGF, there was no change in VEGF secretion after replacing fetal bovine serum with PRP, which was inconsistent with the findings of Levoux *et al.*, and the specific reasons still need further research and evidence [12]. However, regardless of which protein or factor, after replacing FBS with PRP, exosome secretion function is certainly enhanced, which is of great significance for cell culture and clinical application. In the follow-up studies, we will conduct more in-depth detection and analysis of some important proteins, such as: which pathway enables MSCs to enhance the expression of these proteins.

By using PRP to culture cADMSCs, we observed that PRP can improve the biological properties of cADMSCs from cell state, cell gene expression, and exosome secretion, and replacing FBS in the vitro culture system is feasible. However, the metabolic changes in this process are still unknown. Levoux *et al.* found that after MSCs are transported to obtain platelet mitochondria, cells can improve the factor secretion function of MSCs by activating new fatty acid synthesis pathways [12]. In addition, the metabolic changes of MSCs still need to be further explored. Therefore, in this study, nontargeted metabolomics analysis was performed on cells cultured with PRP. After replacing FBS with PRP in cell culture systems, PRP can regulate cell growth, proliferation, migration, and various biological functions by ABC transporters, cGMP-PKG signaling pathway, Sphingolipid signaling pathway, cAMP signaling pathway, mTOR signaling pathway and PI3-Akt signaling pathway. This finding is closely associated with adenosine, dopamine and other substances released by platelet dense particles, more in-depth research will be conducted in the future. At the same time, PRP can cause cells to undergo metabolic reprogramming, especially the significant changes in amino acid metabolism and fatty acid metabolism. Amino acid metabolism, on the one hand, is mainly used to synthesize the body's own unique proteins, peptides and other nitrogen-containing substances. On the other hand, through deamination, transamination, combined deamination or decarboxylation, decomposition into α-ketoacids, the conversion of amino acids into sugars, lipids or resynthesis of some nonessential amino acids can occur. Amino acids can also be oxidized into carbon dioxide and water through the tricarboxylic acid cycle, and release energy. Fatty acid metabolism, especially linoleic acid metabolism and arachidonic acid metabolism, can regulate various physiological activities of cADMSCs, and these metabolic pathways have important effects on the secretion of exosomes and other physiological functions of cADMSCs. Similarly, in transcriptome analysis, many upregulated differentially expressed genes were also enriched in GO function and KEGG pathways related to gene expression, protein phosphorylation, protein synthesis, and protein transport.

In this study, no more in-depth joint multi-omics analysis was performed. We will conduct other types of machine learning in the later stage to make it more clear through which components of activated PRP can stimulate the improvement of the biological characteristics of mesenchymal stem cells, and explore which metabolic pathways these components play a role. In summary, by detecting the effects of PRP on the biological performance of cADMSCs, combined with multiomics analysis and related tests, this study analyzed the specific ways, relevant mechanisms and metabolic pathways by which PRP improves the biological performance of cADMSCs. The changes of exosome secretion and therapeutic effect of cADMSCs were revealed after PRP replaced fetal bovine serum. These results provide theoretical and technical references for optimizing the MSCs culture system, improving the biological properties and clinical application effects of MSCs, and achieving better the functional recovery of damaged tissues.

## Conclusion

In the *in vitro* cell culture system, the biological performance of MSCs was significantly improved after using PRP instead of FBS, and the genes related to cell proliferation, adhesion, growth, migration and signal transduction were significantly upregulated. PRP can enhance the secretion function of MSCs exosomes and significantly upregulate many proteins involved in tissue repair, immune regulation and anti-infection. After replacing FBS with PRP, MSCs undergo metabolic reprogramming, which changes the metabolism of amino acids, fatty acids and multiple signaling pathways, thus promoting the synthesis of various proteins, improving the biological performance of caDMSCs and the secretion of various factors (Supplementary document-38).

### Supplementary Information

**Supplementary Material 1.**

## Data Availability

The datasets generated and/or analysed during the current study are available in the NCBI Short Read Archive (SRA) with PRJNA1002236, the ProteomeXchange Consortium (http://proteomecentral.proteomexchange.org) via the iProX partner repository with the dataset identifier PXD045208, and Metabolomics data have been deposited to the EMBL-EBI MetaboLights database (10.1093/nar/gkad1045, PMID:37971328) with the identifier MTBLS9602 (https://www.ebi.ac.uk/metabolights/MTBLS9602).
