# Supplementary material for: Multiomics analysis of platelet-rich plasma promoting biological performance of mesenchymal stem cells
Source: BMC Genomics. 2024 Jun 5;25:564. doi: 10.1186/s12864-024-10329-8 (PMC11151483; doi:10.1186/s12864-024-10329-8)

## Slide 1
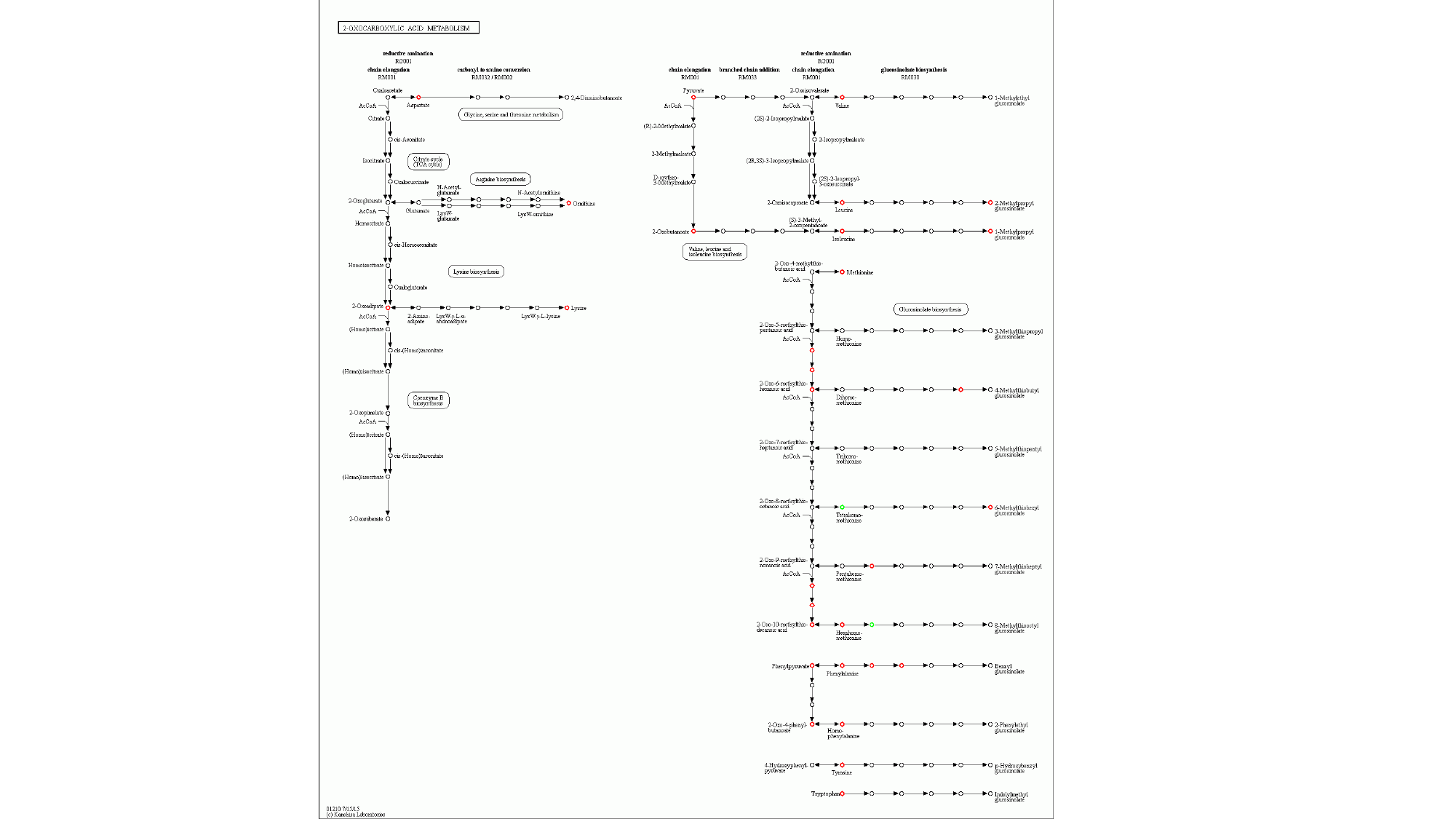

## Slide 2
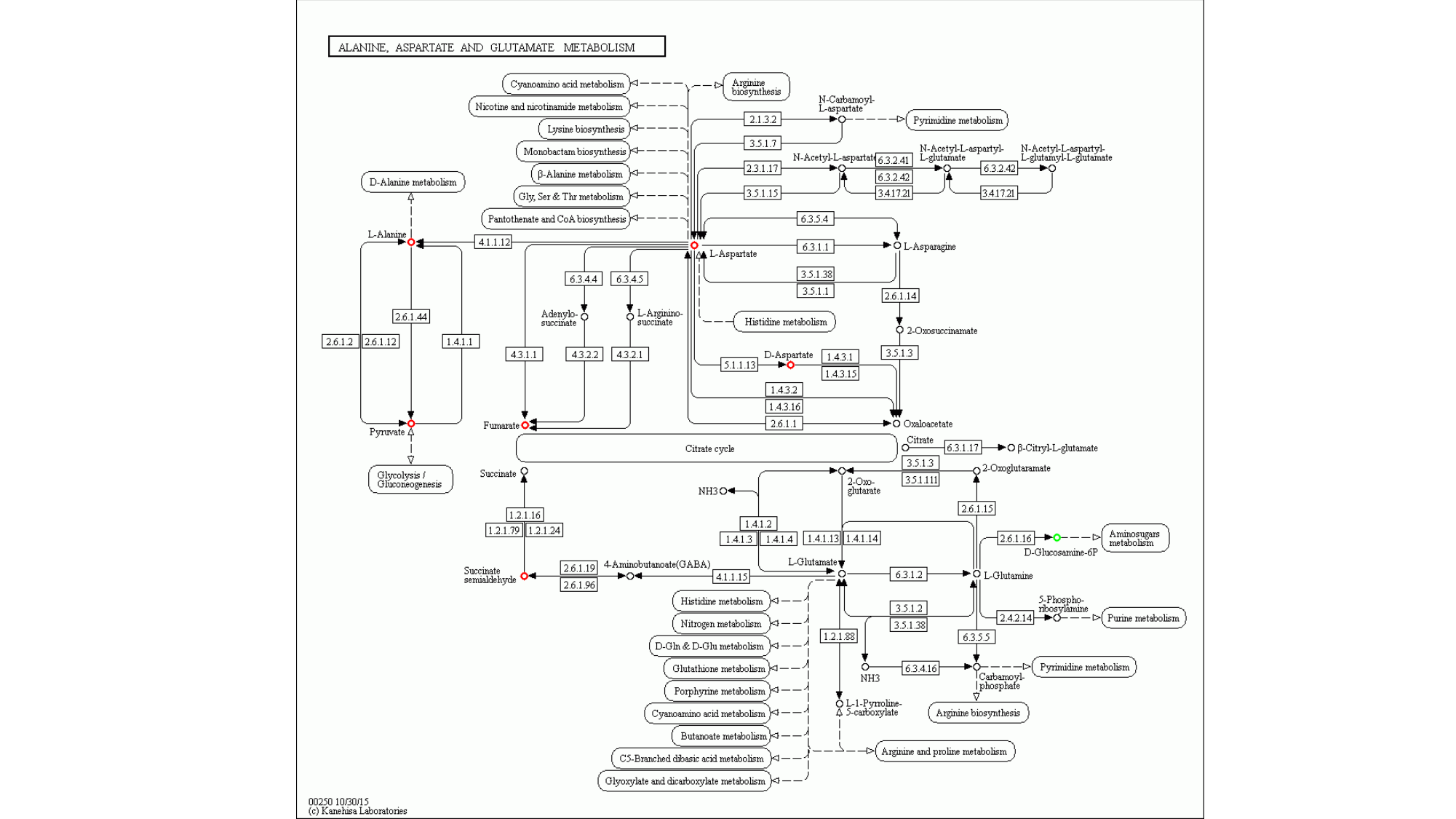

## Slide 3
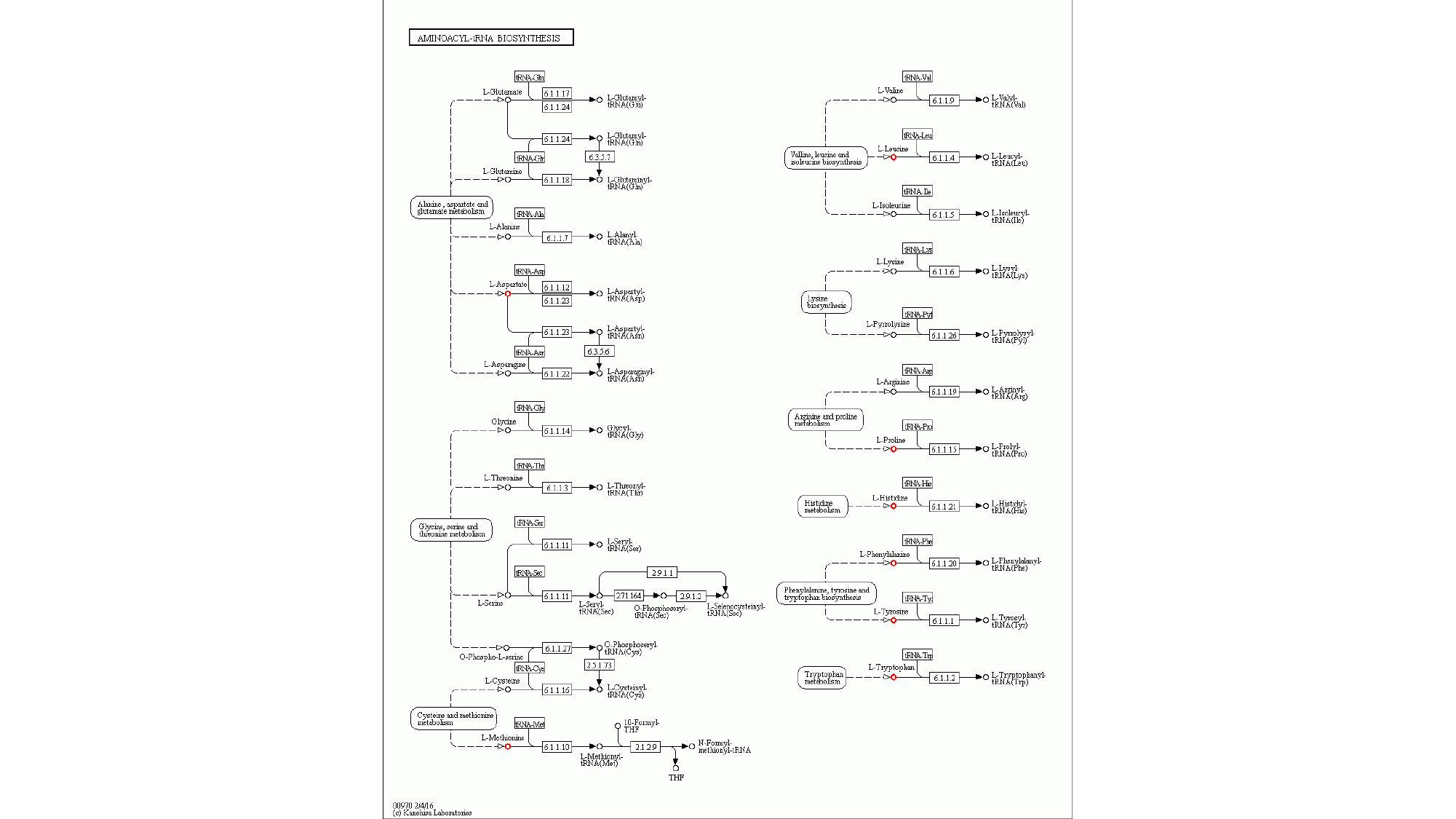

## Slide 4
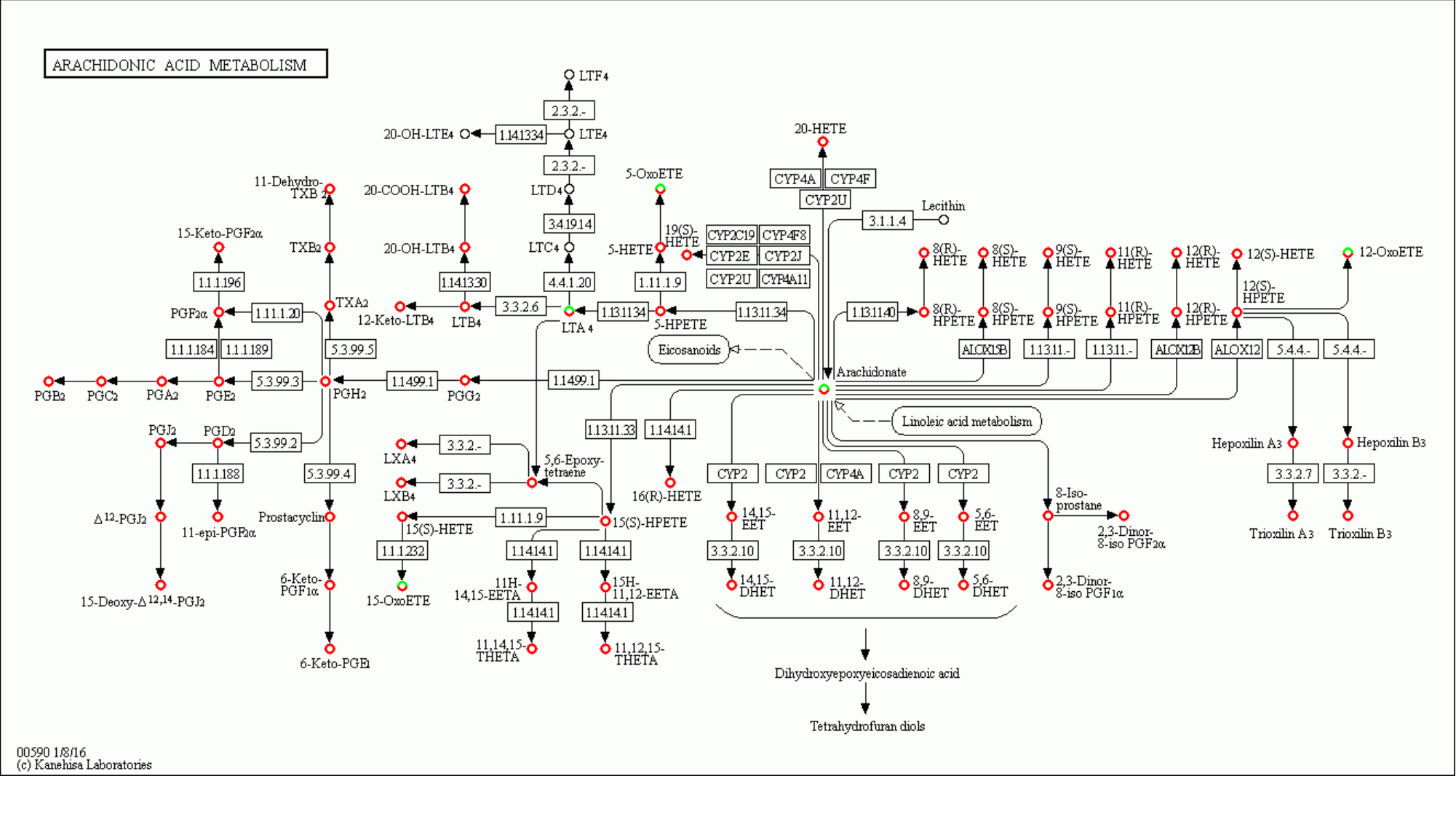

## Slide 5
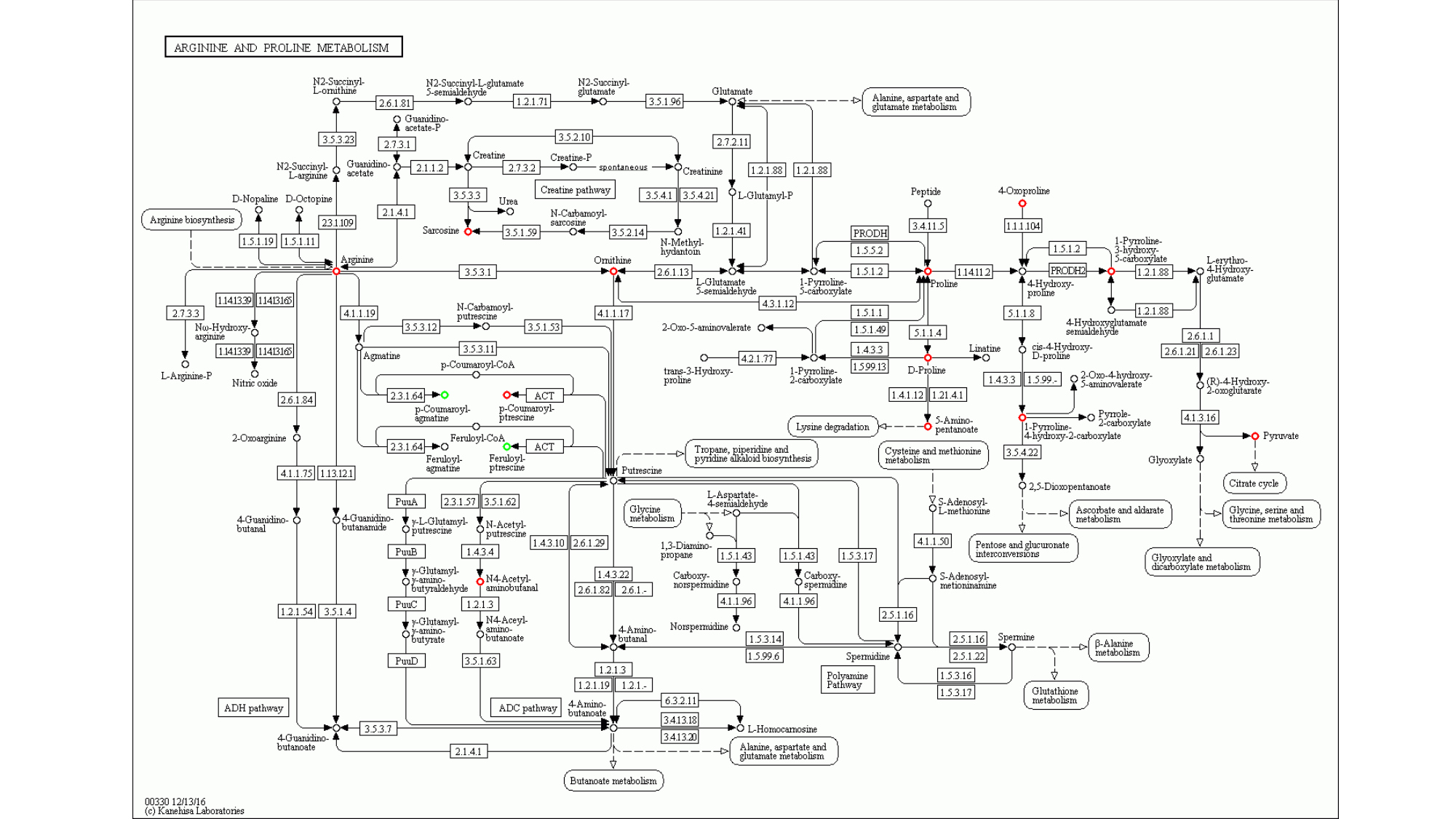

## Slide 6
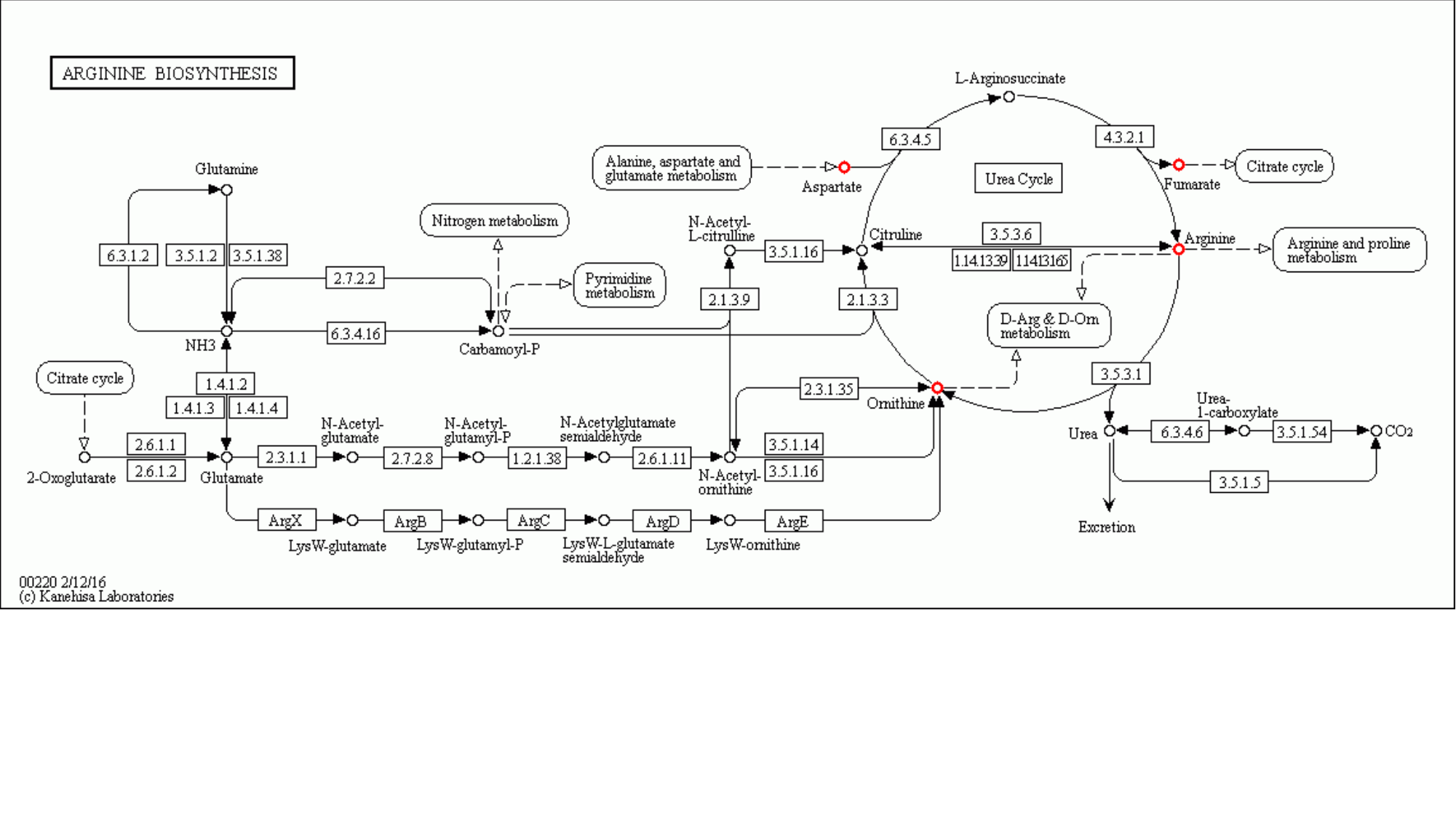

## Slide 7
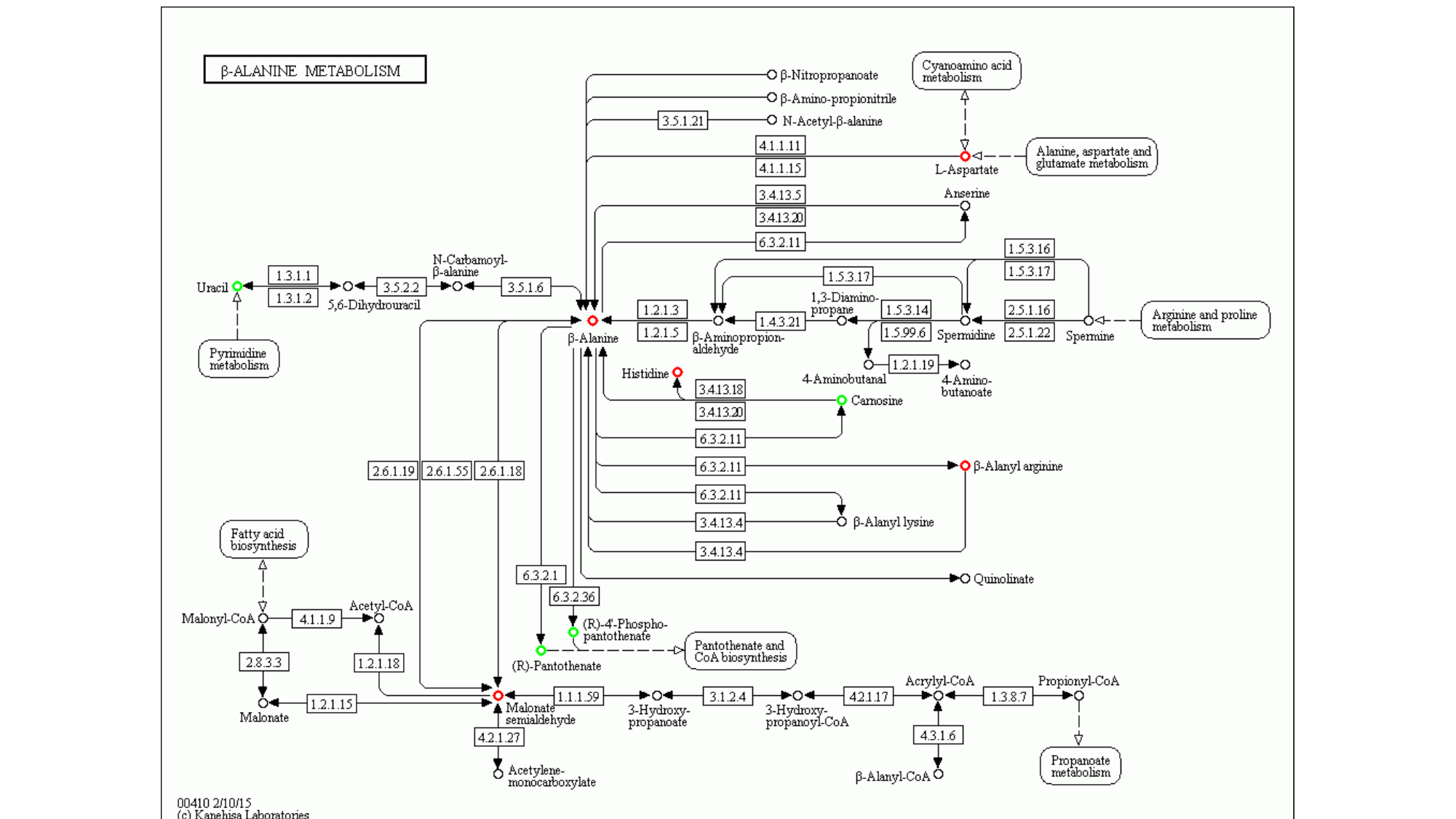

## Slide 8
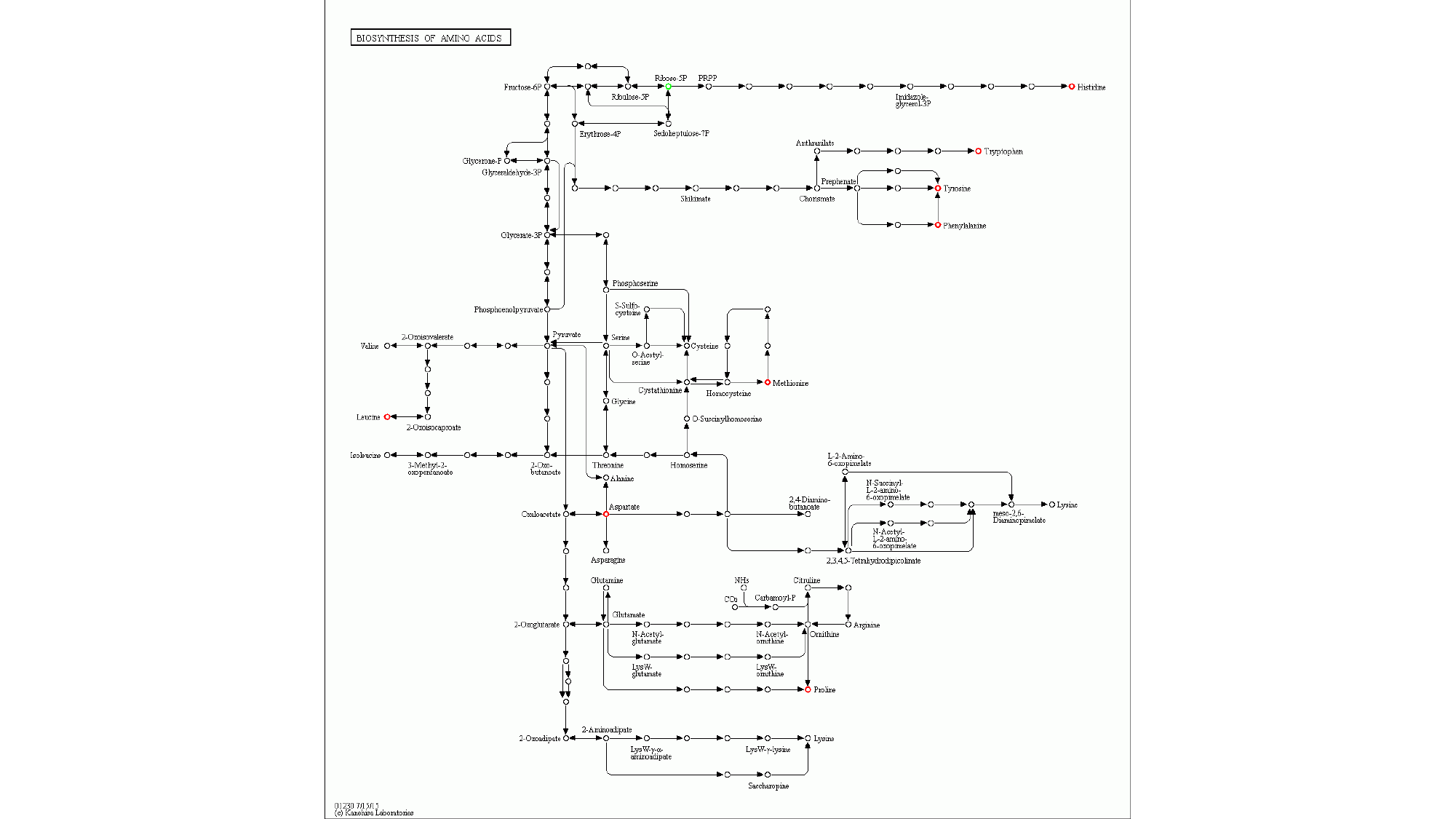

## Slide 9
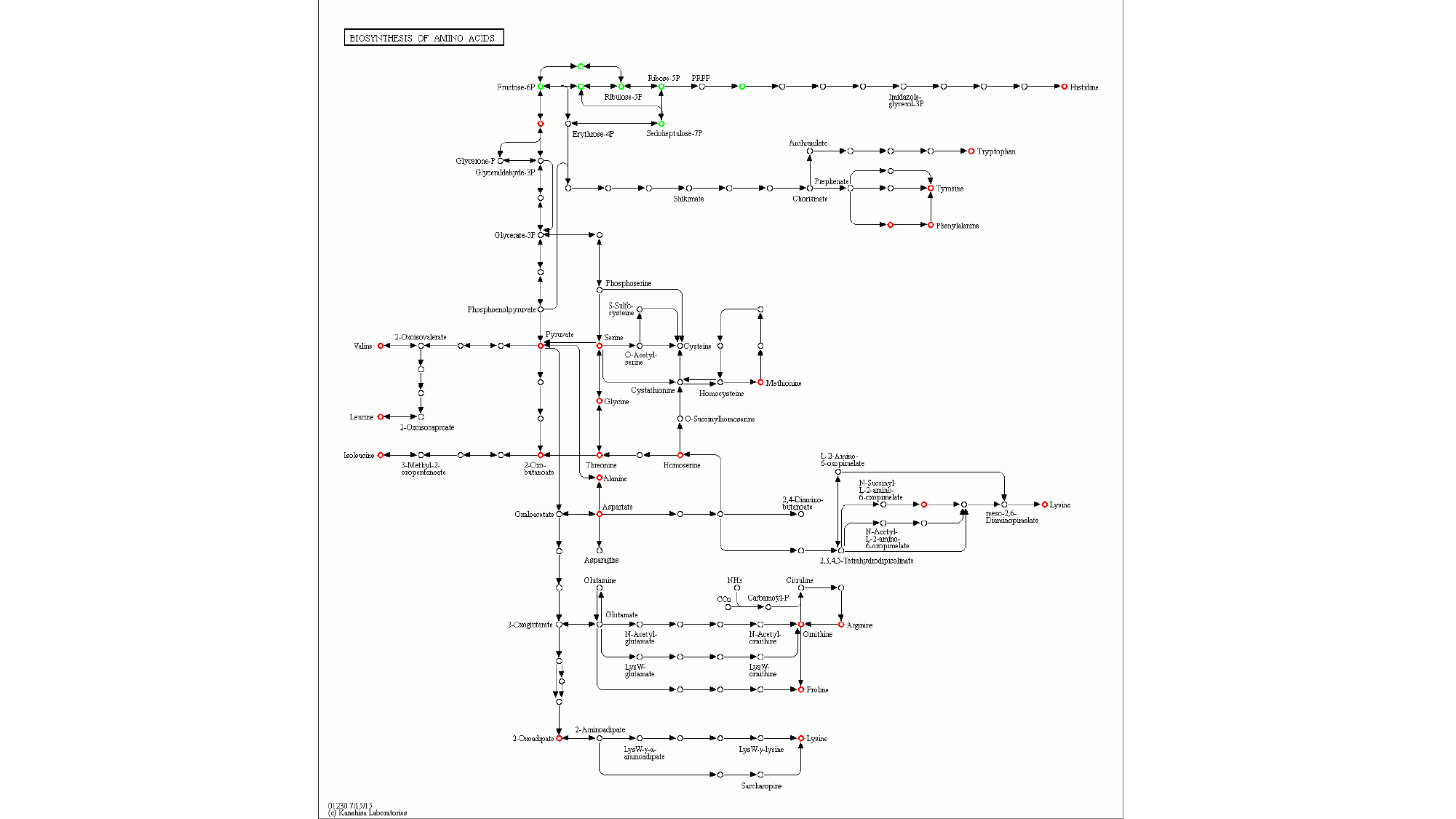

## Slide 10
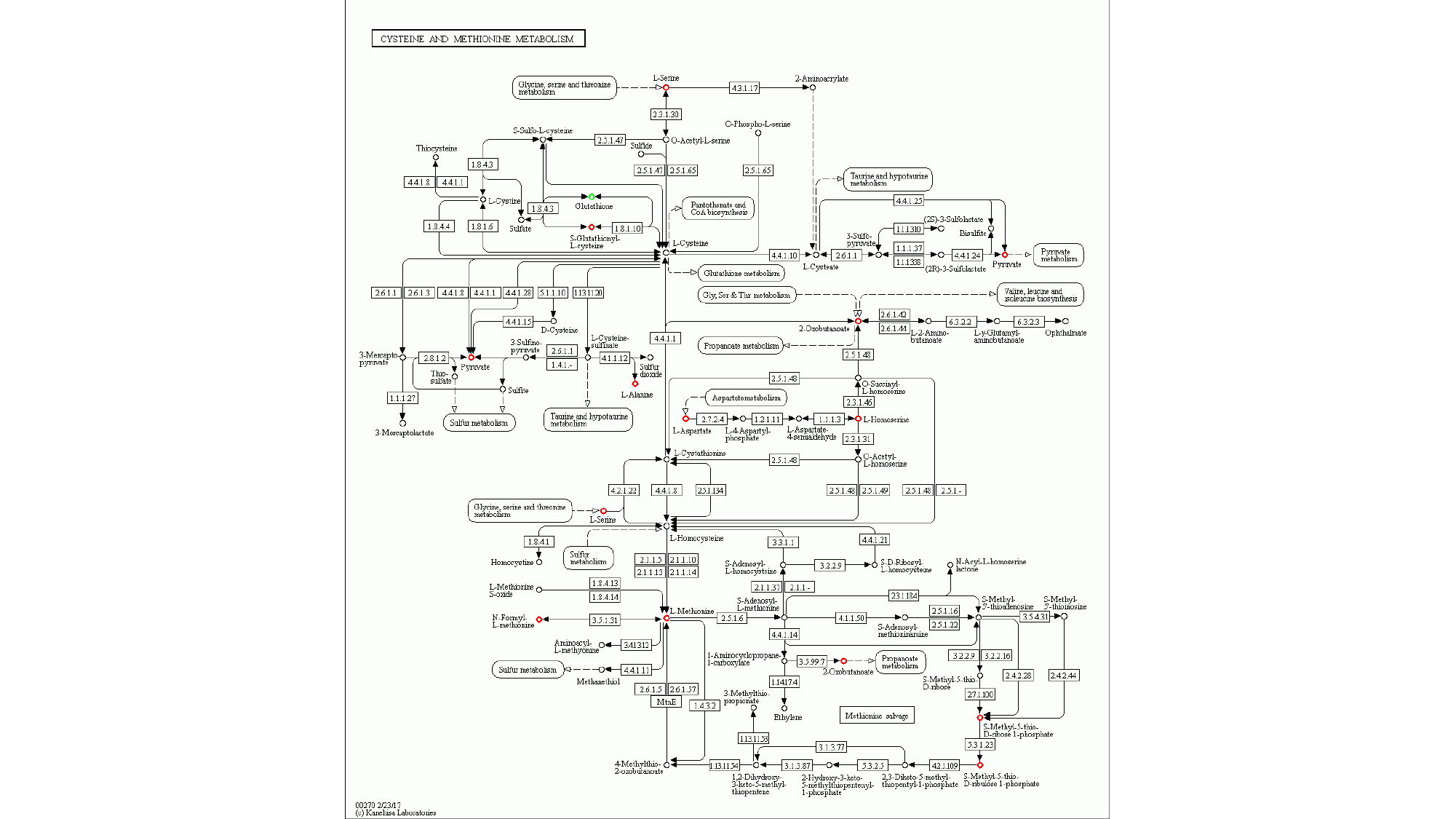

## Slide 11
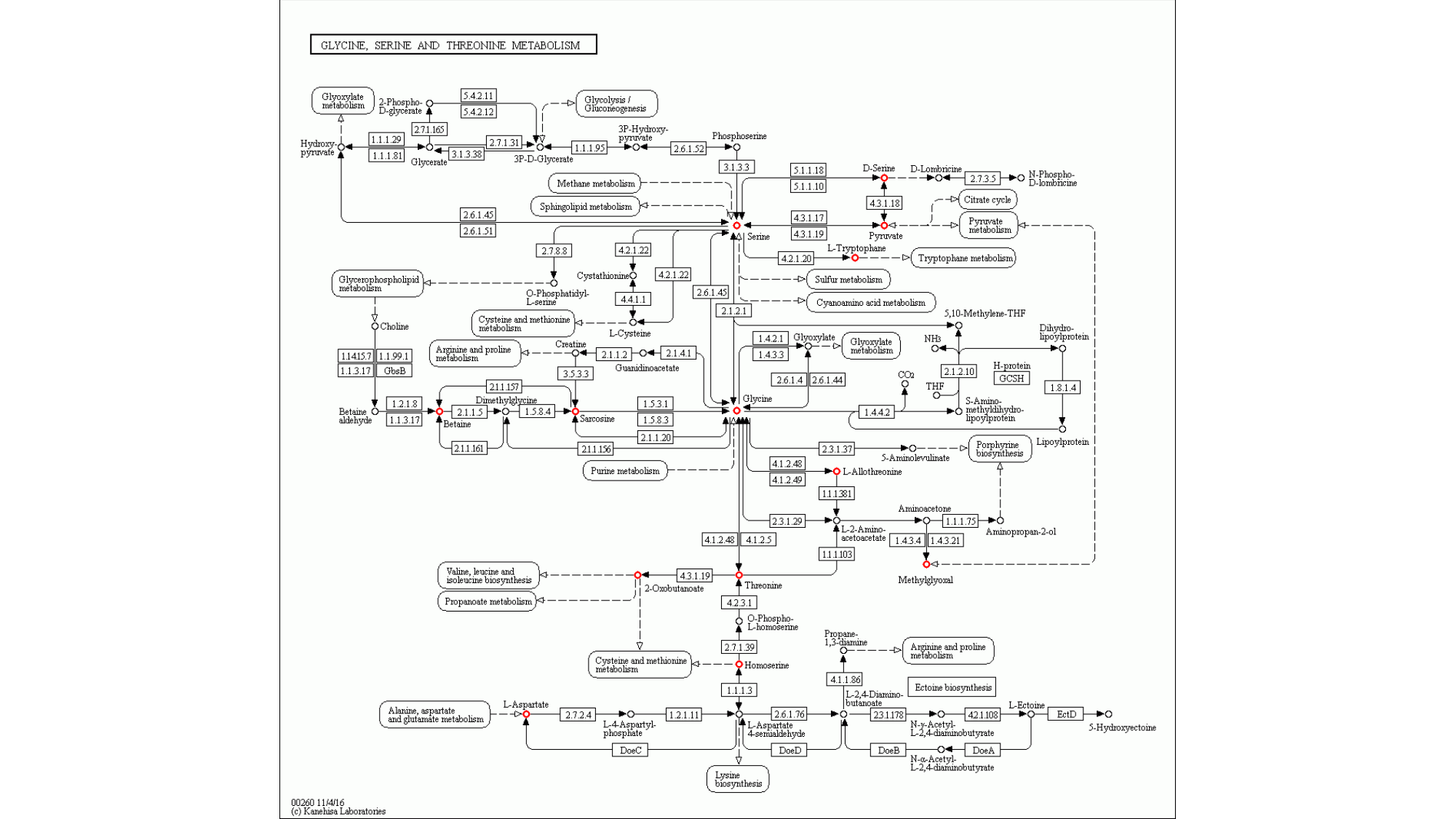

## Slide 12
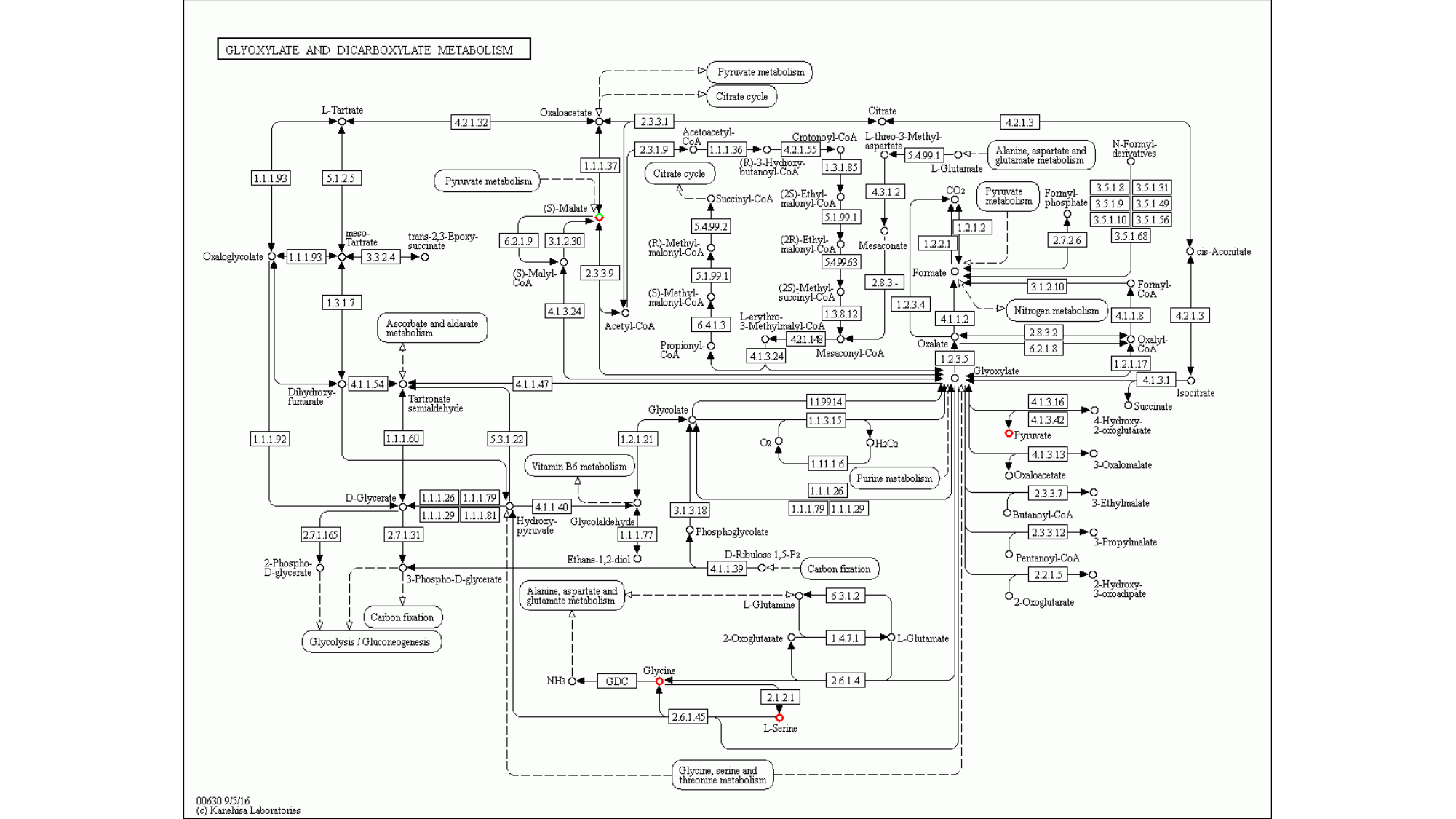

## Slide 13
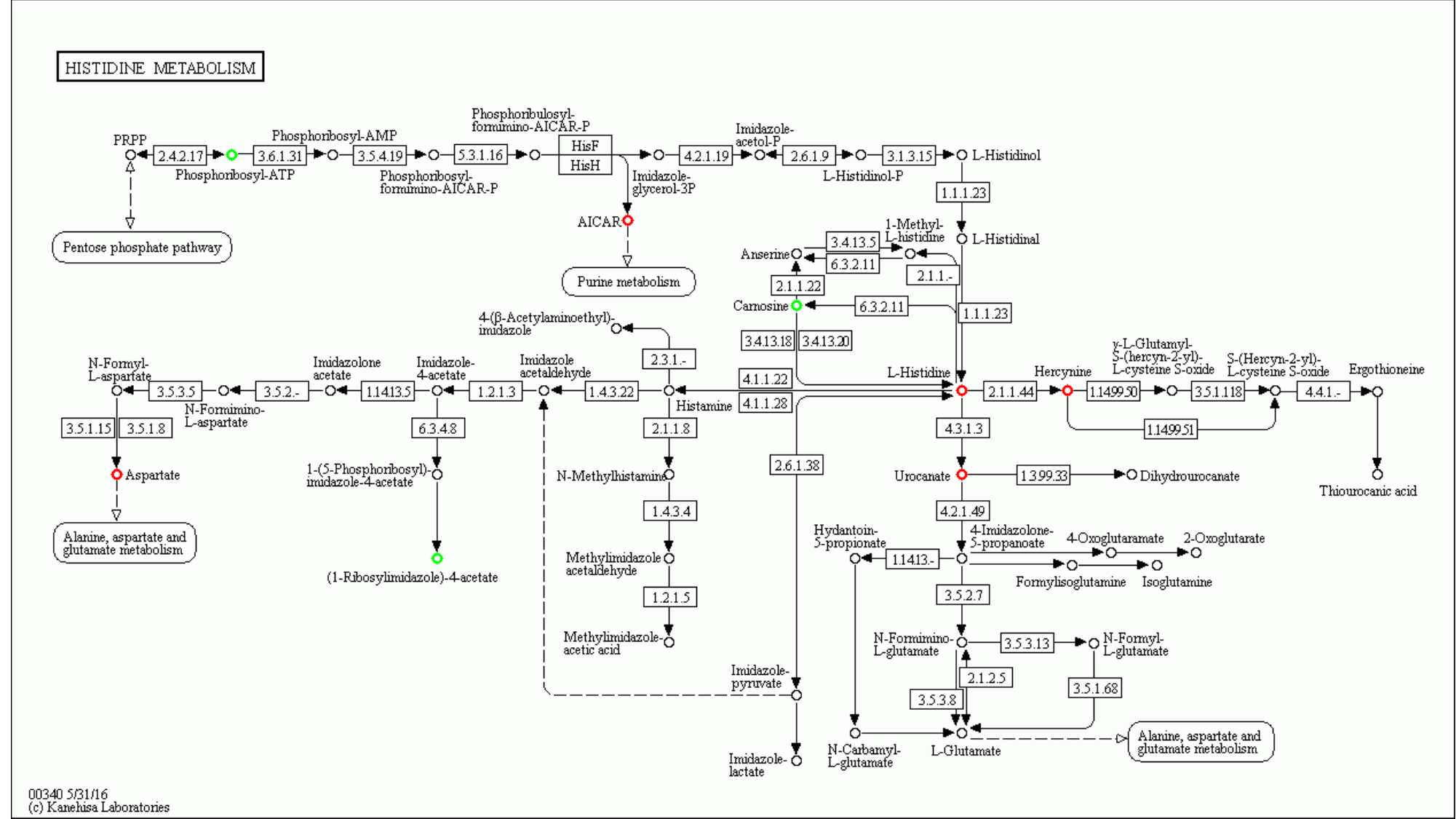

## Slide 14
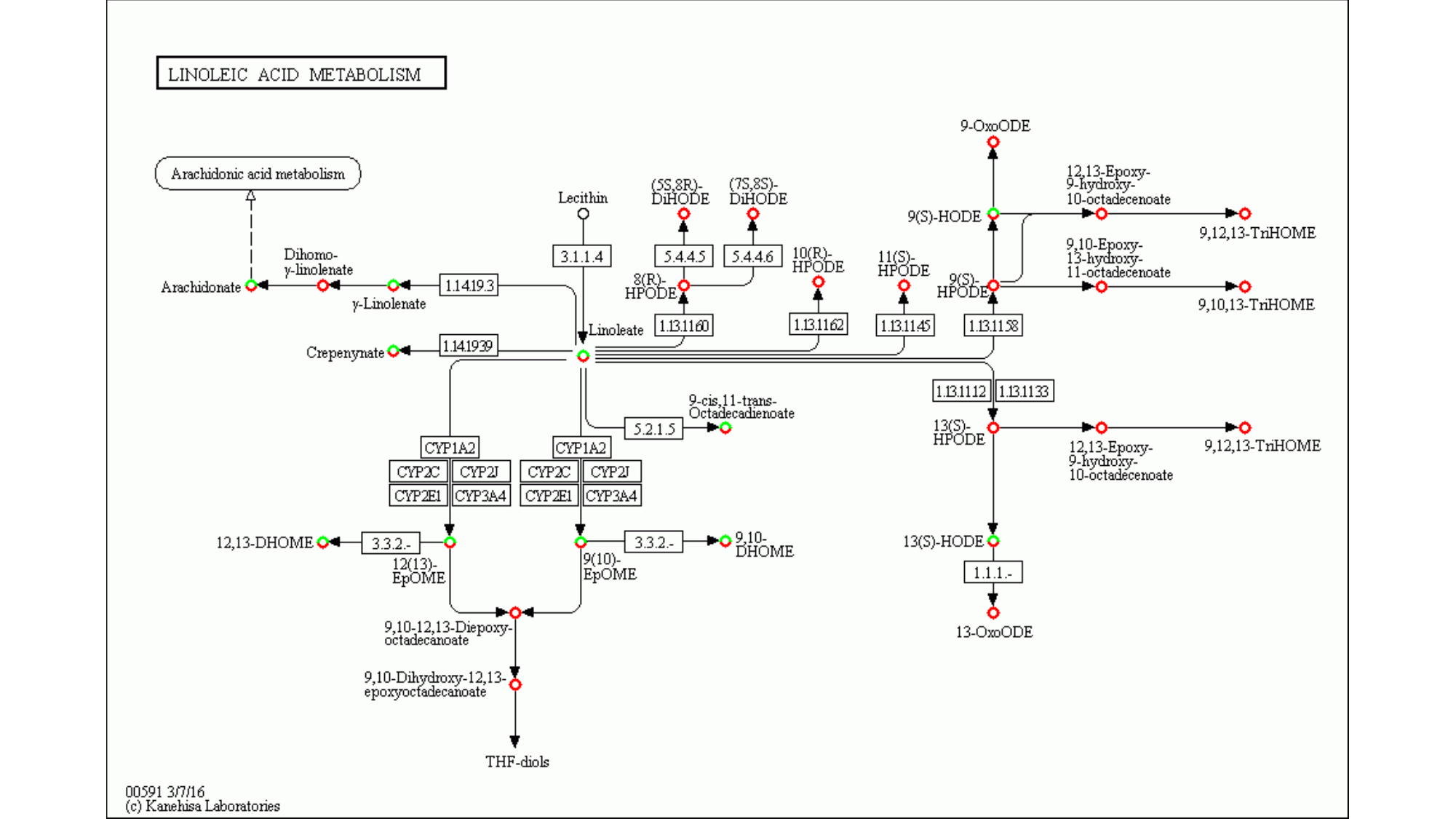

## Slide 15
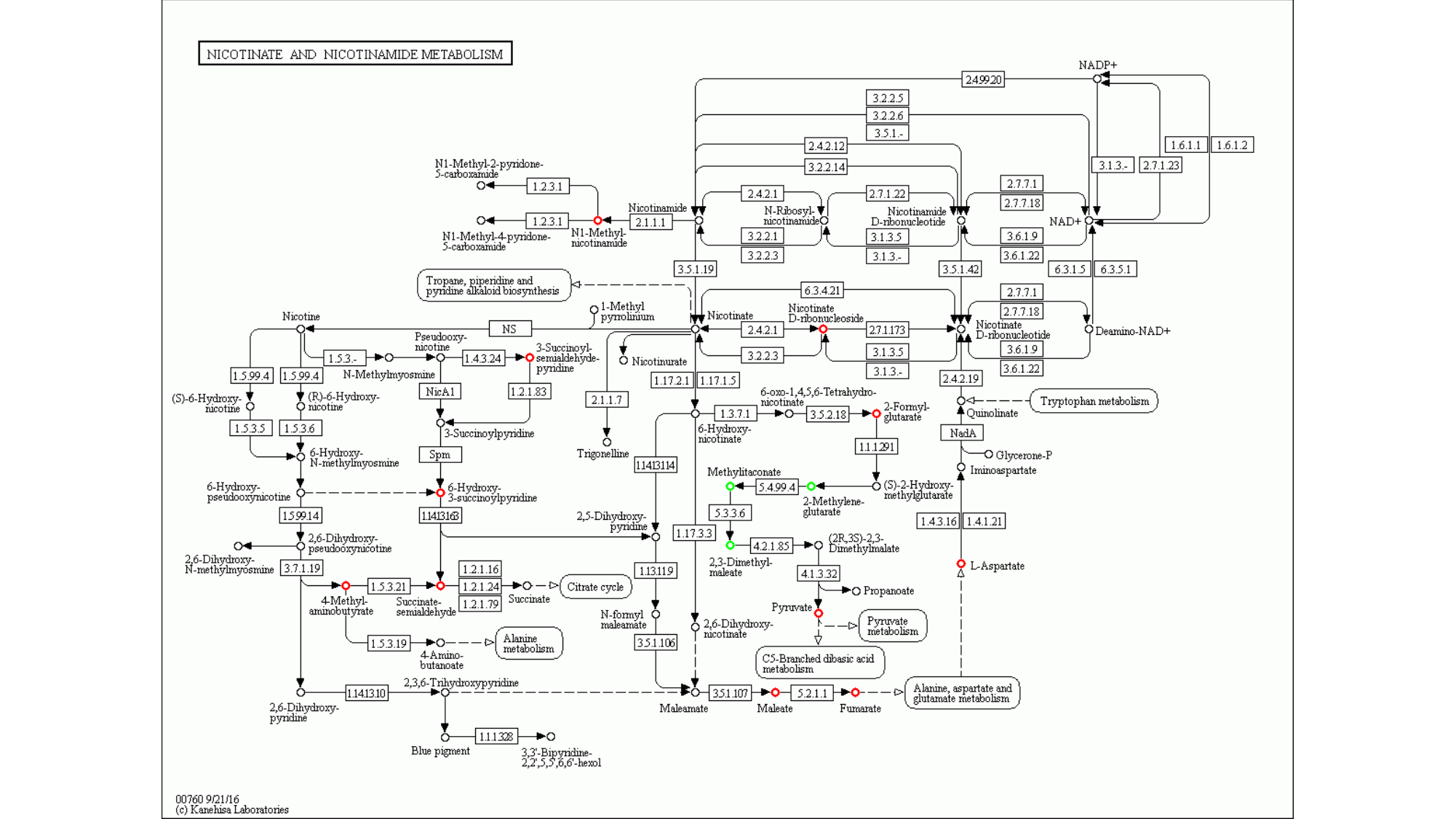

## Slide 16
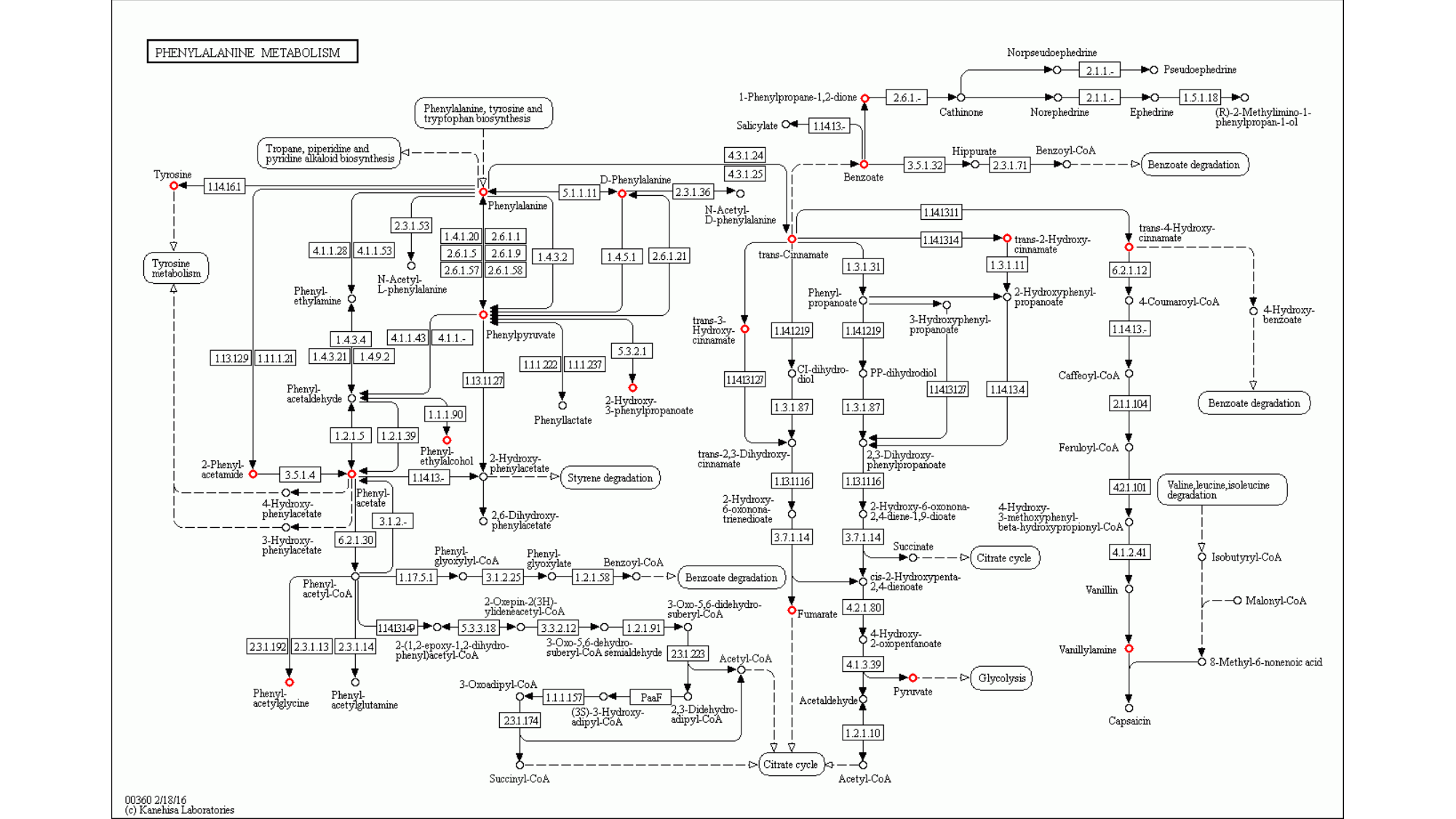

## Slide 17
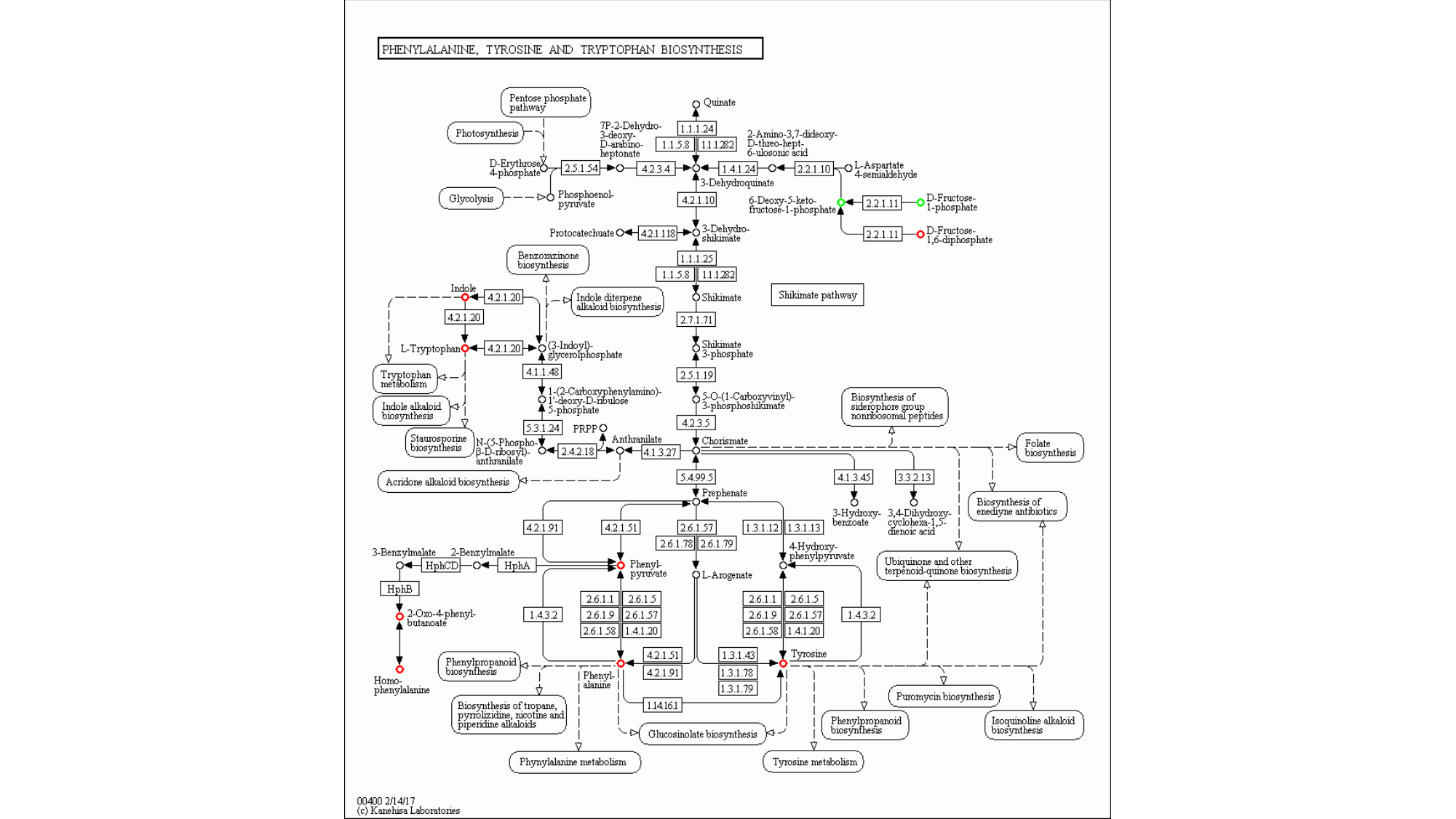

## Slide 18
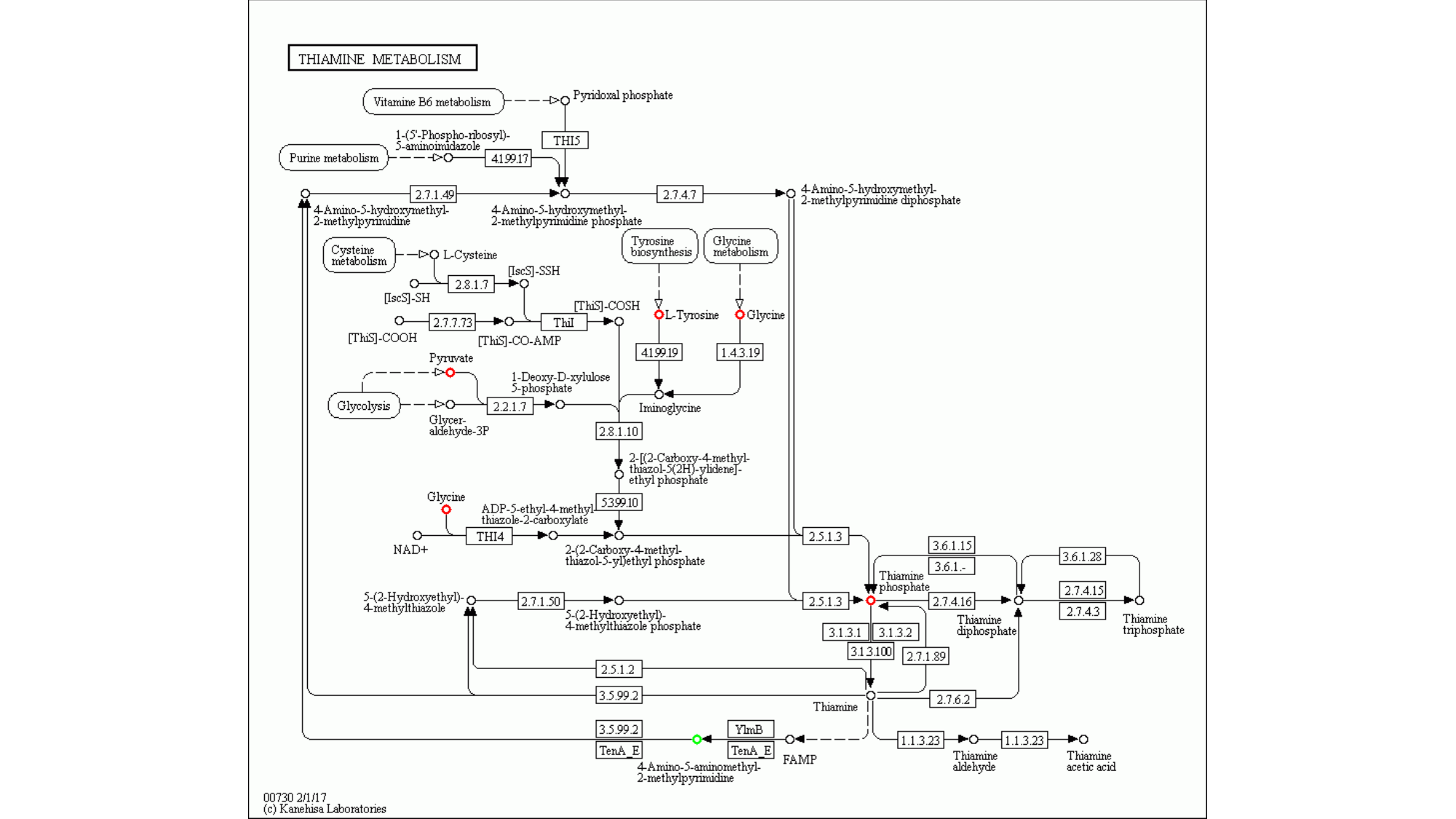

Supplement: Supplementary file 1 — Supplementary Material 1. [file 12864_2024_10329_MOESM1_ESM.zip › Supplementary document-36 Differential (upregulated) metabolite enrichment pathways.pptx]
