# Supplementary material for: Multiomics analysis of platelet-rich plasma promoting biological performance of mesenchymal stem cells
Source: BMC Genomics. 2024 Jun 5;25:564. doi: 10.1186/s12864-024-10329-8 (PMC11151483; doi:10.1186/s12864-024-10329-8)
Supplement: Supplementary file 1 — Supplementary Material 1. [file 12864_2024_10329_MOESM1_ESM.zip › Supplementary document-5 RT-qPCR validation.pptx]

## Slide 1
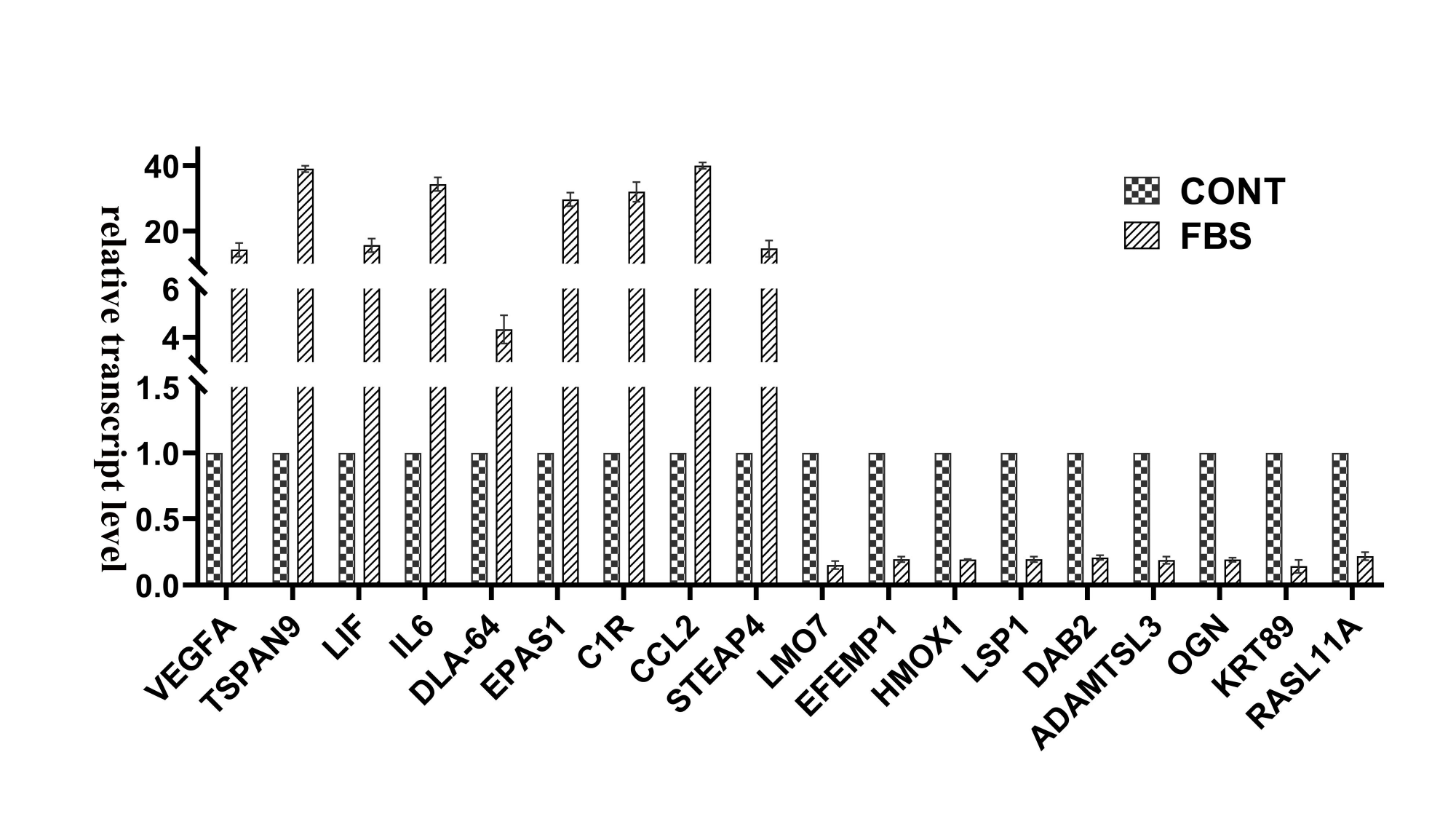

## Slide 2
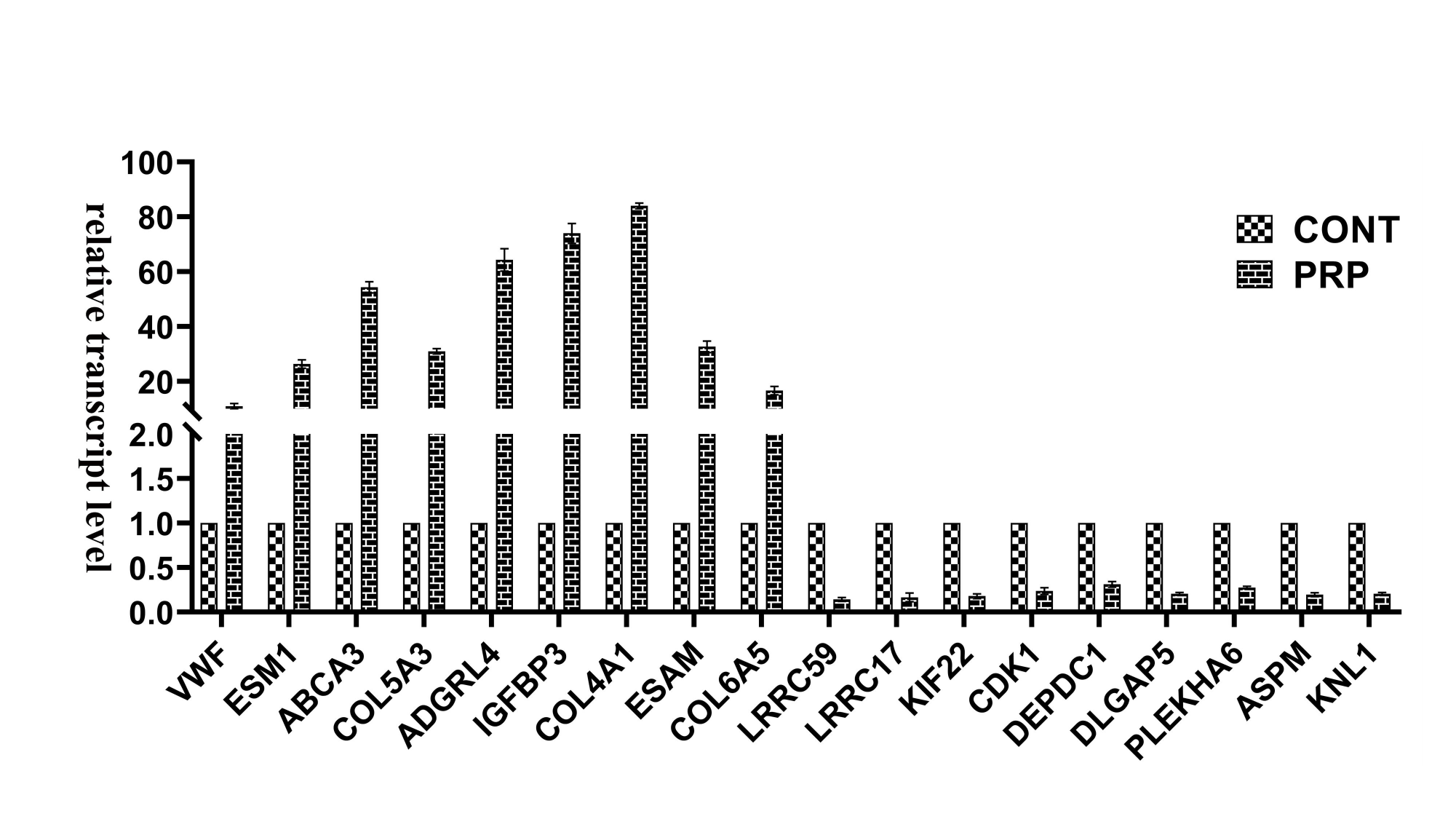

## Slide 3
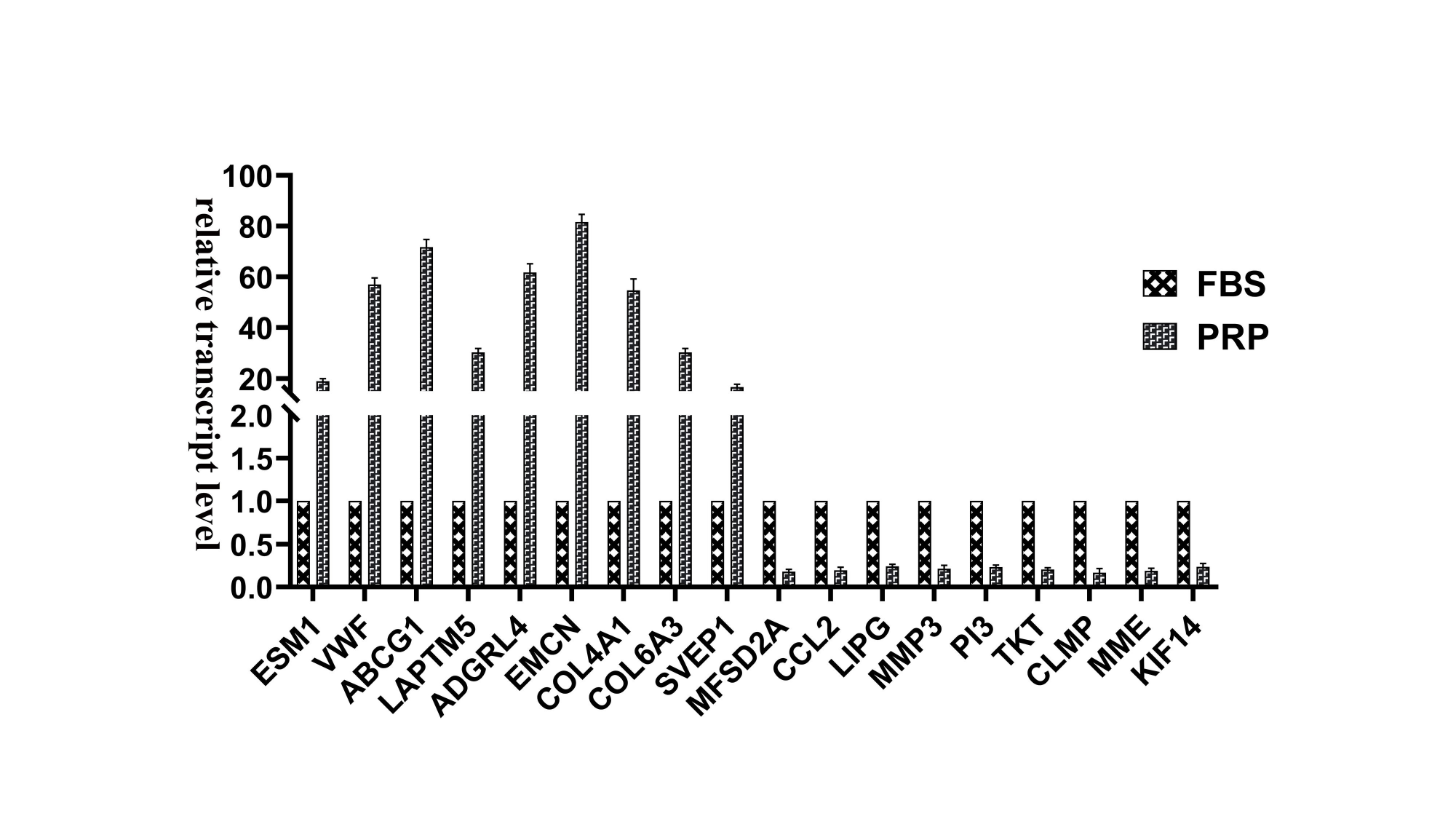

## Slide 4
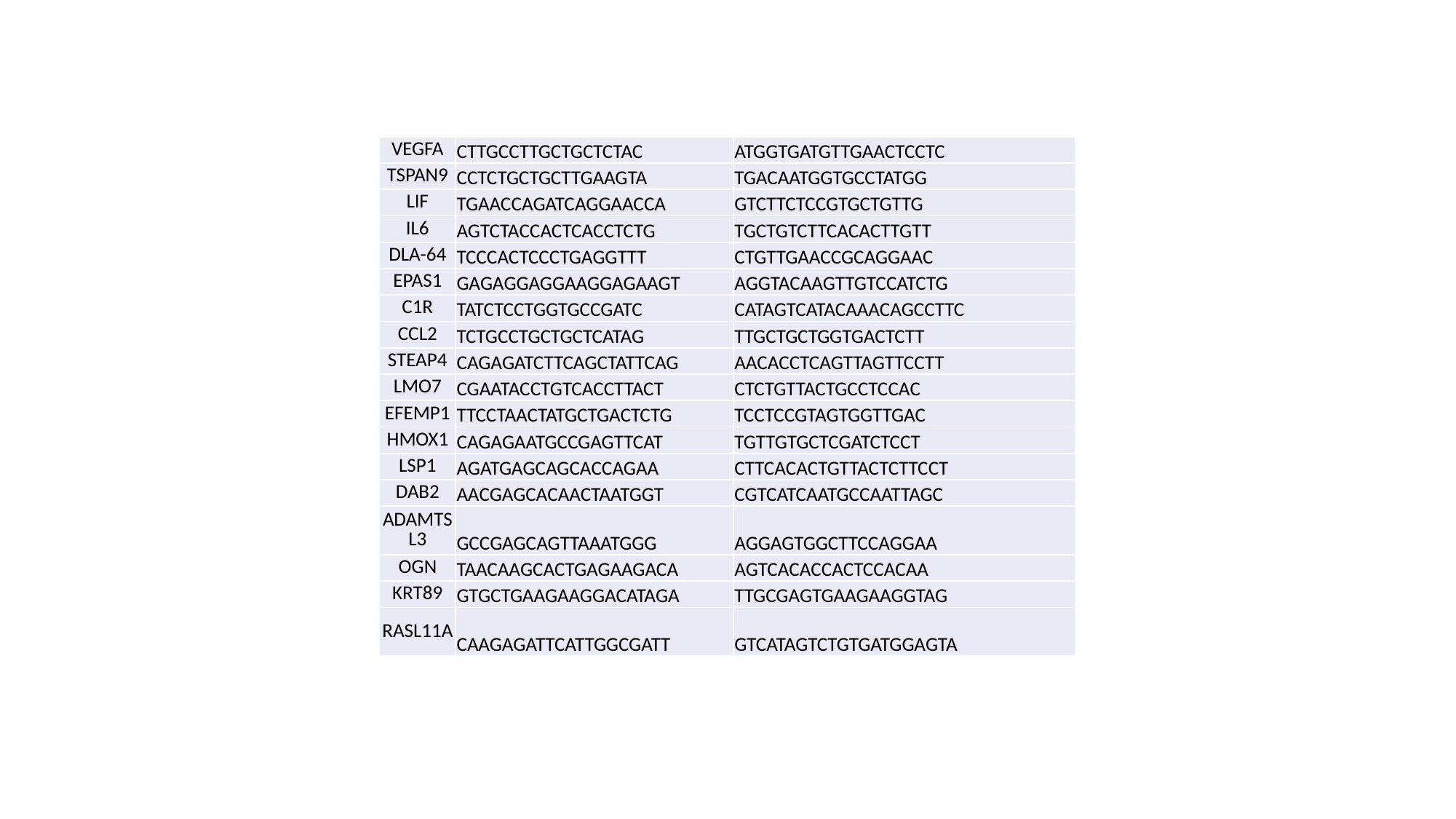

| VEGFA | CTTGCCTTGCTGCTCTAC | ATGGTGATGTTGAACTCCTC |
| --- | --- | --- |
| TSPAN9 | CCTCTGCTGCTTGAAGTA | TGACAATGGTGCCTATGG |
| LIF | TGAACCAGATCAGGAACCA | GTCTTCTCCGTGCTGTTG |
| IL6 | AGTCTACCACTCACCTCTG | TGCTGTCTTCACACTTGTT |
| DLA-64 | TCCCACTCCCTGAGGTTT | CTGTTGAACCGCAGGAAC |
| EPAS1 | GAGAGGAGGAAGGAGAAGT | AGGTACAAGTTGTCCATCTG |
| C1R | TATCTCCTGGTGCCGATC | CATAGTCATACAAACAGCCTTC |
| CCL2 | TCTGCCTGCTGCTCATAG | TTGCTGCTGGTGACTCTT |
| STEAP4 | CAGAGATCTTCAGCTATTCAG | AACACCTCAGTTAGTTCCTT |
| LMO7 | CGAATACCTGTCACCTTACT | CTCTGTTACTGCCTCCAC |
| EFEMP1 | TTCCTAACTATGCTGACTCTG | TCCTCCGTAGTGGTTGAC |
| HMOX1 | CAGAGAATGCCGAGTTCAT | TGTTGTGCTCGATCTCCT |
| LSP1 | AGATGAGCAGCACCAGAA | CTTCACACTGTTACTCTTCCT |
| DAB2 | AACGAGCACAACTAATGGT | CGTCATCAATGCCAATTAGC |
| ADAMTSL3 | GCCGAGCAGTTAAATGGG | AGGAGTGGCTTCCAGGAA |
| OGN | TAACAAGCACTGAGAAGACA | AGTCACACCACTCCACAA |
| KRT89 | GTGCTGAAGAAGGACATAGA | TTGCGAGTGAAGAAGGTAG |
| RASL11A | CAAGAGATTCATTGGCGATT | GTCATAGTCTGTGATGGAGTA |

## Slide 5
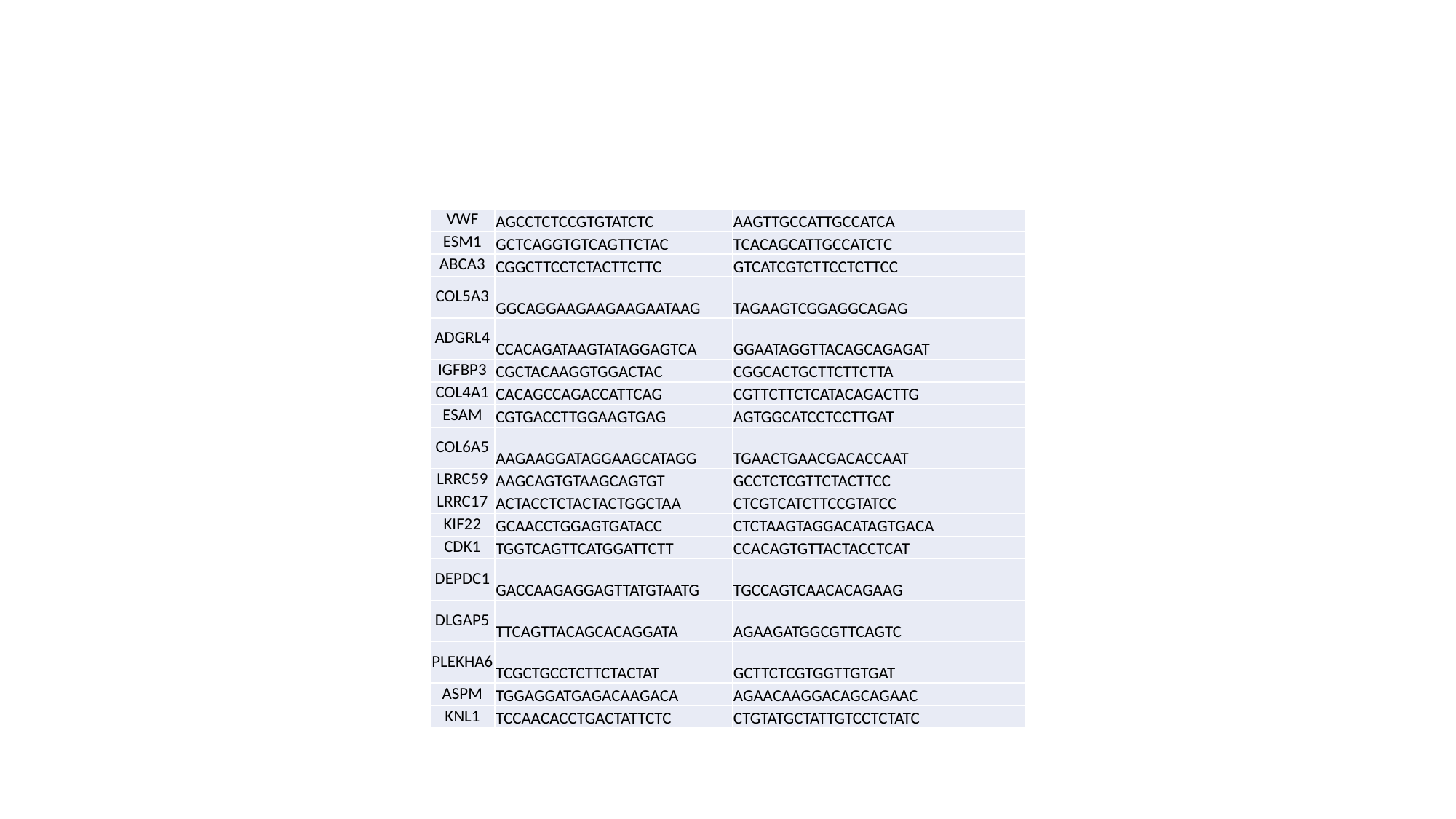

| VWF | AGCCTCTCCGTGTATCTC | AAGTTGCCATTGCCATCA |
| --- | --- | --- |
| ESM1 | GCTCAGGTGTCAGTTCTAC | TCACAGCATTGCCATCTC |
| ABCA3 | CGGCTTCCTCTACTTCTTC | GTCATCGTCTTCCTCTTCC |
| COL5A3 | GGCAGGAAGAAGAAGAATAAG | TAGAAGTCGGAGGCAGAG |
| ADGRL4 | CCACAGATAAGTATAGGAGTCA | GGAATAGGTTACAGCAGAGAT |
| IGFBP3 | CGCTACAAGGTGGACTAC | CGGCACTGCTTCTTCTTA |
| COL4A1 | CACAGCCAGACCATTCAG | CGTTCTTCTCATACAGACTTG |
| ESAM | CGTGACCTTGGAAGTGAG | AGTGGCATCCTCCTTGAT |
| COL6A5 | AAGAAGGATAGGAAGCATAGG | TGAACTGAACGACACCAAT |
| LRRC59 | AAGCAGTGTAAGCAGTGT | GCCTCTCGTTCTACTTCC |
| LRRC17 | ACTACCTCTACTACTGGCTAA | CTCGTCATCTTCCGTATCC |
| KIF22 | GCAACCTGGAGTGATACC | CTCTAAGTAGGACATAGTGACA |
| CDK1 | TGGTCAGTTCATGGATTCTT | CCACAGTGTTACTACCTCAT |
| DEPDC1 | GACCAAGAGGAGTTATGTAATG | TGCCAGTCAACACAGAAG |
| DLGAP5 | TTCAGTTACAGCACAGGATA | AGAAGATGGCGTTCAGTC |
| PLEKHA6 | TCGCTGCCTCTTCTACTAT | GCTTCTCGTGGTTGTGAT |
| ASPM | TGGAGGATGAGACAAGACA | AGAACAAGGACAGCAGAAC |
| KNL1 | TCCAACACCTGACTATTCTC | CTGTATGCTATTGTCCTCTATC |

## Slide 6
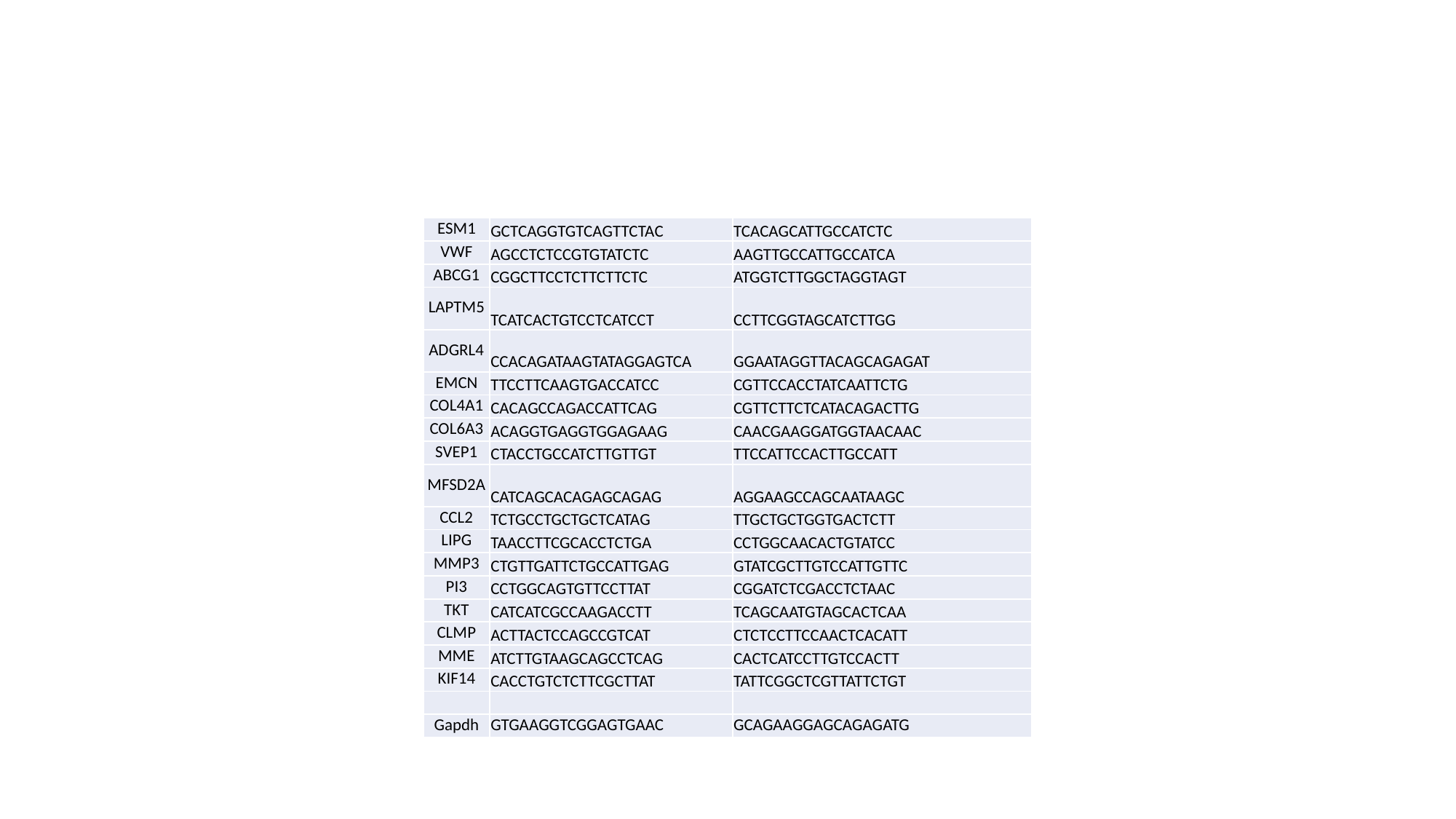

| ESM1 | GCTCAGGTGTCAGTTCTAC | TCACAGCATTGCCATCTC |
| --- | --- | --- |
| VWF | AGCCTCTCCGTGTATCTC | AAGTTGCCATTGCCATCA |
| ABCG1 | CGGCTTCCTCTTCTTCTC | ATGGTCTTGGCTAGGTAGT |
| LAPTM5 | TCATCACTGTCCTCATCCT | CCTTCGGTAGCATCTTGG |
| ADGRL4 | CCACAGATAAGTATAGGAGTCA | GGAATAGGTTACAGCAGAGAT |
| EMCN | TTCCTTCAAGTGACCATCC | CGTTCCACCTATCAATTCTG |
| COL4A1 | CACAGCCAGACCATTCAG | CGTTCTTCTCATACAGACTTG |
| COL6A3 | ACAGGTGAGGTGGAGAAG | CAACGAAGGATGGTAACAAC |
| SVEP1 | CTACCTGCCATCTTGTTGT | TTCCATTCCACTTGCCATT |
| MFSD2A | CATCAGCACAGAGCAGAG | AGGAAGCCAGCAATAAGC |
| CCL2 | TCTGCCTGCTGCTCATAG | TTGCTGCTGGTGACTCTT |
| LIPG | TAACCTTCGCACCTCTGA | CCTGGCAACACTGTATCC |
| MMP3 | CTGTTGATTCTGCCATTGAG | GTATCGCTTGTCCATTGTTC |
| PI3 | CCTGGCAGTGTTCCTTAT | CGGATCTCGACCTCTAAC |
| TKT | CATCATCGCCAAGACCTT | TCAGCAATGTAGCACTCAA |
| CLMP | ACTTACTCCAGCCGTCAT | CTCTCCTTCCAACTCACATT |
| MME | ATCTTGTAAGCAGCCTCAG | CACTCATCCTTGTCCACTT |
| KIF14 | CACCTGTCTCTTCGCTTAT | TATTCGGCTCGTTATTCTGT |
| | | |
| Gapdh | GTGAAGGTCGGAGTGAAC | GCAGAAGGAGCAGAGATG |
