# Supplementary figures and images for: Multiomics analysis of platelet-rich plasma promoting biological performance of mesenchymal stem cells
Source: BMC Genomics. 2024 Jun 5;25:564. doi: 10.1186/s12864-024-10329-8 (PMC11151483; doi:10.1186/s12864-024-10329-8)

## Slide 1
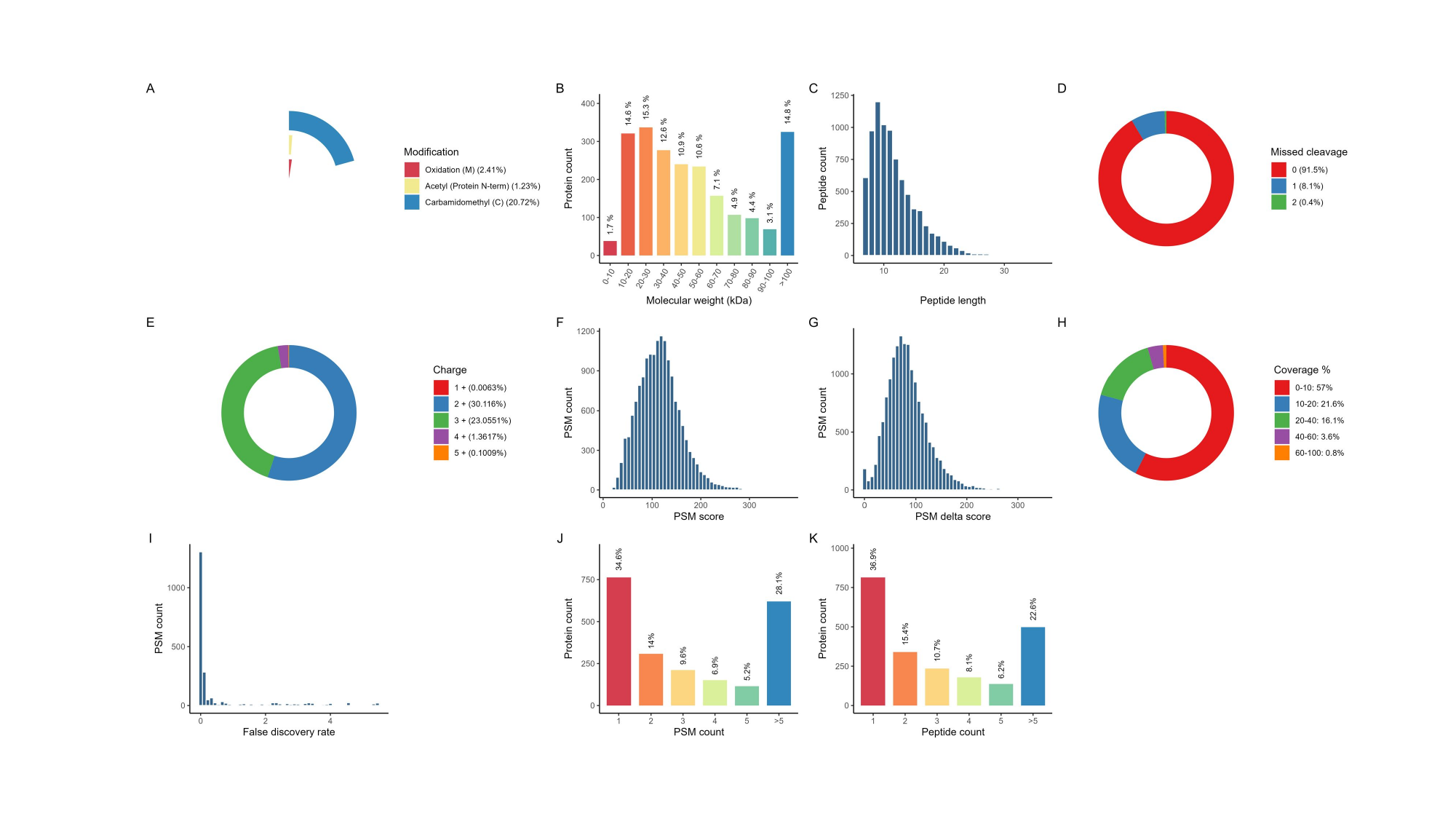

Supplement: Supplementary file 1 — Supplementary Material 1. [file 12864_2024_10329_MOESM1_ESM.zip › Supplementary document-13 identification.qc.combine.pptx]

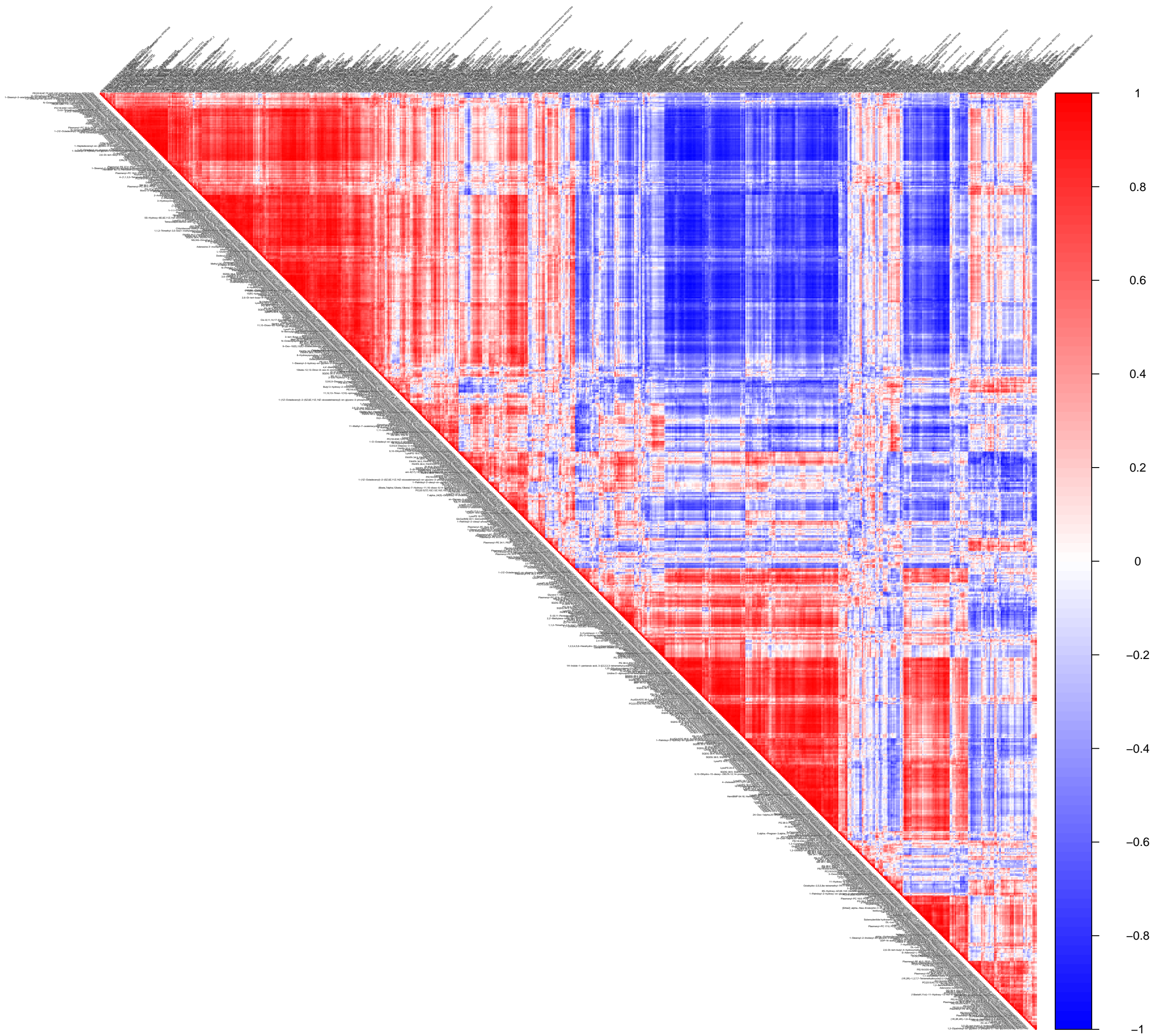

Supplement: Supplementary file 1 — Supplementary Material 1. [file 12864_2024_10329_MOESM1_ESM.zip › Supplementary document-26 combine.metabolite.correlation.pdf]

## Slide 1
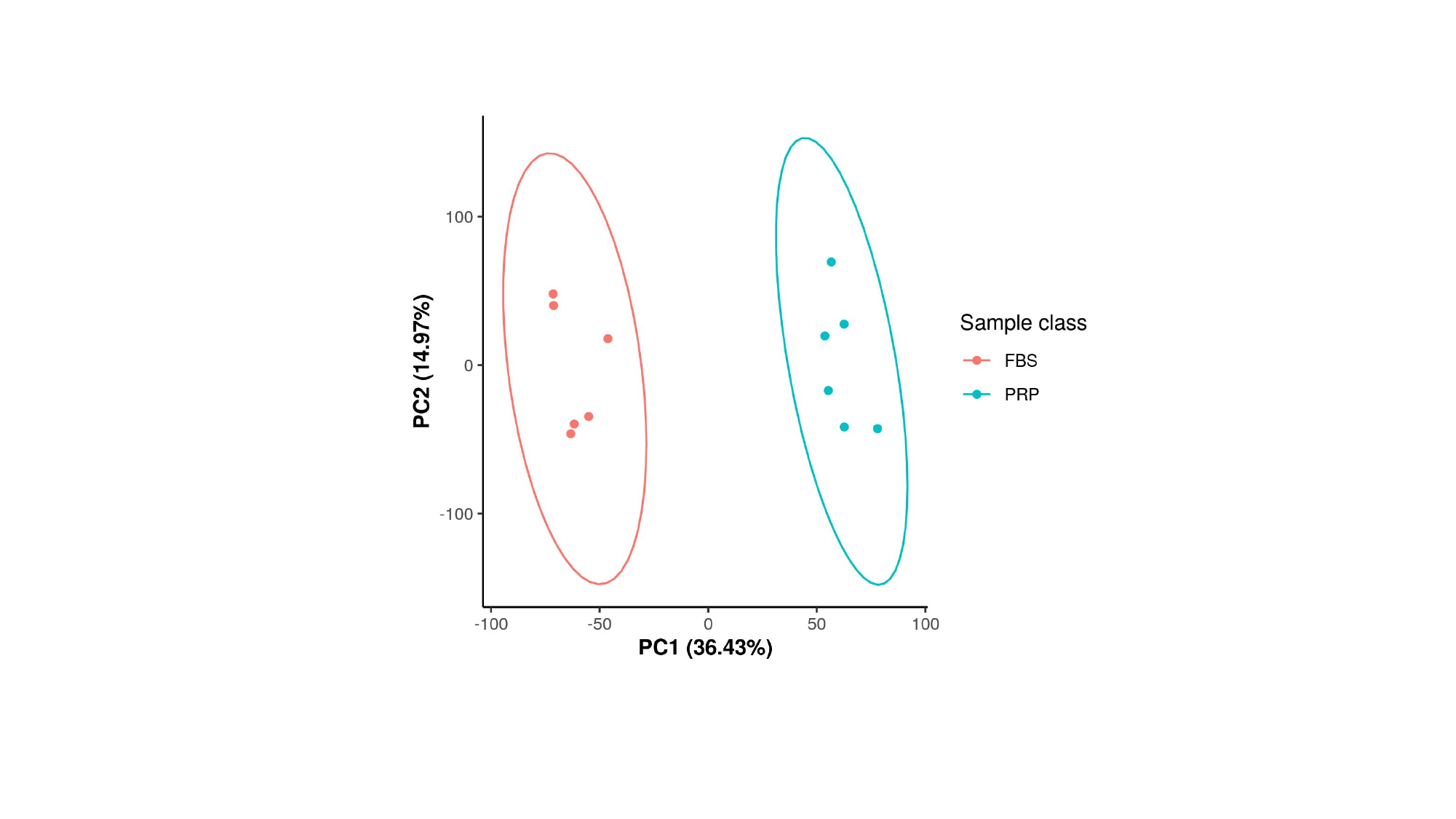

Supplement: Supplementary file 1 — Supplementary Material 1. [file 12864_2024_10329_MOESM1_ESM.zip › Supplementary document-29 PRP_FBS.pca.score.noqc.pptx]

## Slide 1
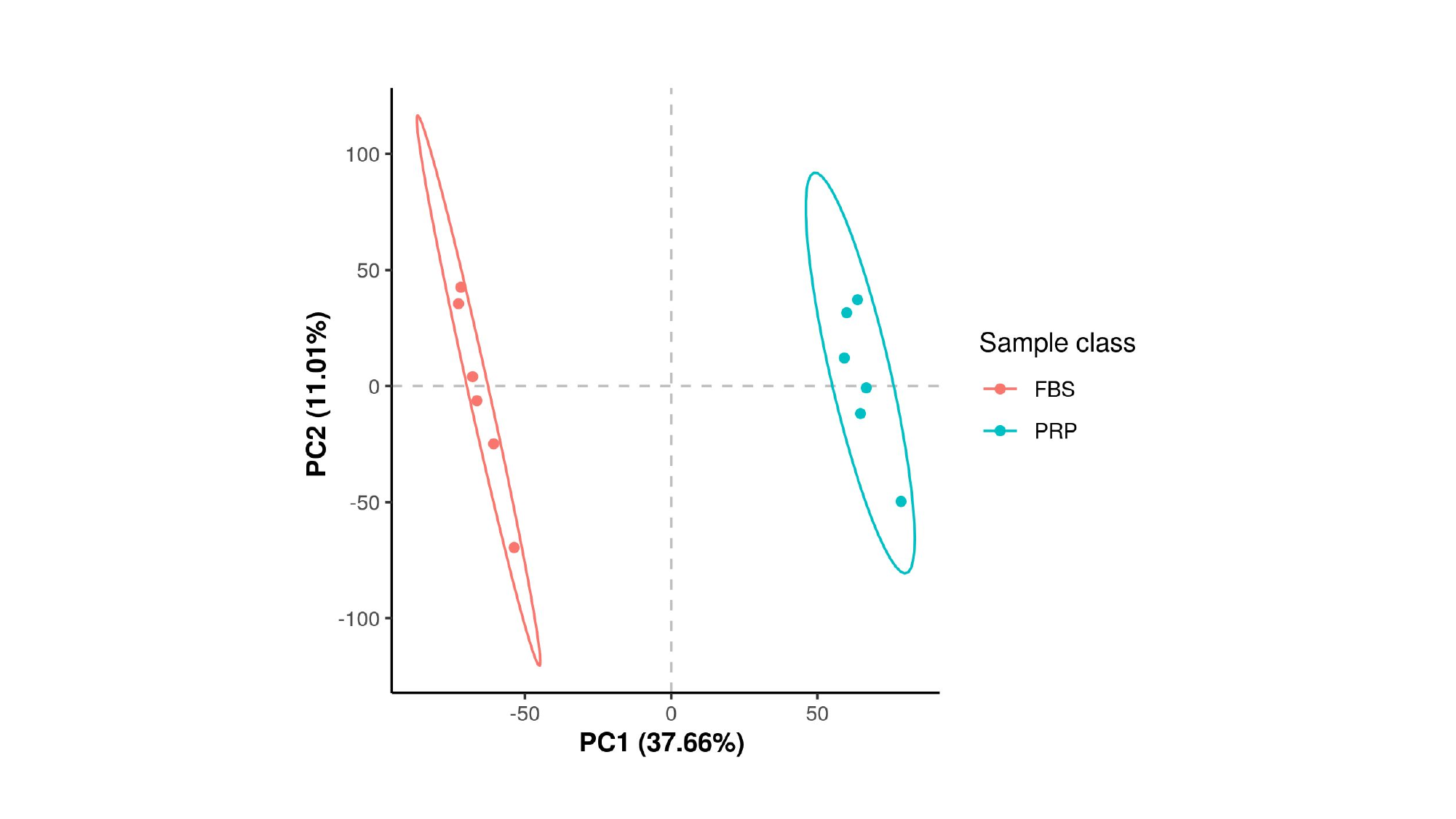

Supplement: Supplementary file 1 — Supplementary Material 1. [file 12864_2024_10329_MOESM1_ESM.zip › Supplementary document-30 PRP_FBS.plsda.score.pptx]

## Slide 1
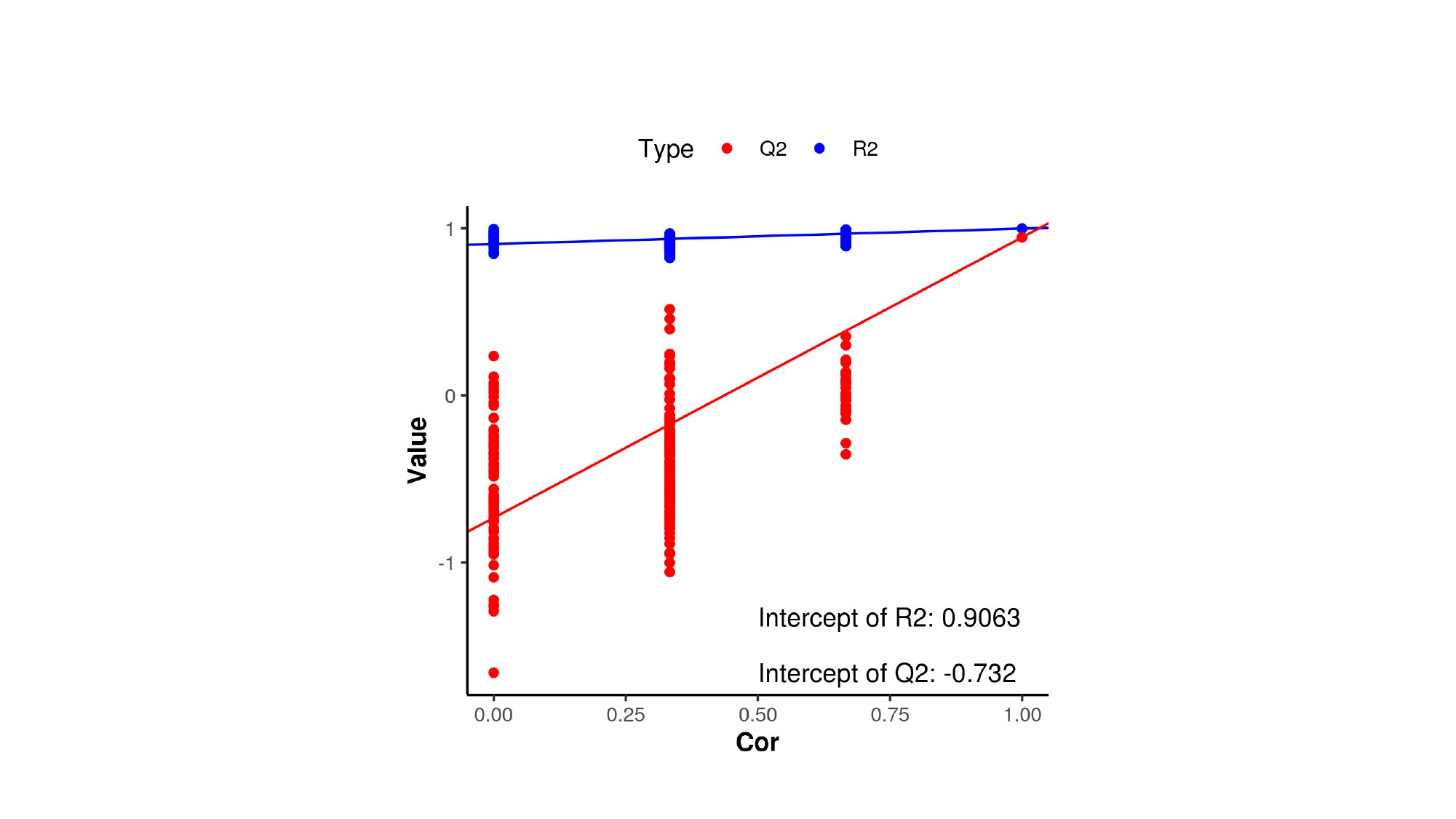

Supplement: Supplementary file 1 — Supplementary Material 1. [file 12864_2024_10329_MOESM1_ESM.zip › Supplementary document-31 PRP_FBS.plsda.validation.pptx]

## Slide 1
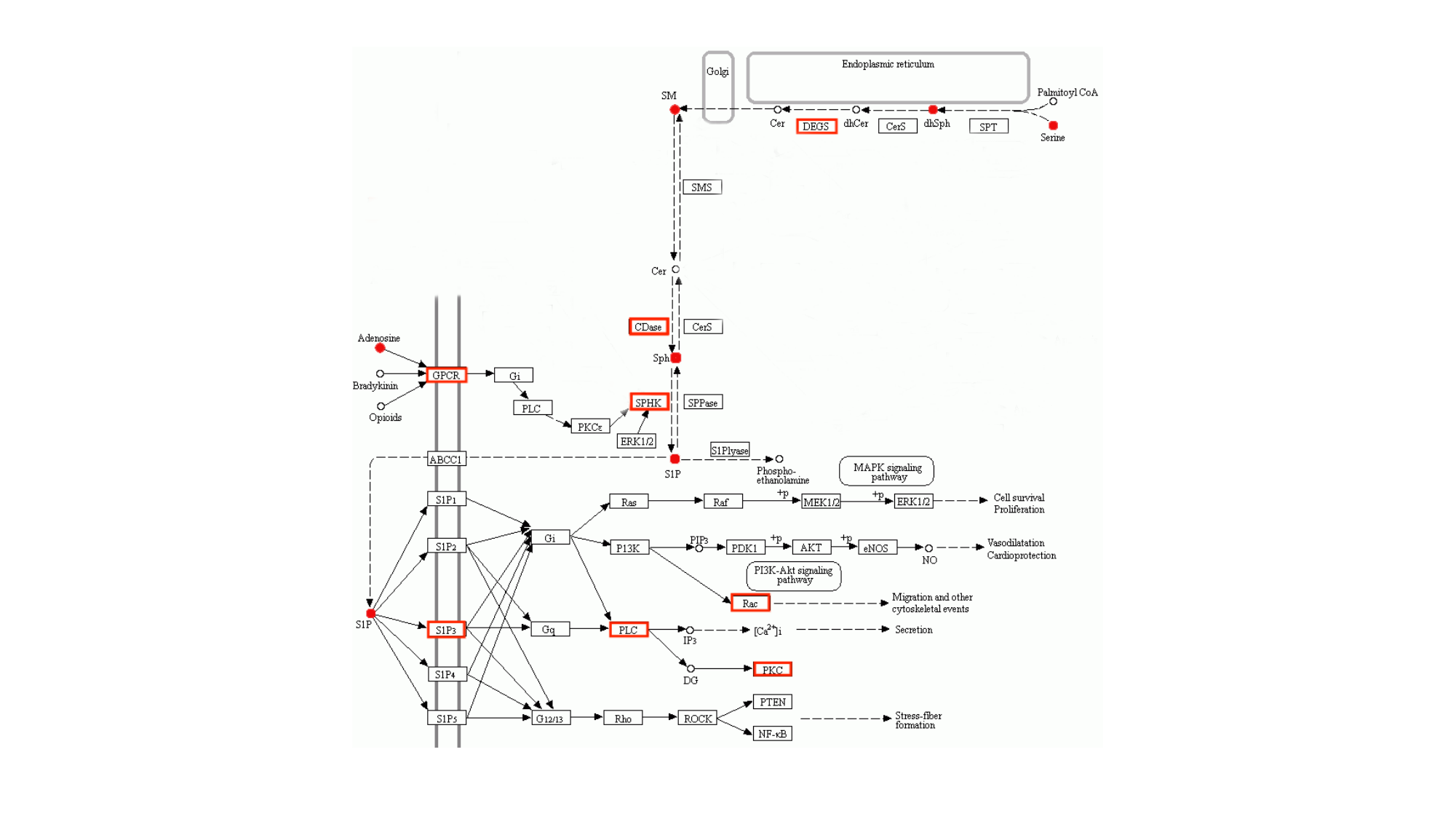

Supplement: Supplementary file 1 — Supplementary Material 1. [file 12864_2024_10329_MOESM1_ESM.zip › Supplementary document-34 Sphingolipid signaling pathway.pptx]

## Slide 1
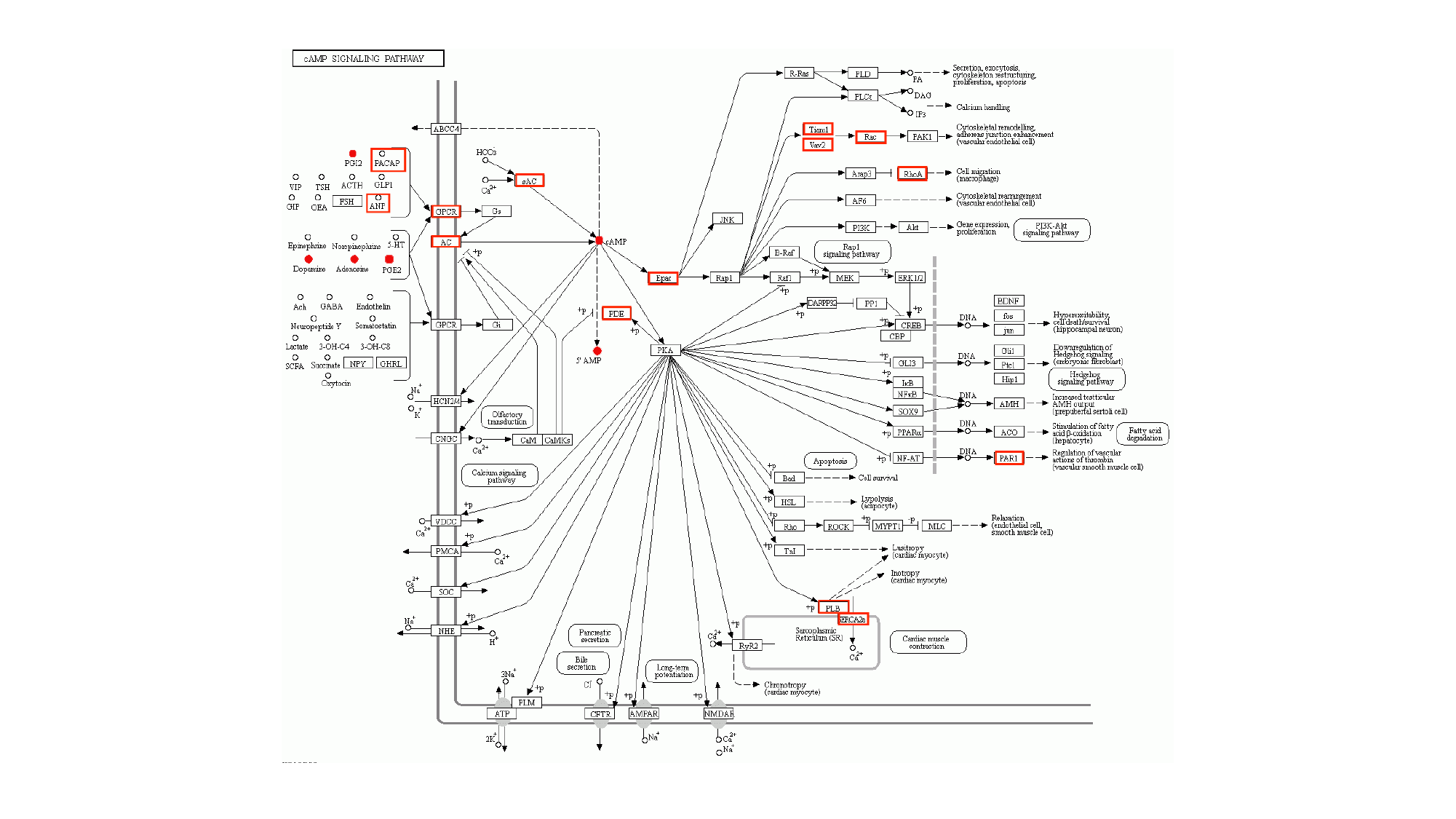

Supplement: Supplementary file 1 — Supplementary Material 1. [file 12864_2024_10329_MOESM1_ESM.zip › Supplementary document-35 cAMP signaling pathway.pptx]

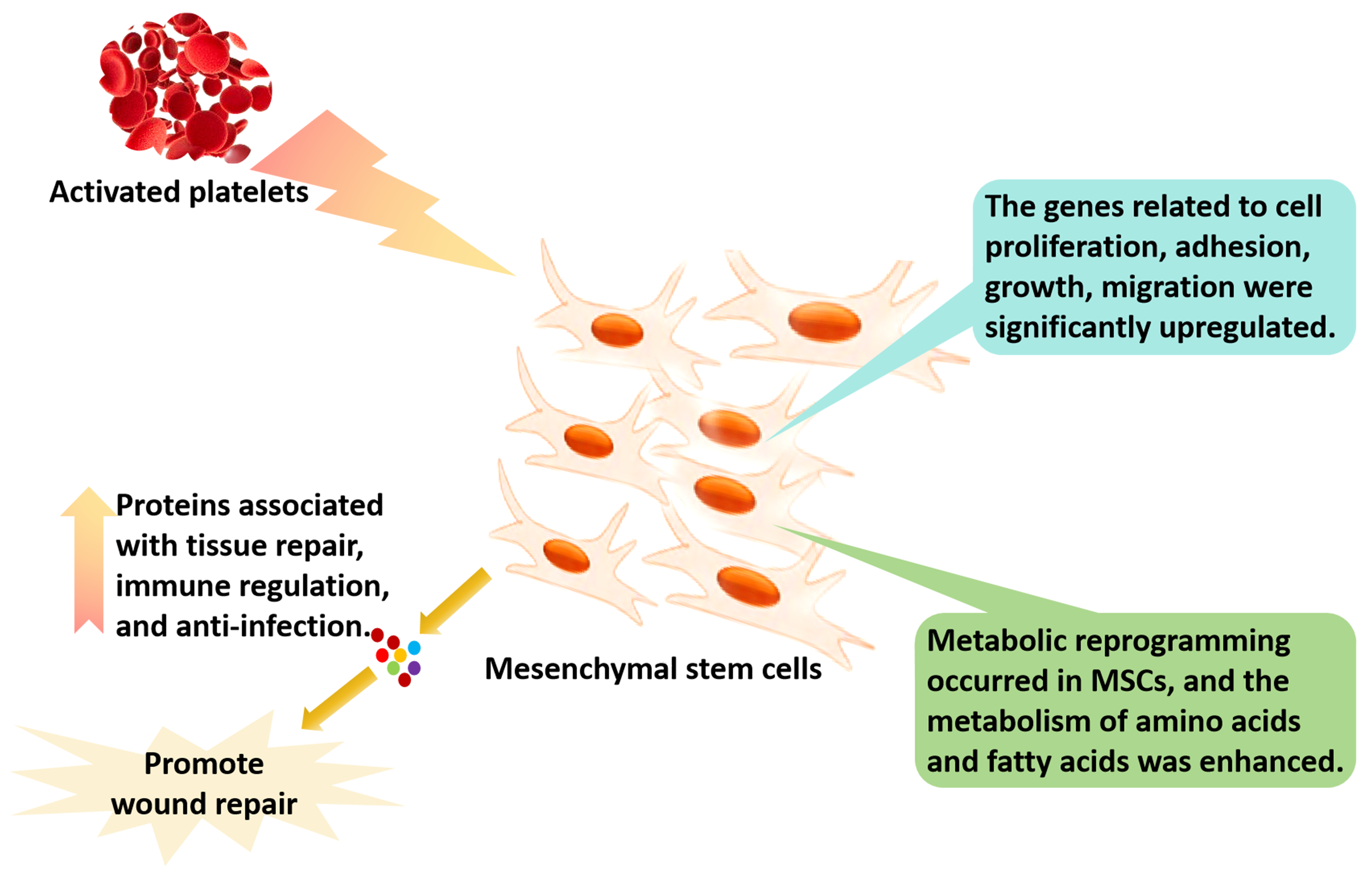

Supplement: Supplementary file 1 — Supplementary Material 1. [file 12864_2024_10329_MOESM1_ESM.zip › Supplementary document-38 Graphical abstract.tif]
